# Strain and Complexity, Passerini and Ugi Reactions of Four‐Membered Heterocycles and Further Elaboration of TOSMIC Product

**DOI:** 10.1002/open.202200083

**Published:** 2023-08-07

**Authors:** Gábor Sztanó, Zoltán Dobi, Tibor Soós

**Affiliations:** ^1^ Institute of Organic Chemistry Research Centre for Natural Sciences Budapest 1519 Hungary; ^2^ Hevesy György PhD School of Chemistry ELTE Eötvös Loránd University Budapest 1117 Pázmány Péter sétány 1/A Hungary

**Keywords:** Multicomponent reaction, Ugi-Passerini reaction, Oxetane, Azetidine, TOSMIC

## Abstract

Straightforward and general Passerini and Ugi procedures have been developed to incorporate four‐membered heterocycles into highly functionalized scaffolds. Additionally, toslymethyl isocyanide (TosMIC)‐derived Ugi adducts have been prepared, showcasing the prospect of the multicomponent reaction.

## Graphical abstract

Straightforward Passerini and Ugi procedures have been developed to incorporate four‐membered oxo‐heterocycles into highly decorated peptide‐like scaffolds. Then, toslymethyl isocyanide (TosMIC)‐derived Ugi adduct have been further prepared, showcasing the prospect of these multicomponent reactions.

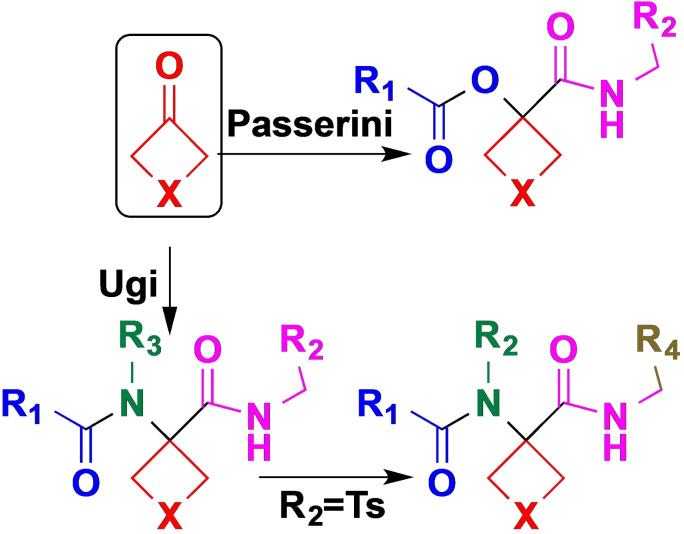

## Introduction

Over the last decade, four‐membered heterocycles have evolved into a prominent tactical building block in drug design and development.[^1^, ^2^] Incorporation of these structural motifs in drug candidates can beneficially alter various ADME properties, including but not limited to hERG liability reduction, LogD and p*K*_a_ modulations.[^3^, ^4^, ^5^, ^6^, ^7^] Furthermore, these structural elements have been frequently applied to provide new intellectual property space through scaffold hops[^8^, ^9^, ^10^] or, in certain cases, bioisosteric replacements for functional groups.[^11^, ^12^, ^13^, ^14^, ^15^, ^16^] As a result, considerable interest has been garnered to develop synthetic methods for the rapid assemblies of these four‐membered heterocyclic rings.[^17^, ^18^, ^19^] In parallel, based on cheap and commercially available 3‐oxo derivatives, a widely applicable modular synthetic approach has been introduced by Carriera, Rogers‐Evans and Müller to complement drug development.[^20^, ^21^, ^22^] Using these keto building blocks, research activities have also brought a range of synthetic efforts to access various rigid bicyclic scaffolds.[^23^, ^24^, ^25^] Although encouraging progress has been witnessed using this modular approach, the obvious application of these building blocks in multicomponent reactions (MCR) are scarce.[^26^, ^27^, ^28^, ^29^, ^30^] Herein, we report the realization of this prospect in Passerini and Ugi reactions. Moreover, not only were general procedures developed, but the utilization of tosylmethyl isocyanide (TosMIC) in these reactions was also described to allow for further synthetic applications of the corresponding Ugi products.

Multicomponent reactions are broadly utilized tools for the rapid generation of structural complexity. Arguably, Passerini and Ugi reactions and their recent variations are among the most important MCRs,[^31^, ^32^, ^33^, ^34^, ^35^, ^36^] and they are often used in pharmaceutical industry for lead discovery and optimization. These powerful reactions involve the convergent condensation of a carbonyl compound, an isocyanide, a carboxylic acid and an additional amine in case of the Ugi reaction. Despite their versatility, there are some limitations that become apparent from the corresponding literature. For example, while aldehydes are widely used carbonyl components in these reactions, there are relatively few examples reporting the use of ketones, especially the targeted four‐membered heterocyclic ketones, as inputs.[^28^, ^29^, ^30^] Additionally, the scope of commercially available isocyanides is rather restricted. To circumvent this limitation and use the MCR reaction products for further transformations, several so‐called convertible isocyanides have been introduced.[^37^, ^38^]

In a dual attempt to further the MCRs of four‐membered heterocyclic ketones regarding scope and practicality, we aimed to develop general, one‐pot procedures for isocyanide‐based Ugi and Passerini MCRs and utilize a specific isocyanide component that allows the further transformation of the condensation products. Along these lines, we focused on the utilization of tosylmethyl isocyanide (TosMIC) as the isocyanide component. We assumed that TosMIC, a multipurpose synthon that was introduced and further developed by van Leusen,[^39^, ^40^, ^41^, ^42^] is not only a unique, versatile and less odorous isocyanide, but its MCR adduct can furthermore be selectively transformed under benign conditions.[^43^, ^44^, ^45^, ^46^, ^47^, ^48^]

## Results and Discussion

Our initial focus was on the Passerini reaction of 3‐oxoazetidine derivatives. We quickly ascertained that azetidine **1**, benzoic acid (**2**) and tosylmethyl isocyanide (TosMIC, **3**) furnished the desired MCR product, although the reaction required 10 days in refluxing DCM to reach full conversion. After some experimentation, we found that performing the reaction in apolar solvents provided Passerini adduct **4 a** at higher conversions. (Table 1, entries 4–7 vs. entries 1, 2 and 9). However, there were exceptions to this trend, as the reaction was found kinetically favored in MeCN, but disfavored in EtOAc (Table 1, entries 3 and 9). As expected,^[49]^ this Passerini reaction was also not favored in protic and polar solvents like methanol (Table 1, entry 4). To our delight, there was no need to employ inert conditions and the reaction proceeded well in technical grade solvents. Notably, all reactions conducted at 40 °C were highly selective towards the formation of **4 a**, and no side products could be observed by ^1^H NMR spectroscopy. The results also show that increased concentration (Table 1, entries 12 vs. 9 vs. 13) and the excess of reagents **2** and **3** (Table 1, entry 13 vs. 14) facilitated the reaction. Additionally, higher reaction temperatures (Table 1, entries 10 vs. 9 vs. 11) or prolonged reaction times afforded higher conversions (Table 1 entry 14 vs. 15), up to 95 %. Although the reaction was considerably more facile at elevated temperature (80 °C), the reaction was less selective and the isolated yield decreased (Table 1 entry 15 vs. 16).

**Table 1:** Optimization of Passerini reaction condition to afford **4 a**.^[a]^

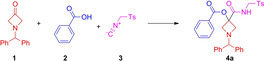						
Entry	Solvent	Temp. [°C]	Conc.^[b]^ [M]	Eq.^[c]^	Time [h]	Conv.^[d]^ [%]
**1**	DCM	40	0.2	1.5	20	34
**2**	DCE	40	0.2	1.5	20	33
**3**	MeCN	40	0.2	1.5	20	32
**4**	MeOH	40	0.2	1.5	20	20
**5**	DMSO	40	0.2	1.5	20	11
**6**	DMF	40	0.2	1.5	20	15
**7**	THF	40	0.2	1.5	20	12
**8**	EtOAc	40	0.2	1.5	20	22
**9**	PhMe	40	0.2	1.5	20	41
**10**	PhMe	25	0.2	1.5	20	22
**11**	PhMe	80	0.2	1.5	20	68
**12**	PhMe	40	0.067	1.5	20	19
**13**	PhMe	40	0.6	1.5	20	67
**14**	PhMe	40	0.6	2.0	20	89 (70)
**15**	PhMe	80	0.6	2.0	48	96 (55)
**16**	PhMe	40	0.6	2.0	48	95 (76)

[a] All reactions were conducted at 0.5 mmol scale of **1**. [b] Concentration of **1**. [c] Molar equivalents of **2** and **3**. [d] Conversion determined by NMR, isolated yield in parentheses.

**Scheme 1:**
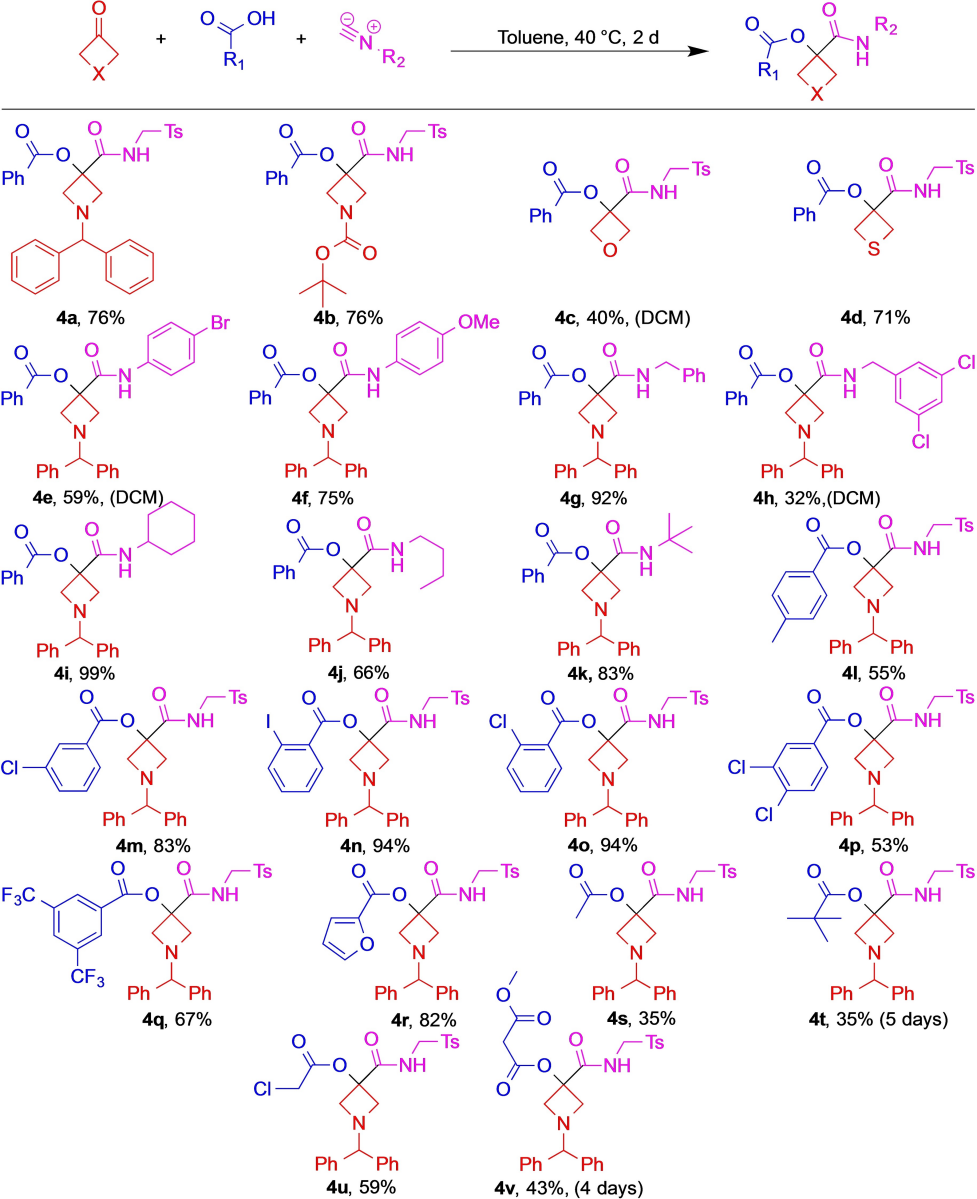
Scope of the Passerini three‐component reaction (Reaction conditions: Ketone (1.0 mmol, 1.0 equiv.), acid (2.0 equiv.), isocyanide (2.0 equiv.), toluene (0.6 M) under air. Reaction time: 48 h; temperature: 40 °C. Isolated yields. Deviation from standard conditions (solvent in cases of poor solubility, and reaction time in cases of low reactivity) indicated in parentheses).

With the optimal conditions in hand, we began to explore the scope of the reaction (Scheme 1). We found that all available azetidine and oxetane ketones afforded the desired products (**4 a**–**c**). Even the thietane analog **4 d** could be synthesized from the easily degradable thietane ketone. When the ketone reactant was insoluble in toluene, DCM was used (e. g., **4 c**) to effect the desired reaction. Next, selected aliphatic or aromatic isocyanide components were evaluated in the model Passerini reaction. Independently of the isocyanide used, the expected adducts were formed in good to high yields (**4 g**, **4 i** and **4 k** but also **4 e** or **4 j**). For solubility reasons regarding the isocyanide component, the synthesis of **4 h** had to be conducted in DCM which resulted in a mediocre yield. Finally, the acid component was varied. As a general trend, stronger aromatic acids provided the adducts in higher yields (Scheme 1, **4 m**–**o** vs. **4 a**). As expected, the less acidic aliphatic carboxylic acids resulted in lower yields (**4 s**–**v**).

**Scheme 2:**
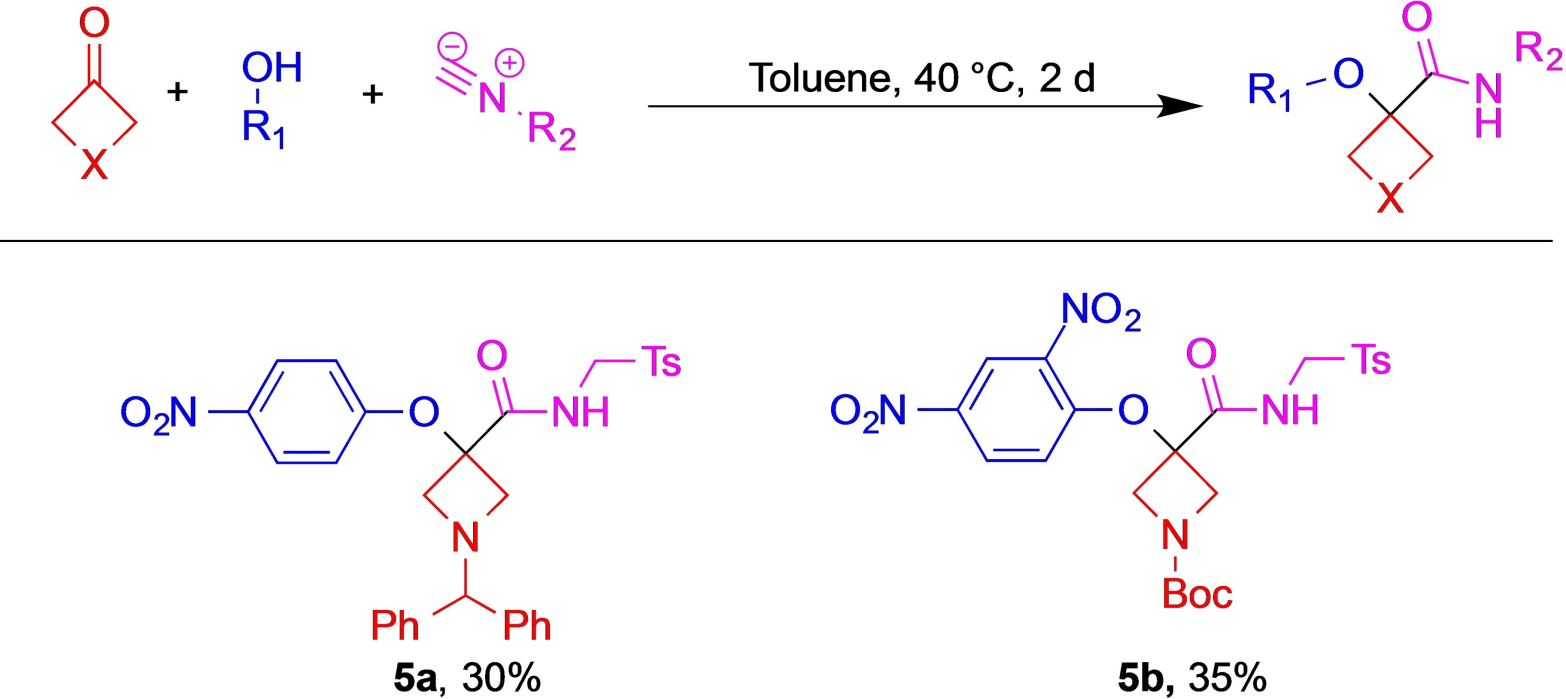
Extension toward Passerini‐Smiles reaction.

Next, we probed whether the reaction could be extended toward Passerini–Smiles MCR. Thus, based on El Kaïm's work,^[50]^ we used phenols instead of carboxylic acids. To our delight, the desired adducts **5 a, b** formed if appropriately acidic nitro‐phenols were employed, even though only mediocre yields (Scheme 2) were obtained. Notably, we were unable to synthesize the MCR adduct when less acidic phenols were employed.

Followingly, we turned our attention to the Ugi four‐component reaction to afford peptide‐like scaffolds. However, we found that under identical conditions, only the competing Passerini product was formed. We assumed that the required imine intermediate formation was hampered and the process halted at the hemiaminal stage due to the ring strain.[^51^, ^52^] To facilitate the imine formation from the hemiaminal and activate the imine intermediate,^[53]^ we probed various polar and protic solvents. Gratifyingly, we could completely shift the reaction towards Ugi product formation in methanol. Finally, trifluoroethanol proved to be the optimal solvent in this four‐component MCR and under conditions identical to those for the above Passerini reaction, azetidinone **1** afforded the Ugi product **6 a**.

**Scheme 3:**
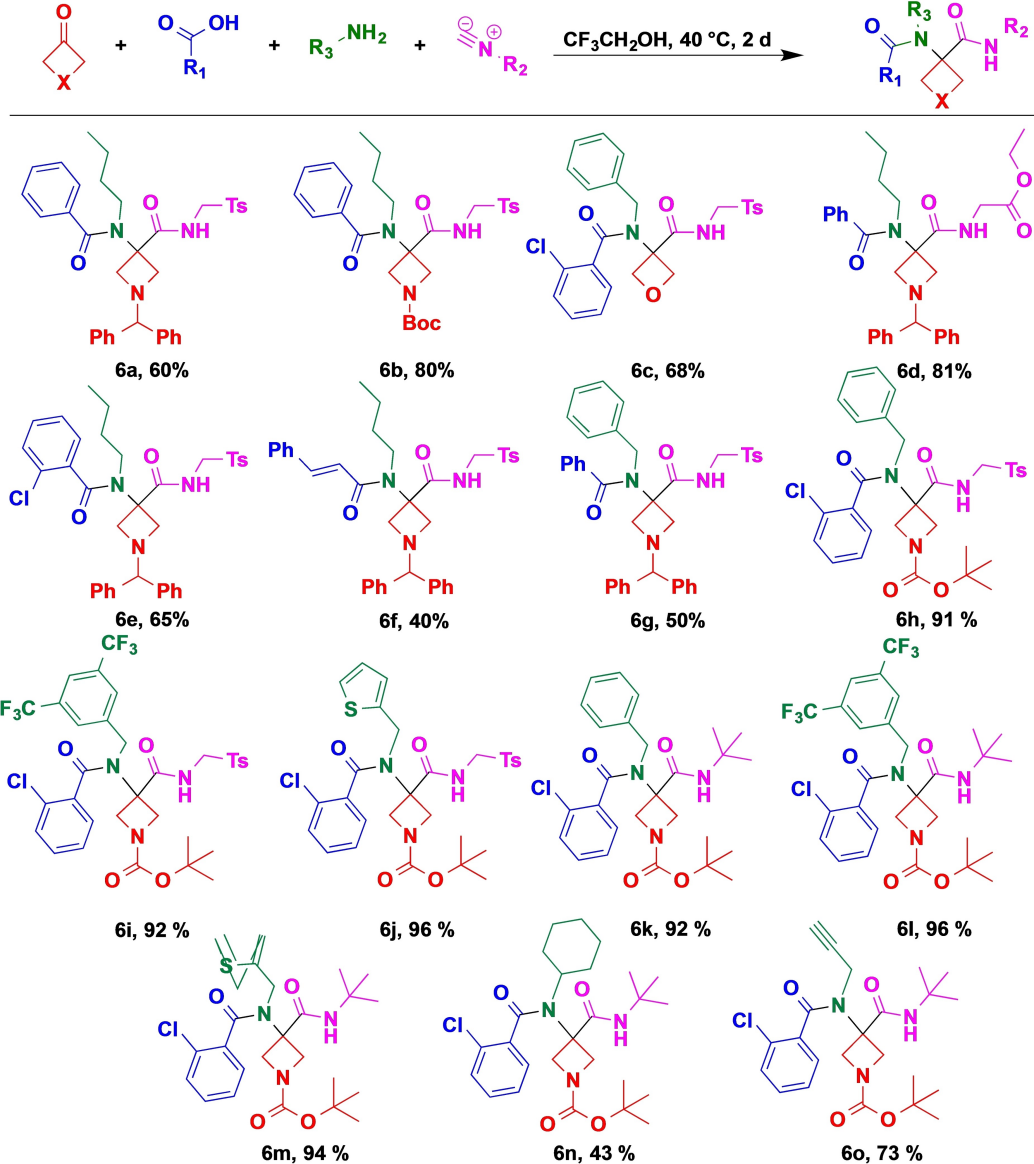
Scope of the Ugi four‐component reaction (Reaction conditions: Ketone (1.0 mmol, 1.0 equiv.), amine (2.0 equiv.), acid (2.0 equiv.), isocyanide (2.0 equiv.), CF_3_CH_2_OH (0.6 M) under air. Reaction time: 48 h; temperature: 40 °C. Isolated yields).

Next, we began to explore the scope of the reaction. During the course of this study, we found that all four components can be varied without significant loss of yield or selectivity (Scheme 3). Interestingly, the N‐Boc azetidine ketone performed noticeably better than the benzhydryl analogue (**6 b** vs. **6 a**). A similar trend between the acidity of the aromatic carboxylic acid component and the yield as for the Passerini reaction was observed (see **6 a** vs. **6 e** and **6 f**), although this effect was less pronounced here. Gratifyingly, variation of the isocyanide (**6 d**, **6 k**–**6 o**) or the amine component (**6 h**–**j**, **6 l**–**o**) had a negligible effect on the yield; only increased steric hindrance seems to have a detrimental effect (**6 n**).

**Scheme 4:**
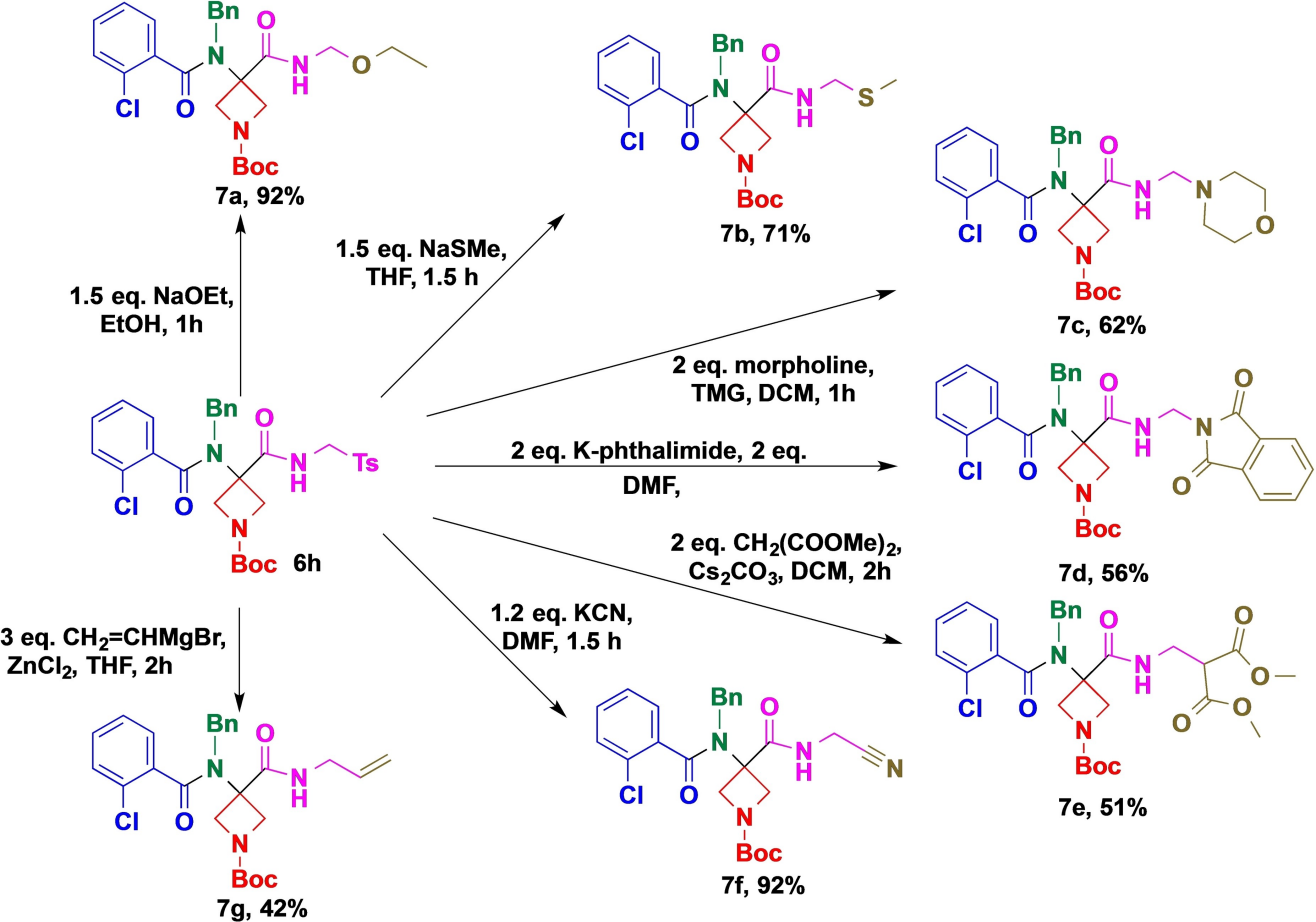
Further synthetic elaboration of the TosMIC adduct **6 h**.

Finally, our efforts were directed at exploring the transformation of Ugi adducts as a tool for further complexity generation (Scheme 4). More specifically, we targeted the TosMIC adduct **6 h**, as we envisioned that the tosyl group can be used as a synthetic handle for further transformations.[^43^, ^44^, ^45^, ^46^, ^47^, ^48^] Thus, we attempted to selectively react azetidine compounds **6 h** with various O, N and C‐based nucleophiles. We were pleased to find that the tosyl group acted as an efficient leaving group, allowing the introduction of those nucleophiles under benign conditions, often without any detectable side reactions. We observed that alkoxide, thiolate and cyanide (**7 a**, **7 b** and **7 f**) were highly active and selective reagents, allowing the introduction of those functionalities in high yields. Besides cyanide, other easily available carbon nucleophiles were probed. While those reactions resulted in mediocre yields (**7 e** and **7 g**), the compatibility of those reagents, especially the Grignard reagent, indicates a promising tool for further synthetic application of TosMIC‐based Ugi adducts.

## Conclusions

To conclude, we demonstrated that four‐membered heterocyclic ketones are excellent substrates for Passerini and Ugi reactions, providing simple routes to generate pharmaceutically relevant scaffolds. These MCR reactions proceed in good to high yields with broad range of components. We also showed that TosMIC is not only a suitable isocyanide component, but also a convertible reagent that allow the further synthetic application of the Ugi adduct.

## Supporting Information

Detailed experimental procedures and analytical data can be found in the Supporting Information for this article, which is available under https://doi.org/10.1002/open.202200083.

## Conflict of interest

The authors declare no conflict of interest.

## Supporting information

As a service to our authors and readers, this journal provides supporting information supplied by the authors. Such materials are peer reviewed and may be re‐organized for online delivery, but are not copy‐edited or typeset. Technical support issues arising from supporting information (other than missing files) should be addressed to the authors.

Supporting Information
