# Supplementary material for: Strain and Complexity, Passerini and Ugi Reactions of Four‐Membered Heterocycles and Further Elaboration of TOSMIC Product
Source: ChemistryOpen. 2023 Aug 7;12(8):e202200083. doi: 10.1002/open.202200083 (PMC10405247; doi:10.1002/open.202200083)

ChemistryOpen

Supporting Information

Strain and Complexity, Passerini and Ugi Reactions of Four-Membered Heterocycles and Further Elaboration of TOSMIC Product

Gábor Sztanó, Zoltán Dobi, and Tibor Soós*

General Information

NMR spectra were acquired on Varian spectrometer, running at 500 MHz and 125 MHz for ^{1}H and ^{13}C, respectively. Chemical shifts (δ) are reported in ppm relative to residual solvent signals (^{1}H, $CHCl_3$: δ = 7.26, DMSO: δ = 2.50; ^{13}C: $CHCl_3$: δ = 77.16, DMSO: δ =39.52). The following abbreviations are used to indicate the multiplicity in spectra: s, singlet; d, doublet; t, triplet; m, multiplet. ^{13}C NMR spectra were acquired on a broad band decoupled mode. High resolution mass spectra were obtained on a Q-TOF Premier mass spectrometer (Waters Corporation) in positive electrospray mode. Melting points were determined on a SRS MPA100 apparatus and are uncorrected. Column chromatography was carried out with Teledyne ISCO CombiFlash Rf200 UV/VIS system, by using RediSep Rf Gold® Normal-phase Silica columns and hexane/ethyl-acetate gradient elution program unless otherwise noted. Commercially available materials were purchased from Sigma-Aldrich and Fluorochem; all were used without further purification. Ether type solvents and toluene were freshly distilled from sodium/benzophenone.

Preparation of isocyanides

1-bromo-4-isocyanobenzene and ethyl 2-isocyanoacetate were prepared according to literature procedures, and their spectras (^{1}H and ^{13}C) match the reported ones[1,2].

N-(3,5-dichlorobenzyl)formamide, SI1

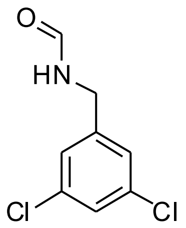

3,5-dichlorobenzaldehyde(25.0 mmol, 4.38 g), formamide (300 mmol, 13.51 g, 11.93 ml) and formic acid (200 mmol, 9.21 g, 7.55 ml) was stirred for 2.5 hours at 180 °C with a Dean-Stark apparatus. The reaction mixture was then poured on ice, stirred for further 2 hours at room temperature, then filtered, yielding 4.62 g crude product as orange crystals. Recrystallization from IPA afforded 2.39 g (47%) faint orange crystals.

M.p.: 96.8 °C

^{1}H NMR (500 MHz, $CDCl_3$) δ 8.30 (s, 1H), 7.28 (t, *J* = 1.8 Hz, 1H), 7.18 (dd, *J* = 1.2, 0.5 Hz, 2H), 5.95 (bs, 1H), 4.45 (d, *J* = 6.2 Hz, 2H)

^{13}C NMR (126 MHz, $CDCl_3$) 161.6, 141.1, 135.2, 127.7, 125.9, 41.0.

HR-MS (ESI): *m/z* $[M+H]^+$ calcd for $C_8H_7Cl_2NO$: 203.9983; found: 203.9985.

1,3-dichloro-5-(isocyanomethyl)benzene, SI2

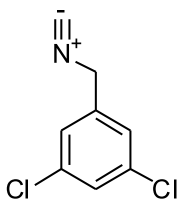

N-(3,5-dichlorobenzyl)formamide (7.0 mmol, 1.43 g) and TEA (21.0 mmol, 2.12 g, 2.93 ml) were dissolved in 15 ml of DCM, cooled to 0 °C and $POCl_3$ (8.4 mmol, 1.29 g, 0.78 ml) was slowly added. After 4 hours it was washed with sat. $NaHCO_3$ and water. The solvent was evaporated under reduced pressure, then heptane was added and again concentrated to give 1.58 g (>100%) dark brown, foul smelling liquid. The compound suffered decay under column chromatography conditions, thus it was used further without purification.

M.p.: Not measured.

^{1}H NMR (500 MHz, $CDCl_3$) δ 7.36 (s, 1H), 7.26 – 7.24 (m, 2H), 4.62 (s, 2H)

General procedure for the Passerini reaction

The heteocyclic ketone (1.0 mmol), acid component (2.0 mmol) and the isocyanide component (2.0 mmol) were dissolved in 1.67 ml of toluene, and stirred at 40 °C for two days. The mixture was concentrated under reduced pressure and subjected to column chromatography (hexane-ethyl acetate gradient elution). In the case of the aggressive smelling isocyanides, after two days 5 ml of 1M HCl was added and the two phase system was vigorously stirred for 30 min, then the phases was separated, the organic phase was concentrated under reduced pressure and subjected to column chromatography.

1-benzhydryl-3-((tosylmethyl)carbamoyl)azetidin-3-yl benzoate, 4a

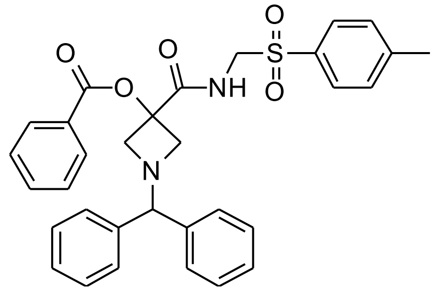

3.0 mmol scale. 1.186 g (71%) white solid.

M.p.: 158.1 °C.

^{1}H NMR (500 MHz, $CDCl_3$) δ 8.46 (t, *J* = 6.4 Hz, 1H), 7.95 (d, *J* = 7.2 Hz, 2H), 7.79 (d, *J* = 8.2 Hz, 2H), 7.56 (t, *J* = 7.5 Hz, 1H), 7.47 (d, *J* = 7.2 Hz, 4H), 7.41 (t, *J* = 7.8 Hz, 2H), 7.36 (t, *J* = 7.6 Hz, 4H), 7.26 (dd, *J* = 20.0, 7.7 Hz, 4H), 4.84 (d, *J* = 6.5 Hz, 2H), 4.46 (s, 1H), 3.70 (dd, *J* = 7.2, 2.1 Hz, 2H), 3.30 (dd, *J* = 7.4, 1.9 Hz, 2H), 2.40 (s, 3H).

^{13}C NMR (126 MHz, $CDCl_3$) δ 168.8, 164.9, 145.6, 140.4, 133.8, 133.5, 129.9, 129.8, 128.8, 128.8, 128.6, 128.3, 127.7, 127.3, 77.7, 72.8, 62.6, 60.0, 21.6.
HR-MS (ESI): *m/z* $[M+H]^+$ calcd for $C_{32}H_{31}N_2O_5S$: 555.1954; found: 555.1958.

tert-butyl 3-(benzoyloxy)-3-((tosylmethyl)carbamoyl)azetidine-1-carboxylate, 4b

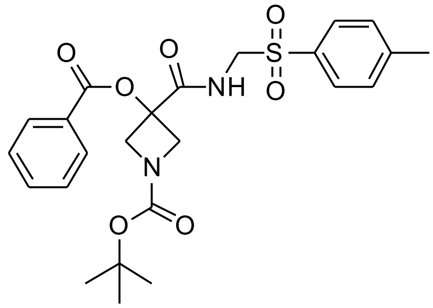

3.0 mmol scale. 1.117 g (76%) white solid.
M.p.: 180.0 °C.
^1H NMR (500 MHz, $CDCl_3$) δ 8.07 (d, *J* = 7.2 Hz, 2H), 7.75 (d, *J* = 8.3 Hz, 2H), 7.67 (t, *J* = 7.5 Hz, 1H), 7.52 (t, *J* = 7.8 Hz, 2H), 7.34 (d, *J* = 8.0 Hz, 2H), 6.65 (t, *J* = 6.7 Hz, 1H), 4.68 (d, *J* = 6.1 Hz, 2H), 4.27 (bs, 2H), 4.08 (d, *J* = 10.0 Hz, 2H), 2.45 (s, 3H), 1.43 (s, 9H).
^{13}C NMR (126 MHz, $CDCl_3$) δ 168.0, 165.0, 155.7, 145.6, 134.3, 133.8, 130.1, 129.9, 128.8, 128.7, 128.0, 80.3, 74.4, 60.2, 57.6, 28.2, 21.7
HR-MS (ESI): *m/z* $[M+Na]^+$ calcd for $C_{24}H_{28}N_2O_7SNa$: 511.1515; found: 511.1520.

3-((tosylmethyl)carbamoyl)oxetan-3-yl benzoate, 4c

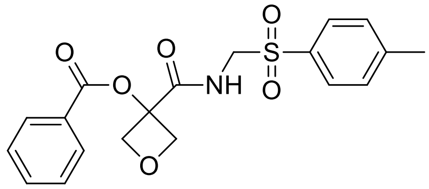

The reaction was carried out in DCM due to the poor solubility of oxetane in toluene. 0.156 g (40%) white solid.
M.p.: 179.9 °C.
^1H NMR (500 MHz, $CDCl_3$) δ 8.12 – 8.04 (m, 2H), 7.76 (d, *J* = 8.3 Hz, 2H), 7.68 (t, *J* = 7.5 Hz, 1H), 7.53 (t, *J* = 7.8 Hz, 2H), 7.33 (d, *J* = 8.1 Hz, 2H), 6.78 (t, *J* = 6.6 Hz, 1H), 4.88 (d, *J* = 8.2 Hz, 2H), 4.81 (d, *J* = 8.1 Hz, 2H), 4.72 (d, *J* = 6.8 Hz, 2H), 2.45 (s, 3H).
^{13}C NMR (126 MHz, DMSO-d6) δ 172.9, 169.6, 149.8, 140.0, 139.1, 135.1, 134.9, 134.4, 133.9, 133.8, 133.73, 133.68, 83.3, 82.0, 65.8, 26.3.
HR-MS (ESI): *m/z* $[M+H]^+$ calcd for $C_{19}H_{20}NO_6S$: 390.1011; found: 390.1021.

3-((tosylmethyl)carbamoyl)thietan-3-yl benzoate, 4d

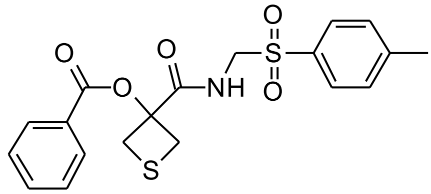

0.282 g (70%) white solid.
M.p.: 181.2 °C.
^1H NMR (500 MHz, $CDCl_3$) δ 8.02 (dd, *J* = 8.2, 1.0 Hz, 2H), 7.79 (d, *J* = 8.2 Hz, 2H), 7.63 (t, *J* = 7.5 Hz, 1H), 7.48 (t, *J* = 7.8 Hz, 2H), 7.37 (t, *J* = 6.9 Hz, 1H), 7.31 (d, *J* = 8.0 Hz, 2H), 4.78 (d, *J* = 6.7 Hz, 2H), 3.75 (dd, *J* = 9.8, 1.7 Hz, 2H), 3.55 (dd, *J* = 9.9, 1.6 Hz, 2H), 2.44 (s, 3H).
^{13}C NMR (126 MHz, DMSO-d6) δ 173.5, 169.0, 149.7, 140.0, 139.0, 135.0, 134.84, 134.0, 133.8, 133.6, 86.1, 65.9, 38.7, 26.3.
HR-MS (ESI): *m/z* $[M+H]^+$ calcd for $C_{19}H_{20}NO_5S_2$: 406.0783; found: 406.0772.

1-benzhydryl-3-((4-bromophenyl)carbamoyl)azetidin-3-yl benzoate, 4e

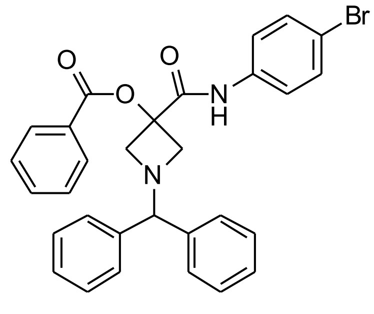

The reaction was carried out in DCM. 0.320 g (59%) white solid.
M.p.: 212.8 °C.
^1H NMR (500 MHz, $CDCl_3$) δ 9.86 (s, 1H), 8.06 (dd, *J* = 8.3, 1.2 Hz, 2H), 7.67 – 7.54 (m, 3H), 7.54 – 7.38 (m, 8H), 7.34 (t, *J* = 7.5 Hz, 4H), 7.28-7.22 (m, 2H), 4.56 (s, 1H), 3.98 (dd, *J* = 7.2, 2.2 Hz, 2H), 3.47 (dd, *J* = 7.3, 2.0 Hz, 2H).
^{13}C NMR (126 MHz, $CDCl_3$) δ 167.3, 165.3, 140.4, 137.1, 133.6, 132.1, 130.0, 128.9, 128.5, 127.8, 127.2, 121.2, 116.8, 77.6, 73.3, 63.0.
HR-MS (ESI): *m/z* $[M+H]^+$ calcd for $C_{30}H_{26}BrN_2O_3$: 541.1127; found: 541.1136.

1-benzhydryl-3-((4-methoxyphenyl)carbamoyl)azetidin-3-yl benzoate, 4f

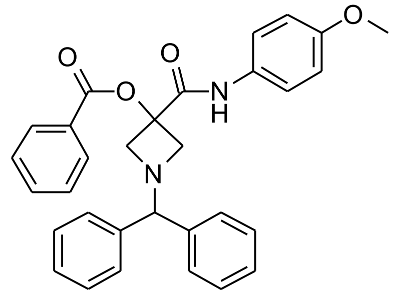

0.372 g (75%) white solid.
M.p.: 162.3 °C.
^1H NMR (500 MHz, $CDCl_3$) δ 9.61 (s, 1H), 8.07 (d, *J* = 8.0 Hz, 2H), 7.62 – 7.55 (m, 3H), 7.40 – 7.47 (m, 6H), 7.33 (t, *J* = 7.5 Hz, 4H), 6.93 (d, *J* = 9.0 Hz, 2H), 4.56 (s, 1H), 3.99 (dd, *J* = 7.2, 2.2 Hz, 2H), 3.82 (s, 3H), 3.47 (dd, *J* = 7.2, 2.1 Hz, 2H),
^{13}C NMR (126 MHz, $CDCl_3$) δ 167.0, 165.4, 156.4, 140.6, 133.4, 131.2, 130.0, 129.2, 128.8, 128.4, 127.7, 127.2, 121.6, 114.3, 77.6, 73.4, 63.1, 55.5.

HR-MS (ESI): *m/z* $[M+H]^+$ calcd for $C_{31}H_{29}N_2O_4$: 493.2127; found: 493.2123.

1-benzhydryl-3-(benzylcarbamoyl)azetidin-3-yl benzoate, 4g

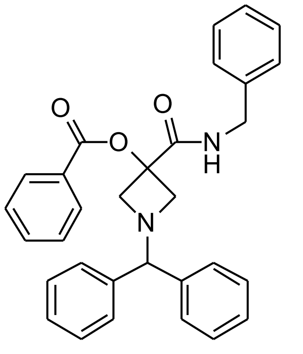

0.437 g (92%), white solid.
M.p.: 73.7 °C.
^{1}H NMR (500 MHz, $CDCl_3$) δ 8.05 (dd, *J* = 8.3, 1.1 Hz, 2H), 7.75 (t, *J* = 5.3 Hz, 1H), 7.57 (t, *J* = 7.4 Hz, 1H), 7.47 – 7.31 (m, 7H), 7.31-7.17 (m, 10H), 4.61 (d, *J* = 5.5 Hz, 2H), 4.49 (s, 1H), 3.87 (dd, *J* = 7.4, 2.1 Hz, 2H), 3.39 (dd, *J* = 7.4, 1.9 Hz, 2H).
^{13}C NMR (126 MHz, $CDCl_3$) δ 169.3, 165.4, 140.8, 138.3, 133.4, 130.0, 129.3, 128.8, 128.7, 128.4, 127.7, 127.6, 127.5, 127.2, 77.4, 73.6, 62.9, 43.6.
HR-MS (ESI): *m/z* $[M+H]^+$ calcd for $C_{31}H_{29}N_2O_3$: 477.2178; found: 477.2183.

1-benzhydryl-3-((3,5-dichlorobenzyl)carbamoyl)azetidin-3-yl benzoate, 4h

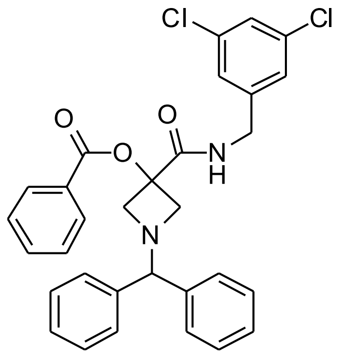

The reaction was carried out in DCM. 0.174 g (32%) yellowish solid.
M.p.: 79.9 °C.
^{1}H NMR (500 MHz, $CDCl_3$) δ 8.08 (d, *J* = 7.5 Hz, 2H), 7.80 (t, *J* = 5.2 Hz, 1H), 7.59 (t, *J* = 7.4 Hz, 1H), 7.46 (t, *J* = 7.7 Hz, 2H), 7.36 (d, *J* = 7.3 Hz, 4H), 7.35 – 7.29 (m, 5H), 7.28-7.21 (m, 4H), 4.56 (d, *J* = 5.8 Hz, 2H), 4.53 (s, 1H), 3.90 (d, *J* = 9.4 Hz, 2H), 3.43 (d, *J* = 9.4 Hz, 2H)
^{13}C NMR (126 MHz, $CDCl_3$) δ 169.6, 165.4, 141.8, 140.7, 135.4, 133.5, 130.0, 129.1, 128.8, 128.4, 127.7, 127.6, 127.2, 125.7, 77.5, 73.5, 62.8, 42.5.
HR-MS (ESI): *m/z* $[M+H]^+$ calcd for $C_{31}H_{27}Cl_2N_2O_3$: 545.1399; found: 545.1396.

1-benzhydryl-3-(cyclohexylcarbamoyl)azetidin-3-yl benzoate, 4i

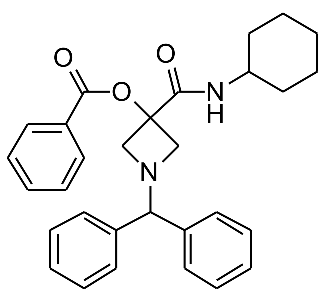

0.466 g (99%) white solid.
M.p.: 181.8 °C.
^{1}H NMR (500 MHz, $CDCl_3$) δ 8.05 (dd, *J* = 8.1, 1.0 Hz, 2H), 7.56 (t, *J* = 7.5 Hz, 1H), 7.46 – 7.36 (m, 7H), 7.31 (t, *J* = 7.5 Hz, 4H), 7.23 (t, *J* = 7.3 Hz, 2H), 4.51 (s, 1H), 3.96 – 3.87 (m, 1H), 3.85 (dd, *J* = 7.4, 2.1 Hz, 2H), 3.39 (dd, *J* = 7.5, 1.9 Hz, 2H), 2.02 – 1.94 (m, 2H), 1.75 (dd, *J* = 9.2, 4.4 Hz, 2H), 1.68 – 1.56 (m, 1H), 1.53 – 1.40 (m, 2H), 1.39-1.24 (m, 3H).
^{13}C NMR (126 MHz, $CDCl_3$) δ 168.5, 165.3, 141.0, 133.3, 130.0, 129.4, 128.7, 128.29, 127.5, 127.2, 77.5, 73.5, 63.0, 47.8, 32.8, 25.6, 24.4.
HR-MS (ESI): *m/z* $[M+H]^+$ calcd for $C_{30}H_{33}N_2O_3$: 469.2491; found: 469.2492.

1-benzhydryl-3-(butylcarbamoyl)azetidin-3-yl benzoate, 4j

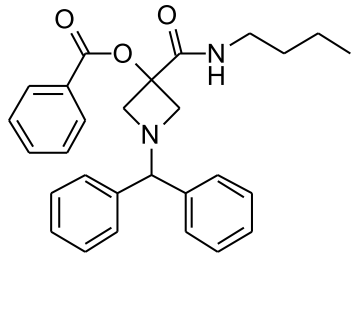

0.354 g (80%) white solid.
M.p.: 129.0 °C.
^{1}H NMR (500 MHz, $CDCl_3$) δ 8.10 (d, *J* = 7.5 Hz, 2H), 7.58 (t, *J* = 7.4 Hz, 1H), 7.50 – 7.38 (m, 7H), 7.34 (t, *J* = 7.5 Hz, 4H), 7.30 – 7.21 (m, 2H), 4.56 (s, 1H), 3.91 (d, *J* = 9.3 Hz, 2H), 3.43 (t, *J* = 6.6 Hz, 4H), 1.70 – 1.59 (m, 2H), 1.53 – 1.43 (m, 2H), 1.03 (t, *J* = 7.3 Hz, 3H),
^{13}C NMR (126 MHz, $CDCl_3$) δ 169.3, 165.4, 141.0, 133.3, 130.0, 129.4, 128.7, 128.4, 127.6, 127.3, 77.6, 73.6, 63.0, 39.2, 31.7, 20.1, 13.8.
HR-MS (ESI): *m/z* $[M+H]^+$ calcd for $C_{28}H_{31}N_2O_3$: 443.2335; found: 443.2329.

1-benzhydryl-3-(tert-butylcarbamoyl)azetidin-3-yl benzoate, 4k

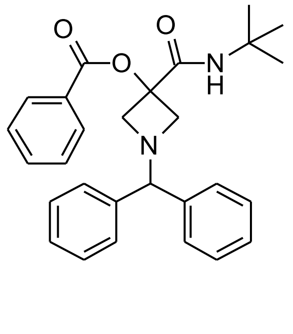

0.364 g (83%) white solid.
M.p.: 140.1 °C.
^{1}H NMR (500 MHz, $CDCl_3$) δ 8.08 – 8.02 (m, 2H), 7.55 (dd, *J* = 14.0, 6.4 Hz, 2H), 7.42 (t, *J* = 7.8 Hz, 2H), 7.39 (d, *J* = 7.3 Hz, 4H), 7.31 (t, *J* = 7.5 Hz, 4H), 7.23 (t, *J* = 7.3 Hz, 2H), 4.51 (s, 1H), 3.83 (dd, *J* = 7.3, 2.0 Hz, 2H), 3.37 (dd, *J* = 7.5, 1.8 Hz, 2H), 1.47 (s, 9H)
^{13}C NMR (126 MHz, $CDCl_3$) δ 168.5, 165.2, 141.0, 133.3, 130.0, 129.4, 128.7, 128.3, 127.6, 127.2, 77.5, 73.5, 63.1, 51.1, 28.9

HR-MS (ESI): *m/z* $[M+H]^+$ calcd for $C_{28}H_{31}N_2O_3$: 443.2335; found: 443.2333.

1-benzhydryl-3-((tosylmethyl)carbamoyl)azetidin-3-yl 4-methylbenzoate, 4l

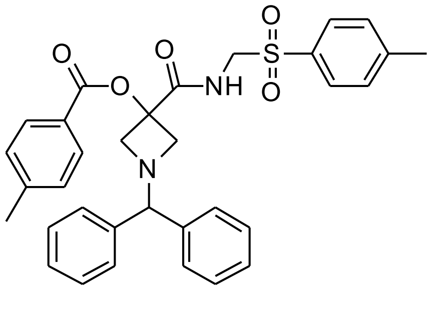

0.320 g (56%) white solid.
M.p.: 197.0 °C.
^{1}H NMR (500 MHz, $CDCl_3$) δ 8.41 (t, *J* = 6.4 Hz, 1H), 7.85 (d, *J* = 8.2 Hz, 2H), 7.79 (d, *J* = 8.2 Hz, 2H), 7.47 (d, *J* = 7.2 Hz, 4H), 7.36 (t, *J* = 7.6 Hz, 4H), 7.29 – 7.18 (m, 6H), 4.84 (d, *J* = 6.5 Hz, 2H), 4.46 (s, 1H), 3.69 (dd, *J* = 7.3, 2.1 Hz, 2H), 3.30 (dd, *J* = 7.4, 1.9 Hz, 2H), 2.40 (s, 6H).
^{13}C NMR (126 MHz, $CDCl_3$) δ 169.0, 165.0, 145.4, 144.4, 140.5, 133.9, 130.00, 129.95, 129.1, 128.8, 128.7, 127.7, 127.4, 126.1, 77.7, 72.9, 62.6, 60.1, 21.69, 21.67.
HR-MS (ESI): *m/z* $[M+H]^+$ calcd for $C_{33}H_{33}N_2O_5S$: 569.2110; found: 569.2118.

1-benzhydryl-3-((tosylmethyl)carbamoyl)azetidin-3-yl 3-chlorobenzoate, 4m

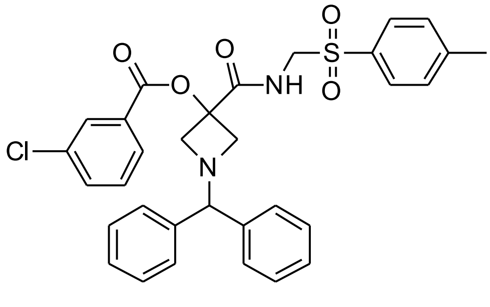

0.487 g (83%) white solid.
M.p.: 160.7 °C.
^{1}H NMR (500 MHz, $CDCl_3$) δ 8.55 (t, *J* = 6.4 Hz, 1H), 7.92 (t, *J* = 1.7 Hz, 1H), 7.83 (d, *J* = 7.8 Hz, 1H), 7.79 (d, *J* = 8.2 Hz, 2H), 7.53 (dd, *J* = 8.0, 1.0 Hz, 1H), 7.48 (d, *J* = 7.3 Hz, 4H), 7.37 (t, *J* = 7.7 Hz, 5H), 7.32-7.23 (m, 4H), 4.85 (d, *J* = 6.5 Hz, 2H), 4.47 (s, 1H), 3.70 (dd, *J* = 7.1, 2.2 Hz, 2H), 3.30 (dd, *J* = 7.3, 2.0 Hz, 2H), 2.41 (s, 3H).
^{13}C NMR (126 MHz, $CDCl_3$) δ ^{13}C δ 168.7, 163.7, 148.7, 145.5, 140.3, 134.6, 133.8, 133.5, 130.6, 130.0, 129.9, 129.7, 128.9, 128.6, 128.0, 127.8, 127.4, 77.8, 73.1, 62.7, 60.1, 21.7.
HR-MS (ESI): *m/z* $[M+H]^+$ calcd for $C_{32}H_{30}ClN_2O_5S$: 589.1564; found: 589.1569.

1-benzhydryl-3-((tosylmethyl)carbamoyl)azetidin-3-yl 2-iodobenzoate, 4n

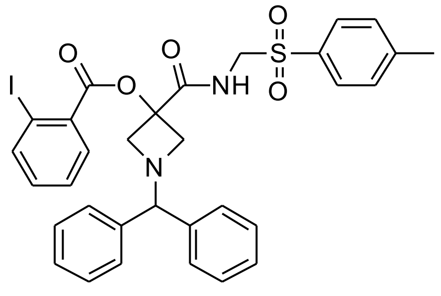

0.643 g (94%) white solid.
M.p.: 85.9 °C.
^{1}H NMR (500 MHz, $CDCl_3$) δ 8.55 (t, *J* = 6.4 Hz, 1H), 7.95 (d, *J* = 7.9 Hz, 1H), 7.81 (dd, *J* = 11.9, 4.9 Hz, 3H), 7.47 (d, *J* = 7.2 Hz, 4H), 7.36 (t, *J* = 7.6 Hz, 5H), 7.26 (dd, *J* = 16.2, 7.9 Hz, 4H), 7.13 (td, *J* = 7.7, 1.7 Hz, 1H), 4.86 (d, *J* = 6.5 Hz, 2H), 4.49 (s, 1H), 3.71 (dd, *J* = 7.2, 2.1 Hz, 2H), 3.36 (dd, *J* = 7.3, 2.0 Hz, 2H), 2.38 (s, 3H).
^{13}C NMR (126 MHz, $CDCl_3$) δ 168.6, 164.7, 145.5, 141.3, 140.3, 133.8, 133.6, 133.1, 131.6, 130.1, 128.9, 128.6, 127.9, 127.8, 127.4, 94.1, 77.6, 73.3, 62.5, 60.1, 21.7.
HR-MS (ESI): *m/z* $[M+H]^+$ calcd for $C_{32}H_{30}IN_2O_5S$: 681.0920; found: 681.0928.

1-benzhydryl-3-((tosylmethyl)carbamoyl)azetidin-3-yl 2-chlorobenzoate, 4o

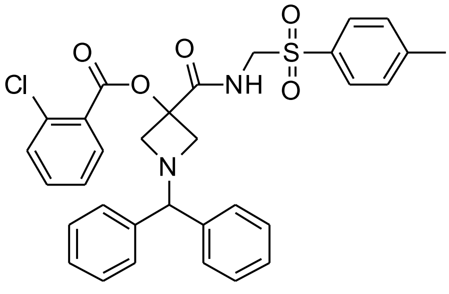

0.557 g (94%) white solid.
M.p.: 89.0 °C (decomposes).
^{1}H NMR (500 MHz, $CDCl_3$) δ 8.55 (t, *J* = 6.1 Hz, 1H), 7.84 (d, *J* = 7.7 Hz, 1H), 7.81 (d, *J* = 8.1 Hz, 2H), 7.49 (d, *J* = 7.4 Hz, 4H), 7.43 (d, *J* = 2.1 Hz, 2H), 7.38 (t, *J* = 7.5 Hz, 4H), 7.32 – 7.22 (m, 5H), 4.87 (d, *J* = 6.5 Hz, 2H), 4.49 (s, 1H), 3.71 (d, *J* = 9.1 Hz, 2H), 3.34 (d, *J* = 9.1 Hz, 2H), 2.40 (s, 3H)
^{13}C NMR (126 MHz, $CDCl_3$) δ 168.7, 163.8, 145.5, 140.3, 134.0, 133.8, 133.1, 132.0, 131.0, 130.0, 128.9, 128.6, 127.8, 127.4, 126.6, 77.7, 73.3, 62.6, 60.1, 21.7.
HR-MS (ESI): *m/z* $[M+H]^+$ calcd for $C_{32}H_{30}ClN_2O_5S$: 589.1564; found: 589.1567.

1-benzhydryl-3-((tosylmethyl)carbamoyl)azetidin-3-yl 3,4-dichlorobenzoate, 4p

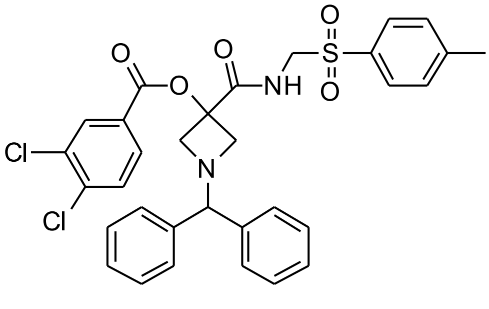

0.330 g (53%) white solid.
M.p.: 151.6 °C.
^{1}H NMR (500 MHz, $CDCl_3$) δ 8.58 (t, *J* = 6.5 Hz, 1H), 8.01 (d, *J* = 1.9 Hz, 1H), 7.79 (d, *J* = 8.2 Hz, 2H), 7.76 (dd, *J* = 8.4, 2.0 Hz, 1H), 7.50-

7.45 (m, 5H), 7.37 (t, *J* = 7.6 Hz, 4H), 7.33 – 7.22 (m, 4H), 4.84 (d, *J* = 6.5 Hz, 2H), 4.48 (s, 1H), 3.70 (dd, *J* = 7.2, 2.2 Hz, 1H), 3.31 (dd, *J* = 7.3, 2.0 Hz, 2H), 2.40 (s, 3H).
^{13}C NMR (126 MHz, $CDCl_3$) δ 168.6, 163.1, 145.5, 140.2, 138.2, 133.8, 133.0, 131.9, 131.7, 130.6, 130.4, 130.0, 129.0, 128.9, 128.7, 128.6, 127.9, 127.4, 77.7, 73.3, 62.5, 60.1, 21.7.
HR-MS (ESI): *m/z* $[M+H]^+$ calcd for $C_{32}H_{29}Cl_2N_2O_5S$: 623.1174; found: 623.1174.

1-benzhydryl-3-((tosylmethyl)carbamoyl)azetidin-3-yl 3,5-bis(trifluoromethyl)benzoate, 4q

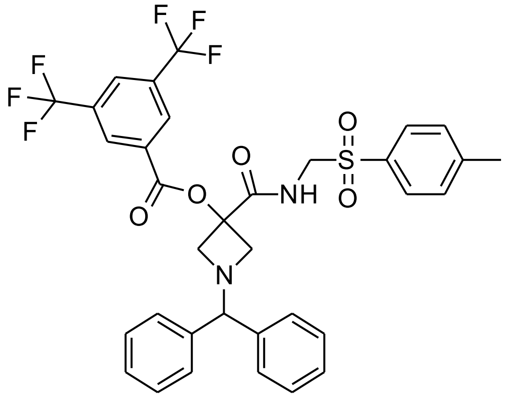

0.463 g (67%) white solid.
M.p.: 153.1 °C.
^{1}H NMR (500 MHz, $CDCl_3$) δ 8.71 (t, *J* = 5.9 Hz, 1H), 8.37 (s, 2H), 8.05 (s, 1H), 7.80 (d, *J* = 8.0 Hz, 2H), 7.48 (d, *J* = 7.4 Hz, 4H), 7.38 (t, *J* = 7.5 Hz, 4H), 7.32 – 7.23 (m, 4H), 4.85 (d, *J* = 6.4 Hz, 2H), 4.50 (s, 1H), 3.75 (d, *J* = 8.8 Hz, 2H), 3.37 (d, *J* = 8.7 Hz, 2H), 2.41 (s, 3H).
^{13}C NMR (126 MHz, $CDCl_3$) δ 168.4, 162.4, 145.6, 140.0, 133.8 , 132.3 (q, *J* = 34.1 Hz), 131.1, 130.3, 129.9 (q, *J* = 1.8 Hz), 129.4, 128.9, 128.6, 128.0, 127.4, 126.8 (q, *J* = 3.6 Hz), 122.7 (q, *J* =273.0 Hz), 77.8, 73.5, 62.6, 60.1, 21.6
HR-MS (ESI): *m/z* $[M+H]^+$ calcd for $C_{34}H_{29}F_6N_2O_5S$: 691.1701; found: 691.1694.

1-benzhydryl-3-((tosylmethyl)carbamoyl)azetidin-3-yl furan-2-carboxylate, 4r

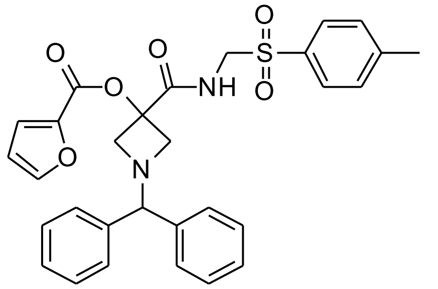

0.444 g (82%) white solid.
M.p.: 106.5 °C
^{1}H NMR (500 MHz, $CDCl_3$) δ 8.50 (t, J = 6.1 Hz, 1H), 7.79 (d, J = 8.1 Hz, 2H), 7.57 (s, 1H), 7.48 (d, J = 7.4 Hz, 4H), 7.37 (t, J = 7.5 Hz, 4H), 7.27 (dd, J = 21.1, 7.7 Hz, 4H), 7.19 (d, J = 3.3 Hz, 1H), 6.53 – 6.47 (m, 1H), 4.85 (d, J = 6.4 Hz, 2H), 4.48 (s, 1H), 3.67 (d, J = 9.2 Hz, 2H), 3.31 (d, J = 9.1 Hz, 2H), 2.41 (s, 3H)
^{13}C NMR (126 MHz, $CDCl_3$) δ 168.6, 156.6, 146.9, 145.5, 143.4, 140.3, 133.8, 130.0, 128.8, 128.6, 127.8, 127.4, 119.4, 112.0, 77.6, 72.9, 62.5, 60.1, 21.7.
HR-MS (ESI): *m/z* $[M+H]^+$ calcd for $C_{30}H_{29}N_2O_6S$: 545.1746; found: 545.1752.

1-benzhydryl-3-((tosylmethyl)carbamoyl)azetidin-3-yl acetate, 4s

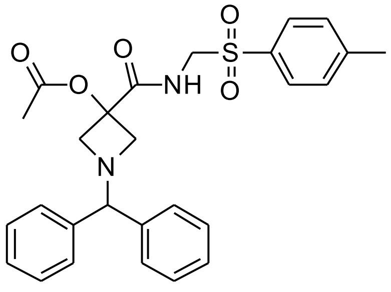

3.0 mmol scale 0.510 g (35%) yellowish white solid.
M.p.: 180.8 °C.
^{1}H NMR (500 MHz, $CDCl_3$) δ 8.51 (t, *J* = 6.3 Hz, 1H), 7.78 (d, *J* = 8.2 Hz, 2H), 7.45 (d, *J* = 7.2 Hz, 4H), 7.35 (t, *J* = 7.6 Hz, 5H), 7.29-7.21 (m, 3H), 4.83 (d, *J* = 6.5 Hz, 2H), 4.43 (s, 1H), 3.56 (dd, *J* = 7.1, 2.2 Hz, 2H), 3.15 (dd, *J* = 7.2, 2.1 Hz, 2H), 2.39 (s, 3H), 2.04 (s, 3H).
^{13}C NMR (126 MHz, $CDCl_3$) δ 169.3, 169.0, 145.5, 140.4, 133.9, 130.0, 128.8, 128.6, 127.8, 127.4, 77.7, 72.4, 62.6, 60.1, 21.7, 20.5.
HR-MS (ESI): *m/z* $[M+H]^+$ calcd for $C_{27}H_{29}N_2O_5S$: 493.1797; found: 493.1795.

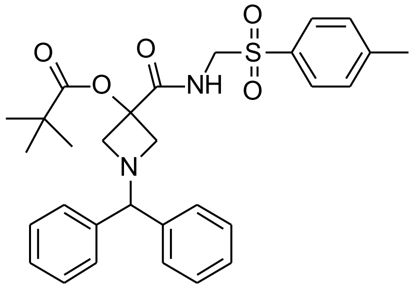

1-benzhydryl-3-((tosylmethyl)carbamoyl)azetidin-3-yl pivalate, 4t
5 days reaction time. 0.451g (56%) white solid.
M.p.: 154.4°C.
^{1}H NMR (500 MHz, $CDCl_3$) δ 8.46 (t, *J* = 6.2 Hz, 1H), 7.78 (d, *J* = 8.1 Hz, 2H), 7.46 (d, *J* = 7.5 Hz, 4H), 7.34 (t, *J* = 7.5 Hz, 4H), 7.28 – 7.20 (m, *J* = 17.4, 7.7 Hz, 4H), 4.81 (d, *J* = 6.4 Hz, 2H), 4.43 (s, 1H), 3.60 (d, *J* = 9.1 Hz, 2H), 3.11 (d, *J* = 9.0 Hz, 2H), 2.38 (s, 3H), 1.17 (s, 9H).
^{13}C NMR (126 MHz, $CDCl_3$) δ 177.0, 169.0, 145.4, 140.4, 133.8, 130.0, 128.8, 128.6, 127.8, 127.4, 77.8, 72.0, 62.9, 60.1, 38.4, 26.9, 21.7.
HR-MS (ESI): *m/z* $[M+H]^+$ calcd for $C_{30}H_{35}N_2O_5S$: 535.2267; found: 535.2272.

1-benzhydryl-3-((tosylmethyl)carbamoyl)azetidin-3-yl 2-chloroacetate, 4u

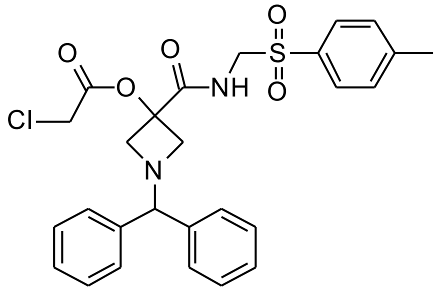

0.310 g (59%) yellowish white solid.
M.p.: decomposition above 120 °C.
^{1}H NMR (500 MHz, $CDCl_3$) δ 8.56 (t, *J* = 5.9 Hz, 1H), 7.77 (d, *J* = 8.1 Hz, 2H), 7.45 (d, *J* = 7.4 Hz, 4H), 7.35 (t, *J* = 7.5 Hz, 4H), 7.25 (t, *J* = 7.5 Hz, 4H), 4.82 (d, *J* = 6.5 Hz, 2H), 4.50 (s, 1H), 4.06 (s, 2H), 3.62 (d, *J* = 9.3 Hz, 2H), 3.28 (d, *J* = 8.2 Hz, 2H), 2.39 (s, 3H)
^{13}C NMR (126 MHz, $CDCl_3$) δ 168.3, 165.8, 145.6, 139.6, 133.7, 130.1, 128.9, 128.6, 128.0, 127.4, 77.3, 73.5, 62.0, 60.1, 40.3, 21.7
HR-MS (ESI): *m/z* $[M+H]^+$ calcd for $C_{27}H_{28}ClN_2O_5S$: 527.1407; found: 527.1407.

1-benzhydryl-3-((tosylmethyl)carbamoyl)azetidin-3-yl methyl malonate, 4v

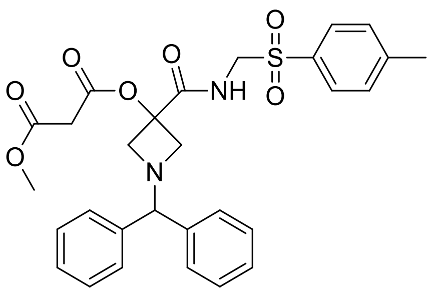

4 days reaction time. 0.472 g (43%) white solid.
M.p.: 114.3 °C.
H NMR (500 MHz, $CDCl_3$) δ 8.46 (t, *J* = 6.5 Hz, 1H), 7.80 (d, *J* = 8.2 Hz, 2H), 7.45 (d, *J* = 7.3 Hz, 4H), 7.33 (t, *J* = 7.6 Hz, 4H), 7.29 – 7.21 (m, 4H), 4.81 (d, *J* = 6.6 Hz, 2H), 4.54 (s, 1H), 3.76 (s, 3H), 3.57 (d, *J* = 9.8 Hz, 2H), 3.49 (s, 2H), 3.37 (d, *J* = 9.4 Hz, 2H), 2.37 (s, 3H)
^{13}C NMR (126 MHz, $CDCl_3$) δ 168.5, 167.4, 164.7, 145.3, 140.5, 133.9, 129.9, 128.8, 128.7, 127.7, 127.4, 77.2, 74.3, 61.6, 60.1, 52.9, 40.8, 21.6.
HR-MS (ESI): *m/z* $[M+H]^+$ calcd for $C_{29}H_{31}N_2O_7S$: 551.1852; found: 551.1850.

1-benzhydryl-3-(4-nitrophenoxy)-N-(tosylmethyl)azetidine-3-carboxamide, 5a

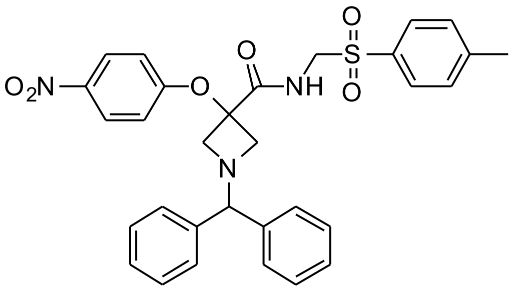

0.170 g (30%) yellowish solid.
M.p.: 177.3 °C.
^{1}H NMR (500 MHz, $CDCl_3$) δ 8.05 (d, *J* = 9.2 Hz, 2H), 7.93 (t, *J* = 6.5 Hz, 1H), 7.72 (d, *J* = 8.2 Hz, 2H), 7.43 (d, *J* = 7.3 Hz, 4H), 7.33 (t, *J* = 7.5 Hz, 4H), 7.30-7.22 (m, 4H), 6.50 (d, *J* = 9.2 Hz, 2H), 4.79 (d, *J* = 6.6 Hz, 2H), 4.46 (s, 1H), 3.64 (d, *J* = 9.3 Hz, 2H), 3.32 (d, *J* = 9.3 Hz, 2H), 2.45 (s, 3H).
^{13}C NMR (126 MHz, $CDCl_3$) δ 169.4, 159.7, 145.7, 142.3, 140.5, 133.8, 130.0, 128.8, 128.6, 127.8, 127.3, 125.7, 115.7, 75.1, 61.4, 59.7, 21.7
HR-MS (ESI): *m/z* $[M+H]^+$ calcd for $C_{31}H_{30}N_3O_6S$: 572.1855; found: 572.1849.

tert-butyl 3-(2,4-dinitrophenoxy)-3-((tosylmethyl)carbamoyl)azetidine-1-carboxylate, 5b

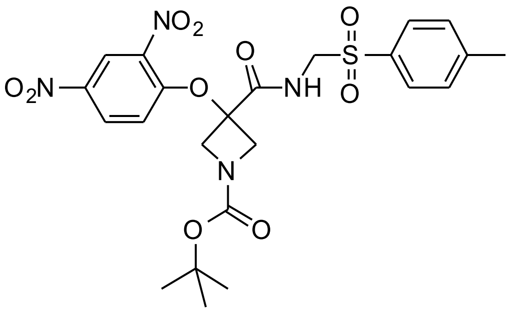

0.191 g (35%) yellow solid.
M.p.: 100.1 °C
^{1}H NMR (500 MHz, $CDCl_3$) δ 8.72 (d, *J* = 2.4 Hz, 1H), 8.30 (dd, *J* = 9.2, 2.4 Hz, 1H), 7.63 (t, *J* = 6.6 Hz, 1H), 7.56 (d, *J* = 8.0 Hz, 2H), 7.28 (d, *J* = 7.9 Hz, 2H), 6.61 (d, *J* = 9.2 Hz, 1H), 4.64 (d, *J* = 6.6 Hz, 2H), 4.36 (d, *J* = 9.6 Hz, 2H), 4.10 (d, *J* = 9.6 Hz, 2H), 2.41 (s, 3H), 1.39 (s, 9H)
^{13}C NMR (126 MHz, $CDCl_3$) δ 167.7, 155.5, 151.8, 146.0, 141.8, 139.7, 133.7, 130.1, 129.2, 128.4, 122.4, 116.1, 81.0, 77.8, 60.1, 28.2, 21.7
HR-MS (ESI): *m/z* $[M+Na]^+$ calcd for $C_{23}H_{26}N_4O_{10}SNa$: 573.1267; found: 573.1274.

General procedure for the Ugi reaction

The heterocyclic ketone (1.0 mmol), acid component (2.0 mmol), amine component (2.0 mmol) and the isocyanide component (2.0 mmol) were dissolved in 1.67 ml of trifluorethanol, and stirred at 40 °C for two days. The mixture was concentrated under reduced pressure and subjected to column chromatography. In the case of the aggressive smelling isocyanides, after two days 5 ml of 1M HCl was added and the two phase system was vigorously stirred for 30 min, then the phases were separated, the organic phase was concentrated under reduced pressure and subjected to column chromatography.

1-benzhydryl-3-(N-butylbenzamido)-N-(tosylmethyl)azetidine-3-carboxamide, 6a

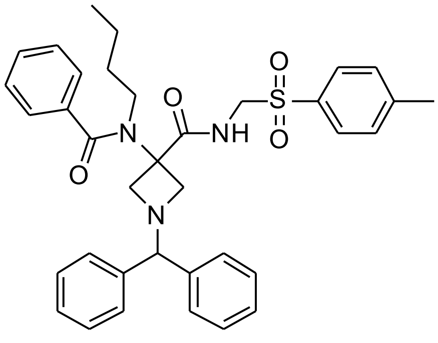

Following the general procedure 0.365 g (60%) tan solid was obtained.
M.p.: 131.2 °C
^{1}H NMR (300 MHz, $CDCl_3$) δ 9.09 (t, *J* = 5.8 Hz, 1H), 7.84 (d, *J* = 8.2 Hz, 2H), 7.50 – 7.20 (m, 17H), 4.87 (d, *J* = 5.9 Hz, 2H), 4.40 (s, 1H), 3.79 (bs, 2H), 3.15 (d, *J* = 7.5 Hz, 2H), 3.04 – 2.91 (m, 2H), 2.38 (s, 3H), 1.25-1.11 (m, 2H), 0.99 – 0.81 (m, 2H), 0.61 (t, *J* = 7.2 Hz, 3H).
^{13}C NMR (75 MHz, $CDCl_3$) δ 171.7, 161.7, 145.4, 140.9, 134.8, 130.2, 130.1, 129.0, 129.0, 128.6, 127.9, 127.7, 127.0, 78.2, 60.8, 60.1, 48.1, 31.7, 21.9, 20.3, 20.2, 13.5.
HR-MS (ESI): *m/z* $[M+H]^+$ calcd for $C_{36}H_{40}N_3O_4S$: 610.2740; found: 610.2748.

tert-butyl 3-(N-butylbenzamido)-3-((tosylmethyl)carbamoyl)azetidine-1-carboxylate, 6b

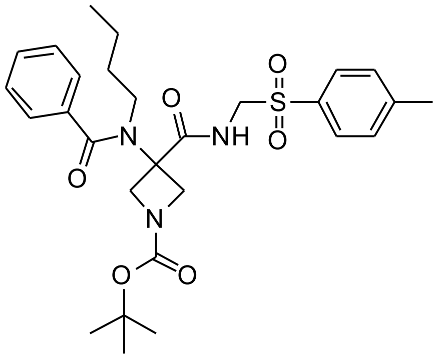

Following the general procedure 0.433 g (80%) colourless amorphous solid was obtained.
M.p.: -
^{1}H NMR (500 MHz, $CDCl_3$) δ 8.48 (s, 1H), 7.83 (d, *J* = 8.1 Hz, 2H), 7.46 (dt, *J* = 13.9, 7.1 Hz, 5H), 7.34 (d, *J* = 7.9 Hz, 2H), 4.73 (d, *J* = 6.1 Hz, 2H), 4.34 (d, *J* = 9.2 Hz, 2H), 4.05 (d, *J* = 9.4 Hz, 2H), 3.28 – 3.18 (m, 2H), 2.43 (s, 3H), 1.46 (s, 9H), 1.28 – 1.22 (m, 2H), 1.02-0.94.98 (m, 2H), 0.66 (t, *J* = 7.3 Hz, 3H).
^{13}C NMR (126 MHz, $CDCl_3$) δ 174.0, 171.5, 156.1, 145.4, 134.9, 134.5, 130.8, 130.0, 128.7, 128.6, 127.4, 80.3, 60.8, 59.6, 48.4, 30.9, 28.3, 28.3, 21.7, 19.8, 13.1.
HR-MS (ESI): *m/z* $[M+H]^+$ calcd for $C_{28}H_{38}N_3O_6S$: 544.2481; found: 544.2488.

3-(N-benzyl-2-chlorobenzamido)-N-(tosylmethyl)oxetane-3-carboxamide, 6c

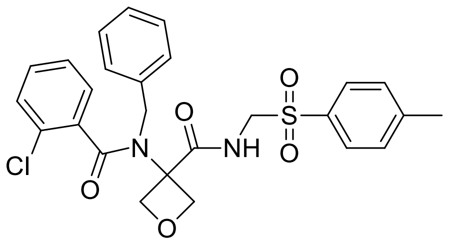

This substance was synthesized using modified general procedure. Oxetan-3-one (108 mg, 1.5 mmol, 1 equiv), 2-chlorobenzoic acid (469 mg, 3.0 mmol, 2.0 equiv), benzylamine (321 mg, 328 µl, 3.0 mmol, 2.0 equiv), TosMIC (585 mg, 3.0 mmol, 2.0 equiv) and 2,2,2-trifluoroethanol (6 ml) was stirred together for two days at 40 °C. Then the mixture was concentrated under reduced pressure, dissolved in 20 ml of EtOAc and washed with water, sat. $NaHCO_3$ solution and brine, concentrated again and subjected to column chromatography. 0.523 g (68%) white solid was obtained.
M.p.: 71.2 °C
^{1}H NMR (500 MHz, $CDCl_3$) δ7.84 (d, *J* = 8.2 Hz, 2H), 7.80 (t, *J* = 6.5 Hz, 1H), 7.43 – 7.31 (m, 5H), 7.29 – 7.21 (m, 5H), 7.07 – 7.02 (m, 2H), 4.86 (d, *J* = 7.2 Hz, 2H), 4.71 (d, *J* = 7.4 Hz, 2H), 4.65 (d, *J* = 5.8 Hz, 2H), 4.34 (s, 2H), 2.43 (s, 3H).
^{13}C NMR (126 MHz, $CDCl_3$) δ 170.7, 170.2, 145.6, 135.1, 134.5, 134.4, 131.2, 130.6, 130.2, 130.0, 129.01, 128.96, 128.6, 128.5, 127.7, 127.4, 77.4, 65.0, 60.8, 51.4, 21.9.
HR-MS (ESI): *m/z* $[M+H]^+$ calcd for $C_{23}H_{29}N_2O_5S$: 445.1797; found: 445.1804.

ethyl 2-(1-benzhydryl-3-(N-butylbenzamido)azetidine-3-carboxamido)acetate, 6d

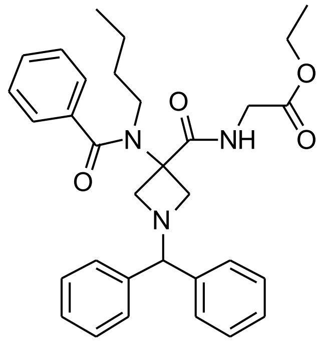

Following the general procedure 0.430 g (81%) white solid was obtained.
M.p.: 158.5 °C
^{1}H NMR (500 MHz, $CDCl_3$) δ 8.65 (s, 1H), 7.49 – 7.42 (m, *J* = 11.7, 5.7 Hz, 6H), 7.41 – 7.36 (m, 3H), 7.29 (t, *J* = 7.6 Hz, 4H), 7.20 (t, *J* = 7.3 Hz, 2H), 4.40 (s, 1H), 4.28 (q, *J* = 7.1 Hz, 2H), 4.19 (d, *J* = 5.3 Hz, 2H), 3.97 (bs, 2H), 3.20 (d, *J* = 7.1 Hz, 2H), 3.14 (t, *J* = 8.1 Hz, 2H), 1.42 – 1.30 (m, *J* = 14.3, 11.8 Hz, 5H), 0.97 (d, *J* = 6.4 Hz, 2H), 0.64 (t, *J* = 6.9 Hz, 3H).
^{13}C NMR (126 MHz, $CDCl_3$) δ 172.3, 169.9, 141.2, 136.4, 129.6, 128.6, 128.3, 127.39, 127.38, 126.7, 78.1, 77.2, 61.3, 59.9, 47.9, 41.8 31.5, 19.9, 14.2, 13.3.
HR-MS (ESI): *m/z* $[M+H]^+$ calcd for $C_{32}H_{38}N_3O_4$: 528.2862; found: 528.2852.

1-benzhydryl-3-(N-butyl-2-chlorobenzamido)-N-(tosylmethyl)azetidine-3-carboxamide, 6e

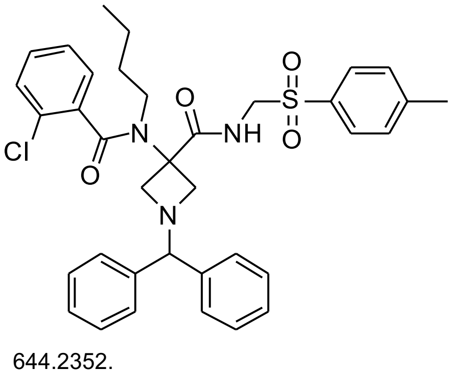

Following the general procedure 0.420g (65%) white solid was obtained.
M.p.: 164.0 °C
^{1}H NMR (500 MHz, $CDCl_3$) δ 9.03 (s, 1H), 7.85 (d, *J* = 8.2 Hz, 2H), 7.54-7.41 (m, 4H), 7.41 – 7.21 (m, 12H), 4.87 (dd, *J* = 27.1, 20.3 Hz, 2H), 4.40 (s, 1H), 3.80 (d, *J* = 108.5 Hz, 2H), 3.19 (dd, *J* = 30.8, 23.9 Hz, 2H), 2.92 (d, *J* = 34.8 Hz, 2H), 2.38 (s, 3H), 1.13 (s, 2H), 0.89 (dd, *J* = 13.9, 6.9 Hz, 2H), 0.57 (t, *J* = 7.3 Hz, 3H).
^{13}C NMR (126 MHz, $CDCl_3$) δ 171.1, 169.2, 145.1, 140.6, 135.5, 134.6, 130.4, 130.1, 129.9, 129.5, 128.8, 128.7, 128.3, 127.6, 127.4, 127.0, 77.8, 60.6, 59.5, 46.8, 30.9, 21.6, 19.8, 13.1.
HR-MS (ESI): *m/z* $[M+H]^+$ calcd for $C_{36}H_{39}ClN_3O_4S$: 644.2350; found: 644.2352.

1-benzhydryl-3-(N-butylcinnamamido)-N-(tosylmethyl)azetidine-3-carboxamide, 6f

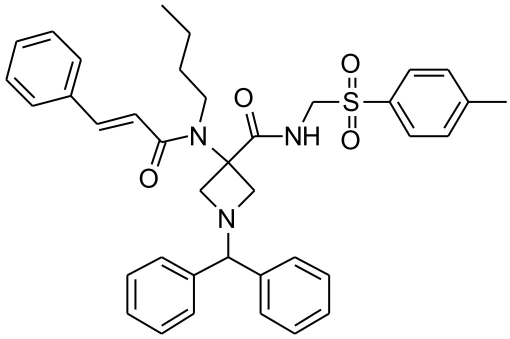

Following the general procedure 0.250 g (40%) white amorphous solid was obtained.
M.p.: -
^{1}H NMR (500 MHz, $CDCl_3$) δ 8.87 (t, *J* = 6.3 Hz, 1H), 7.81 (d, *J* = 8.3 Hz, 2H), 7.66 (d, *J* = 15.3 Hz, 1H), 7.48 (d, *J* = 5.2 Hz, 2H), 7.44 (d, *J* = 7.4 Hz, 4H), 7.40 – 7.35 (m, 3H), 7.33 (t, *J* = 7.6 Hz, 4H), 7.29 – 7.21 (m, 4H), 6.72 (d, *J* = 15.4 Hz, 1H), 4.83 (d, *J* = 5.5 Hz, 2H), 4.36 (s, 1H), 3.76 (d, *J* = 7.0 Hz, 2H), 3.11 (d, *J* = 7.2 Hz, 2H), 3.08 – 3.00 (m, 2H), 2.39 (s, 3H), 1.59 – 1.47 (m, 2H), 1.25 – 1.20 (m, 2H), 0.91 (t, *J* = 7.4 Hz, 3H).
^{13}C NMR (126 MHz, $CDCl_3$) δ 171.4, 167.5, 145.1, 144.0, 140.7, 134.9, 134.3, 130.0, 129.9, 128.9, 128.8, 128.7, 127.9, 127.6, 127.4, 116.7, 77.9, 77.3, 77.0, 60.5, 60.4, 46.2, 33.1, 21.7, 20.3, 13.6.
HR-MS (ESI): *m/z* $[M+H]^+$ calcd for $C_{38}H_{42}N_3O_4S$: 636.2896; found: 636.2889.

1-benzhydryl-3-(N-benzylbenzamido)-N-(tosylmethyl)azetidine-3-carboxamide, 6g

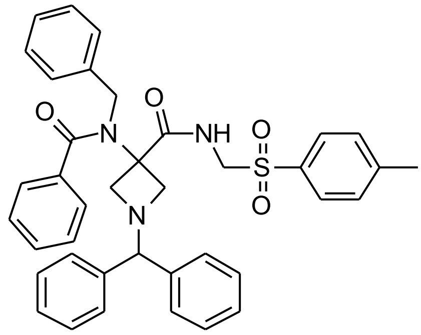

Following the general procedure 0.483 g (50%) white solid was obtained.
M.p.: 92.2°C
^{1}H NMR (500 MHz, $CDCl_3$) δ 9.04 (bs, 1H), 7.83 (d, *J* = 8.2 Hz, 2H), 7.39 – 7.20 (m, 20H), 7.11 (d, *J* = 7.2 Hz, 2H), 4.84 (bs, 2H), 4.32 (s, 1H), 4.19 (bs, 2H), 3.60 (bs, 2H), 3.23 (bs, 2H), 2.40 (s, 3H).
^{13}C NMR (126 MHz, $CDCl_3$) δ 174.1, 171.0, 145.1, 140.4, 137.8, 135.4, 134.3, 130.0, 129.9, 128.8, 128.7, 128.6, 128.4, 128.2, 127.6, 127.4, 126.63, 126.56, 77.4, 61.4, 61.0, 60.5, 52.0, 21.7.
HR-MS (ESI): *m/z* $[M+H]^+$ calcd for $C_{39}H_{38}N_3O_4S$: 644.2583; found: 644.2578.

Tert-butyl 3-(N-benzyl-2-chlorobenzamido)-3-((tosylmethyl)carbamoyl)azetidine-1-carboxylate, 6h

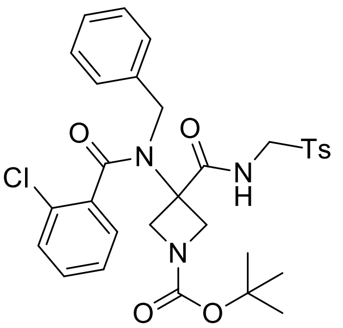

Following the general procedure 0.555 g (91%) colorless gummy oil was obtained.

M.p.: -

^{1}H NMR (500 MHz, $CDCl_3$, 45°C) δ 7.82 (d, *J* = 8.1 Hz, 2H), 7.76 (t, *J* = 5.8 Hz, 1H), 7.39-7.30 (m, 5H), 7.25-7.23 (m, 4H), 7.02-7.02 (m, 2H), 4.59 (d, *J* = 5.8 Hz, 2H), 4.41 (s, 2H), 4.19 (bs, 4H), 2.42 (s, 3H), 1.45 (s, 9H)

^{13}C NMR (126 MHz, $CDCl_3$, 45°C) δ 170.8, 170.1, 156.1, 145.5, 135.3, 134.6, 134.5, 131.2, 130.6, 130.1, 129.9, 129.0, 128.9, 128.7, 128.4, 127.4, 80.4, 60.9, 60.5, 56.0, 51.5, 28.5, 21.8.

HR-MS (ESI): *m/z* $[M+H]^+$ calcd $C_{31}H_{34}ClN_3O_6S$: 612.1930; found: 612.1920.

Tert-butyl 3-(N-(3,5-bis(trifluoromethyl)benzyl)-2-chlorobenzamido)-3-((tosylmethyl)carbamoyl)azetidine-1-carboxylate, 6i

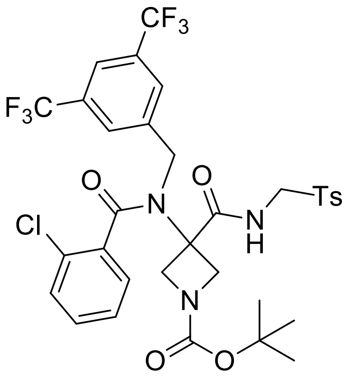

Following the general procedure 0.685 g(92%). white solid was obtained.

M.p.: 98.1 °C;

^{1}H NMR (500 MHz, $CDCl_3$, 45°C) δ 7.90 (t, *J* = 6.2 Hz, 1H), 7.81 (d, *J* = 8.1 Hz, 2H), 7.69 (s, 1H), 7.39 (s, 2H), 7.34 (d, *J* = 8.1 Hz, 2H), 7.26 (s, 2H), 7.18 (s, 2H), 4.69 (s, 2H), 4.47 (s, 2H), 4.28 (d, *J* = 9.5 Hz, 2H), 4.15 (d, *J* = 9.5 Hz, 2H), 2.40 (s, 3H), 1.46 (s, 9H)

^{13}C NMR (126 MHz, $CDCl_3$, 45°C) δ 170.7, 170.0, 156.2, 145.6, 138.5, 134.5, 134.1, 132.1 (q, *J* = 34 Hz), 131.5, 130.5, 130.1, 130.0, 128.8, 128.1, 127.4, 127.3, 123.0 (q, *J* = 273 Hz), 122.0, 80.9, 60.8, 60.7, 56.0, 50.7, 28.3, 21.6

HR-MS (ESI): *m/z* $[M+H]^+$ calcd $C_{33}H_{32}ClF_6N_3O_6S$: 748.1677; found: 748.1658.

Tert-butyl 3-(2-chloro-N-(thiophen-2-ylmethyl)benzamido)-3-((tosylmethyl)carbamoyl)azetidine-1-carboxylate, 6j

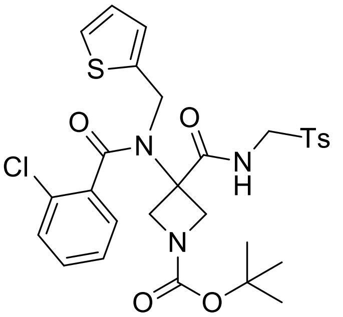

Following the general procedure 0.596 g (96%) colorless gummy oil was obtained.

M.p.: -

^{1}H NMR (500 MHz, $CDCl_3$, 45°C) δ 7.82 (d, *J* = 8.2 Hz, 2H), 7.57 (t, *J* = 6.2 Hz, 1H), 7.43-7.31 (m, 6H), 7.20 (d, *J* = 5.2 Hz, 1H), 6.85 (t, *J* = 5.2 Hz, 1H), 6.68 (s, 1H), 4.59-4.57 (m, 4H), 4.24-4.19 (m, 4H), 2.43 (s, 3H), 1.46 (s, 9H).

^{13}C NMR (126 MHz, $CDCl_3$, 45°C) δ 170.3, 170.0, 156.2, 145.5, 138.1, 134.4, 134.4, 131.4, 130.7, 130.1, 130.0, 129.0, 128.8, 127.4, 127.3, 126.4, 80.5, 60.9, 60.2, 56.0, 46.2, 28.5, 21.8.

HR-MS (ESI): *m/z* $[M+H]^+$ calcd $C_{29}H_{32}ClN_3O_6S_2$: 618.1494; found: 618.1485.

Tert-butyl 3-(N-benzyl-2-chlorobenzamido)-3-(tert-butylcarbamoyl)azetidine-1-carboxylate, 6k

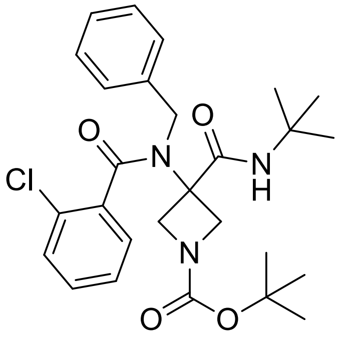

Following the general procedure 0.460 g (92%) colorless gummy oil was obtained.

M.p.: -

^{1}H NMR (500 MHz, $CDCl_3$, 45°C) δ 7.35 (d, *J* = 8.1 Hz, 1H), 7.28 (d, *J* = 8.1 Hz, 1H), 7.19-7.12 (m, 6H), 7.00 (bs, 2H), 4.44-4.41 (m, 4H), 4.21 (bs, 2H), 1.43 (s, 9H), 1.32 (s, 9H).

^{13}C NMR (126 MHz, $CDCl_3$, 45°C) δ 170.4, 169.2, 156.4, 135.8, 135.1, 130.9, 130.7, 129.9, 128.7, 128.3, 128.0, 127.4, 127.1, 80.1, 61.4, 56.0, 51.4, 51.4, 28.7, 28.4.

HR-MS (ESI): *m/z* $[M+H]^+$ calcd $C_{27}H_{34}ClN_3O_4$: 500.2311; found: 500.2308.

Tert-butyl 3-(N-(3,5-bis(trifluoromethyl)benzyl)-2-chlorobenzamido)-3-(tert-butylcarbamoyl)azetidine-1-carboxylate, 6l

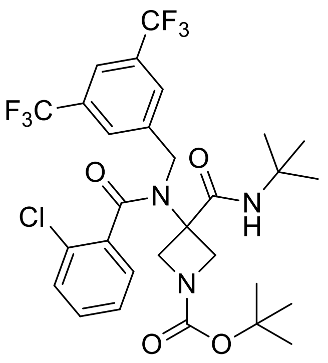

Following the general procedure 0.610 g (96%) white solid was obtained.
M.p.: 153.7 °C
^{1}H NMR (500 MHz, $CDCl_3$, 45°C) δ 7.69 (s, 1H), 7.39 (s, 2H), 7.32-7.26 (m, 2H), 7.16 (d, *J* = 7.2 Hz, 1H), 7.05-7.04 (m, 2H), 4.51 (s, 2H), 4.46 (d, *J* = 9.5 Hz, 2H), 4.17 (bs, 2H), 1.44 (s, 9H), 1.34 (s, 9H).
^{13}C NMR (126 MHz, $CDCl_3$, 45°C) δ 170.3, 169.0, 156.4, 138.9, 134.7, 132.2 (q, *J* = 33 Hz), 131.5, 130.7, 130.2, 128.1, 127.9, 127.2, 123.1 (q, *J* = 273 Hz), 122.0, 80.6, 61.8, 56.1, 51.7, 50.7, 28.7, 24.4.
HR-MS (ESI): *m/z* $[M+H]^+$ calcd $C_{29}H_{32}ClF_6N_3O_4$: 636.2058; found: 636.2045.

Tert-butyl 3-(tert-butylcarbamoyl)-3-(2-chloro-N-(thiophen-2-ylmethyl)benzamido)azetidine-1-carboxylate, 6m

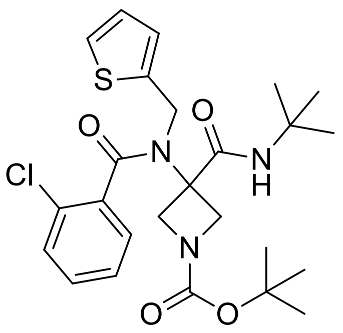

Following the general procedure 0.477 g (94%) colorless gummy oil was obtained.
M.p.: -
^{1}H NMR (600 MHz, DMSO-d_6, 50°C) δ 7.56-7.54 (m, 1H), 7.52-7.47 (m, 2H), 7.44-7-43 (m, 2H), 6.92-6.91 (m, 1H), 6.83 (s, 1H), 6.61 (s, 1H), 4.71 (bs, 2H), 4.23-4.19 (m, 4H), 1.40 (s, 9H), 1.22 (s, 9H).
^{13}C NMR (150 MHz, DMSO-d_6, 50°C) δ 168.4, 168.3, 155.3, 138.3, 134.3, 131.1, 129.5, 128.3, 127.2, 127.0, 126.9, 126.1, 78.9, 60.0, 55.8, 50.4, 45.2, 27.9, 27.8.
HR-MS (ESI): *m/z* $[M+H]^+$ calcd $C_{25}H_{32}ClN_3O_4S$: 506.1875; found: 506.1873.

Tert-butyl 3-(tert-butylcarbamoyl)-3-(2-chloro-N-cyclohexylbenzamido)azetidine-1-carboxylate, 6n

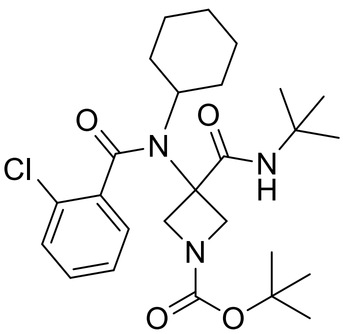

Following the general procedure 0.211 g (43%) white solid was obtained.
M.p.: 179.2 °C
^{1}H NMR (500 MHz, $CDCl_3$, 45°C) δ 7.41 (d, *J* = 7.5 Hz, 2H), 7.37-7.31 (m, 2H), 7.26 (d, *J* = 7.5 Hz, 1H), 4.61 (bs, 1H), 4.40 (d, *J* = 9.0 Hz, 1H), 4.19 (d, *J* = 9.0 Hz, 2H), 3.18 (t, *J* = 12.5 Hz, 1H), 1.88-1.86 (m, 1H), 1.77-1.70 (m, 2H), 1.62-1.57 (m, 1H), 1.48-1.42 (m, 12H),1.37 (s, 9H), 1.08-1.01 (m, 1H), 0.93-0.81 (m, 2H)
^{13}C NMR (126 MHz, $CDCl_3$, 45°C) δ 171.0, 170.5, 156.5, 136.6, 131.0, 130.7, 130.0, 127.8, 126.9, 80.1, 61.5, 61.0, 51.3, 33.3, 28.7, 28.5, 26.6, 26.5, 25.1
HR-MS (ESI): *m/z* $[M+H]^+$ calcd $C_{26}H_{38}ClN_3O_4$: 492.2624; found: 492.2618

Tert-butyl 3-(tert-butylcarbamoyl)-3-(2-chloro-N-(prop-2-yn-1-yl)benzamido)azetidine-1-carboxylate, 6o

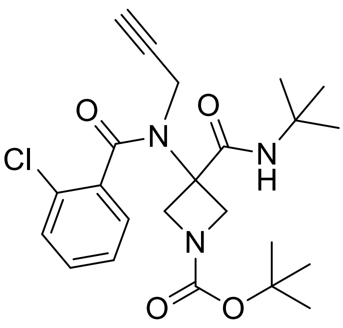

Following the general procedure 0.326 g (73%) colorless gummy oil was obtained.
M.p.: -
^{1}H NMR (500 MHz, $CDCl_3$, 45°C) δ 7.42-7.34 (m, 4H), 6.80 (s, 1H), 4.45 (d, *J* = 9.5 Hz, 2H), 4.26 (bs, 2H), 3.92 (s, 2H), 2.29 (t, *J* = 2.2 Hz, 1H), 1.43 (s, 9H), 1.35 (s, 9H).
^{13}C NMR (126 MHz, $CDCl_3$, 45°C) δ 169.5, 168.9, 156.3, 134.3, 131.3, 130.7, 130.0, 128.2, 127.4, 80.2, 77.8, 74.6, 60.7, 56.0, 51.6, 37.2, 28.7, 28.4.
HR-MS (ESI): *m/z* $[M+H]^+$ calcd $C_{23}H_{30}ClN_3O_4$: 448.1998; found: 448.1993.

Substitution of the tosyl group

tert-butyl 3-(N-benzyl-2-chlorobenzamido)-3-((ethoxymethyl)carbamoyl)azetidine-1-carboxylate, 7a

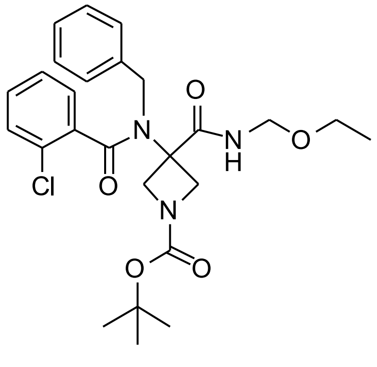

In a 4 ml vial, **6h** (160 mg, 0.26 mmol) was dissolved in 3 ml EtOH, NaOEt (126 mg, 0.39 mmol, 1.5 equivalent as a 21 w/w% solution in EtOH) was added and stirred at room temperature for an hour. Upon completion, it was diluted with EtOAc, extracted with sat. citric acid solution, then brine and dried with Na_2SO_4, concentrated under reduced pressure and finally subjected to column chromatography. Yield 120 mg (92%) as white amorphous solid.

M.p.: -

^{1}H NMR (500 MHz, $CDCl_3$, 45 °C) δ 7.70 (t, *J* = 6.7 Hz, 1H), 7.37 (d, *J* = 8.1 Hz, 1H), 7.33 – 7.27 (m, 1H), 7.25 – 7.14 (m, 5H), 7.07 – 6.98 (m, 2H), 4.73 (d, *J* = 6.5 Hz, 2H), 4.51 – 4.40 (m, 4H), 4.26 (d, *J* = 9.3 Hz, 2H), 3.53 (q, *J* = 7.0 Hz, 2H), 1.44 (s, 9H), 1.20 (t, *J* = 7.0 Hz, 3H).

^{13}C NMR (126 MHz, $CDCl_3$, 45 °C) δ 171.0, 170.6, 156.4, 135.8, 135.0, 131.0, 130.6, 129.9, 128.8, 128.5, 128.2, 127.3, 127.1, 80.4, 70.7, 64.3, 60.9, 56.1, 51.5, 28.4, 15.2.

HR-MS (ESI): *m/z* $[M+H]^+$ calcd $C_{26}H_{33}ClN_3O_5$: 502.2103; found: 502.2097.

tert-butyl 3-(N-benzyl-2-chlorobenzamido)-3-(((methylthio)methyl)carbamoyl)azetidine-1-carboxylate, 7b

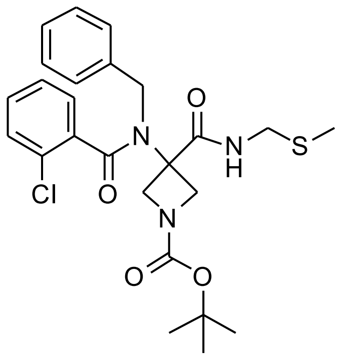

In a 4 ml vial, **6h** (162 mg, 0.264 mmol) was dissolved in 3 ml THF, NaSMe (132 mg, 0.396 mmol, 1.5 equivalent as a 21 w/w% aqeous solution) was added and stirred at room temperature for 1.5 hours. Upon completion, it was diluted with EtOAc, extracted with sat. citric acid solution, then brine and dried with Na_2SO_4, concentrated under reduced pressure and finally subjected to column chromatography. Yield 95 mg (71%) as white solid.

M.p.: 60.0 °C

^{1}H NMR (500 MHz, $CDCl_3$, 45 °C) δ 7.51 (t, *J* = 6.5 Hz, 1H), 7.38 (d, *J* = 8.0 Hz, 1H), 7.30 (t, *J* = 7.3 Hz, 1H), 7.25 – 7.15 (m, 5H), 7.06 – 7.00 (m, 2H), 4.50 – 4.41 (m, 4H), 4.34 (d, *J* = 4.5 Hz, 2H), 4.26 (d, *J* = 9.3 Hz, 2H), 2.17 (s, 3H), 1.45 (s, 9H).

^{13}C NMR (126 MHz, $CDCl_3$, 45 °C) δ 170.7, 170.4, 156.4, 135.7, 135.0, 131.1, 130.7, 130.0, 128.9, 128.5, 128.2, 127.3, 127.2, 80.4, 60.9, 56.1, 51.6, 43.7, 28.5, 14.9.

HR-MS (ESI): *m/z* $[M+H]^+$ calcd $C_{25}H_{31}ClN_3O_4S$: 504.1718; found: 504.1712.

tert-butyl 3-(N-benzyl-2-chlorobenzamido)-3-((morpholinomethyl)carbamoyl)azetidine-1-carboxylate, 7c

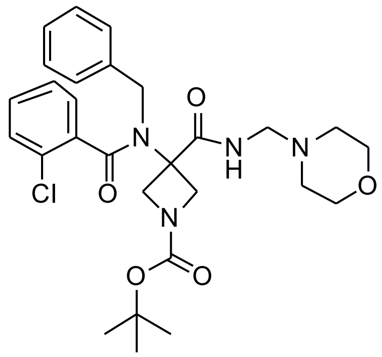

In a 4 ml vial, **6h** (110 mg, 0.18 mmol) was dissolved in 3 ml DCM (stabilised with amylene), morpholine (31 mg, 0.36 mmol, 31 μl, 2 equivalent) and TMG (41 mg, 0.36 mmol, 45 μl, 2 equivalent) were added and stirred at room temperature for 1 hour. Upon completion, it was diluted with EtOAc, extracted with sat. citric acid solution, sat $NaHCO_3$ solution then brine and dried with Na_2SO_4, concentrated under reduced pressure and finally subjected to column chromatography (DCM/MeOH gradient elution). Yield 61 mg (62%) as colourless waxy solid.

M.p.: -

^{1}H NMR (500 MHz, $CDCl_3$, 45 °C) δ 7.46 (t, *J* = 6.0 Hz, 1H), 7.37 (d, *J* = 8.1 Hz, 1H), 7.30 (dt, *J* = 8.2, 4.4 Hz, 1H), 7.25 – 7.16 (m, 5H), 7.06 – 6.97 (m, 2H), 4.51 – 4.38 (m, 4H), 4.26 (d, *J* = 9.1 Hz, 2H), 4.09 (d, *J* = 5.9 Hz, 2H), 3.73 – 3.66 (m, 4H), 2.56 – 2.50 (m, 4H), 1.44 (s, 9H).

^{13}C NMR (126 MHz, $CDCl_3$, 45 °C) δ 171.0, 170.5, 156.2, 135.6, 134.8, 130.9, 130.5, 129.8, 128.7, 128.3, 128.0, 127.2, 127.0, 80.2, 66.8, 62.0, 60.9, 56.1, 51.3, 50.3, 28.3.

HR-MS (ESI): *m/z* $[M+H]^+$ calcd $C_{28}H_{36}ClN_4O_5$: 543.2369; found: 543.2366.

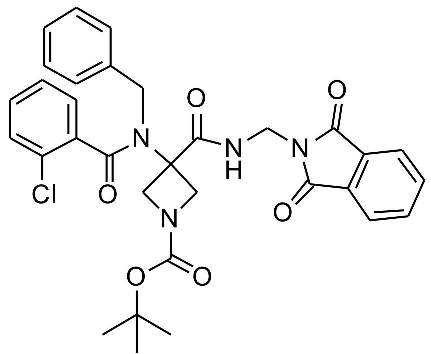

tert-butyl 3-(N-benzyl-2-chlorobenzamido)-3-(((1,3-dioxoisoindolin-2-yl)methyl)carbamoyl)azetidine-1-carboxylate, 7d

In a 4 ml vial, **6h** (110 mg, 0.18 mmol) was dissolved in 3 ml DMF, phthalimide-potassium (50 mg, 0.27 mmol, 1.5 equivalent) was added and stirred at room temperature for 5 hours. The reaction progress was

monitored with TLC. After 6 hours further 0.5 equivalent (17 mg. 0.9 mmol) phthalimide-potassium was added, and the reaction was stirred overnight. Then it was diluted with EtOAc, extracted with sat. citric acid solution, sat $NaHCO_3$ solution then brine and dried with Na_2SO_4, concentrated under reduced pressure and finally subjected to column chromatography. Yield 61 mg (56%) as colourless waxy solid.

M.p.: -

^{1}H NMR (500 MHz, $CDCl_3$) δ 7.96 (t, *J* = 5.8 Hz, 1H), 7.89 (dd, *J* = 5.4, 3.1 Hz, 2H), 7.76 (dd, *J* = 5.4, 3.0 Hz, 2H), 7.37 – 7.28 (m, 3H), 7.25 – 7.21 (m, 1H), 7.03 – 6.98 (m, 3H), 6.94 – 6.88 (m, 2H), 5.13 (d, *J* = 6.0 Hz, 2H), 4.54 – 4.33 (m, 4H), 4.22 (d, *J* = 6.0 Hz, 2H), 1.42 (s, 9H).

^{13}C NMR (126 MHz, $CDCl_3$) δ 170.5, 170.2, 167.2, 156.2, 135.2, 134.7, 134.4, 132.2, 131.1, 130.7, 129.9, 128.9, 128.7, 128.2, 127.6, 127.3, 123.8, 80.3, 60.5, 51.3, 42.9, 28.5.

HR-MS (ESI): *m/z* $[M+H]^+$ calcd $C_{32}H_{32}ClN_4O_6$: 603.2005; found: 603.1998.

dimethyl 2-((3-(N-benzyl-2-chlorobenzamido)-1-(tert-butoxycarbonyl)azetidine-3-carboxamido)methyl)malonate, 7e

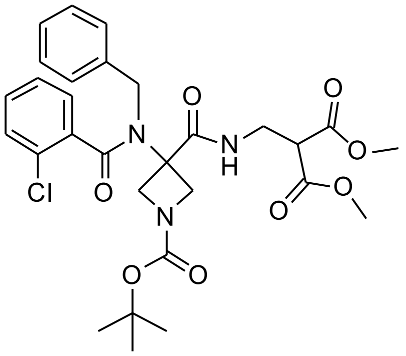

In a 4 ml vial, **6h** (122 mg, 0.2 mmol) was dissolved in 3 ml DCM (stabilised with amylene), dimethyl malonate (53 mg, 0.4 mmol, 46 µl, 2 equivalent) and Cs_2CO_3 (130 mg, 0.4 mmol, 2 equivalent) were added and stirred at room temperature for 2 hours. Upon completion, it was diluted with EtOAc, extracted with sat. citric acid solution, sat $NaHCO_3$ solution then brine and dried with Na_2SO_4, concentrated under reduced pressure and finally subjected to column chromatography. Yield 66 mg (56%) as white solid.

M.p.: 59.4 °C

^{1}H NMR (500 MHz, $CDCl_3$) δ 7.53 (t, *J* = 5.8 Hz, 1H), 7.37 (d, *J* = 7.9 Hz, 1H), 7.35 – 7.28 (m, 2H), 7.25 – 7.20 (m, 4H), 7.04 – 6.98 (m, 2H), 4.42 (s, 2H), 4.38 (d, *J* = 9.1 Hz, 2H), 4.22 (d, *J* = 9.5 Hz, 2H), 3.76 (s, 6H), 3.69 (dd, *J* = 6.3, 6.3 Hz, 2H), 3.62 (t, *J* = 6.6 Hz, 1H), 1.43 (s, 9H).

^{13}C NMR (126 MHz, $CDCl_3$) δ 170.6, 170.3, 168.3, 156.3, 135.4, 134.8, 131.0, 130.6, 129.8, 128.8, 128.6, 128.3, 127.4, 127.2, 80.2, 60.4, 52.9, 51.3, 51.0, 38.2, 28.4.

HR-MS (ESI): *m/z* $[M+H]^+$ calcd $C_{29}H_{35}ClN_3O_8$: 588.2107; found: 588.2101

tert-butyl 3-(N-benzyl-2-chlorobenzamido)-3-((cyanomethyl)carbamoyl)azetidine-1-carboxylate, 7f

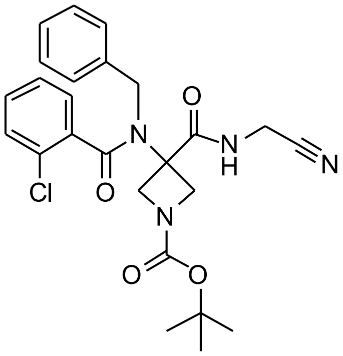

In a 4 ml vial, KCN (14 mg, 0.216 mmol, 1.2 equivalent) was suspended in 1.5 ml DMF, cooled to 0 °C and a solution of **6h** (110 mg, 0.18 mmol) in 1.5 ml DMF was slowly added. It was stirred at this temperature for 1 hour then allowed to warm to room temperature (roughly 0.5 hour). Then it was diluted with EtOAc, extracted with sat $NaHCO_3$ solution then brine and dried with Na_2SO_4, concentrated under reduced pressure and finally subjected to column chromatography. Yield 79 mg (92%) as white solid.

M.p.: 73.0 °C

^{1}H NMR (500 MHz, $CDCl_3$) δ 7.91 (t, *J* = 5.1 Hz, 1H), 7.44 (d, *J* = 8.0 Hz, 1H), 7.42 – 7.34 (m, 1H), 7.33 – 7.24 (m, 5H), 7.08 – 6.98 (m, 2H), 4.64 – 4.36 (m, 4H), 4.27 (d, *J* = 7.3 Hz, 2H), 4.10 (bs, 2H), 1.45 (s, 9H).

^{13}C NMR (126 MHz, $CDCl_3$) δ 171.1, 170.5, 156.2, 134.4, 134.3, 131.4, 130.7, 130.1, 129.1, 128.6, 128.6, 127.6, 127.4, 115.6, 80.5, 60.1, 55.4, 51.7, 28.4, 27.6.

HR-MS (ESI): *m/z* $[M+H]^+$ calcd $C_{25}H_{28}ClN_4O_4$: 483.1794; found: 483.1788.

tert-butyl 3-(allylcarbamoyl)-3-(N-benzyl-2-chlorobenzamido)azetidine-1-carboxylate, 7g

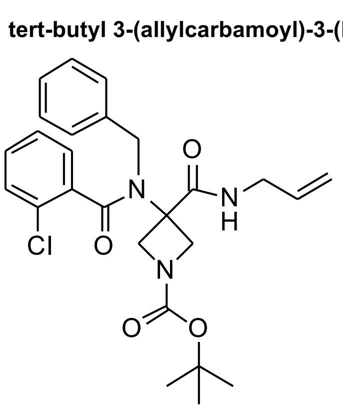

To 1 ml of dry THF at 0 °C, $ZnCl_2$ (45 mg, 0.33 mmol, 1.5 equivalent, 2 M in 2-MeTHF) and vinyl magnesium bromide (87 mg, 0.66 mmol, 3 equivalent, 1 M in THF) were added and stirred for 15 min. **6h** (135 mg, 0.22 mmol) was then added. It was stirred at this temperature for 1 hour then allowed to warm to room temperature (further 1 hour). It was quenched wit h sat NH_4Cl solution, diluted with EtOAc, and extracted with water, sat $NaHCO_3$ solution then brine and dried with Na_2SO_4, concentrated under reduced pressure and finally subjected to column chromatography. Yield 46 mg (43%) as yellowish waxy solid.

^{1}H NMR (500 MHz, $CDCl_3$, 45 °C) δ 7.36 (d, *J* = 8.1 Hz, 1H), 7.28 (td, *J* = 8.3, 7.6, 2.2 Hz, 1H), 7.24 – 7.12 (m, 6H), 7.03 – 6.97 (m, 2H), 5.82 (ddt, *J* = 16.2, 11.2, 5.7 Hz, 1H), 5.22 (dd, *J* = 17.2, 1.3 Hz, 1H), 5.15 (dd, *J* = 10.3, 1.2 Hz, 1H), 4.47 (d, *J* = 9.4

Hz, 2H), 4.42 (s, 2H), 4.25 (d, *J* = 7.6 Hz, 2H), 3.85 (t, *J* = 5.5 Hz, 2H), 1.44 (s, 9H).
M.p.: -
^{13}C NMR (126 MHz, $CDCl_3$, 45 °C) δ 170.7, 170.2, 156.4, 135.9, 135.0, 133.9, 131.0, 130.7, 130.0, 128.8, 128.5, 128.2, 127.3, 127.2, 116.9, 80.3, 60.9, 56.1, 51.5, 42.5, 28.5.
HR-MS (ESI): *m/z* $[M+H]^+$ calcd $C_{26}H_{31}ClN_3O_4$: 484.1998; found: 484.1993.

References

[1] K. Škoch, I. Císařová, P. Štěpnička, *Chem. Eur. J.* **2018**, *24*(52), 13788-13791.

[2] T. Matsuo, A. Hayashi, M. Abe, T. Matsuda, Y. Hisaeda, T. Hayashi, *J. Am. Chem. Soc.* **2009**, *131*(42), 15124-15125.

NMR spectra of the newly synthesized compounds

^{1}H NMR spectra of N-(3,5-dichlorobenzyl)formamide SI1

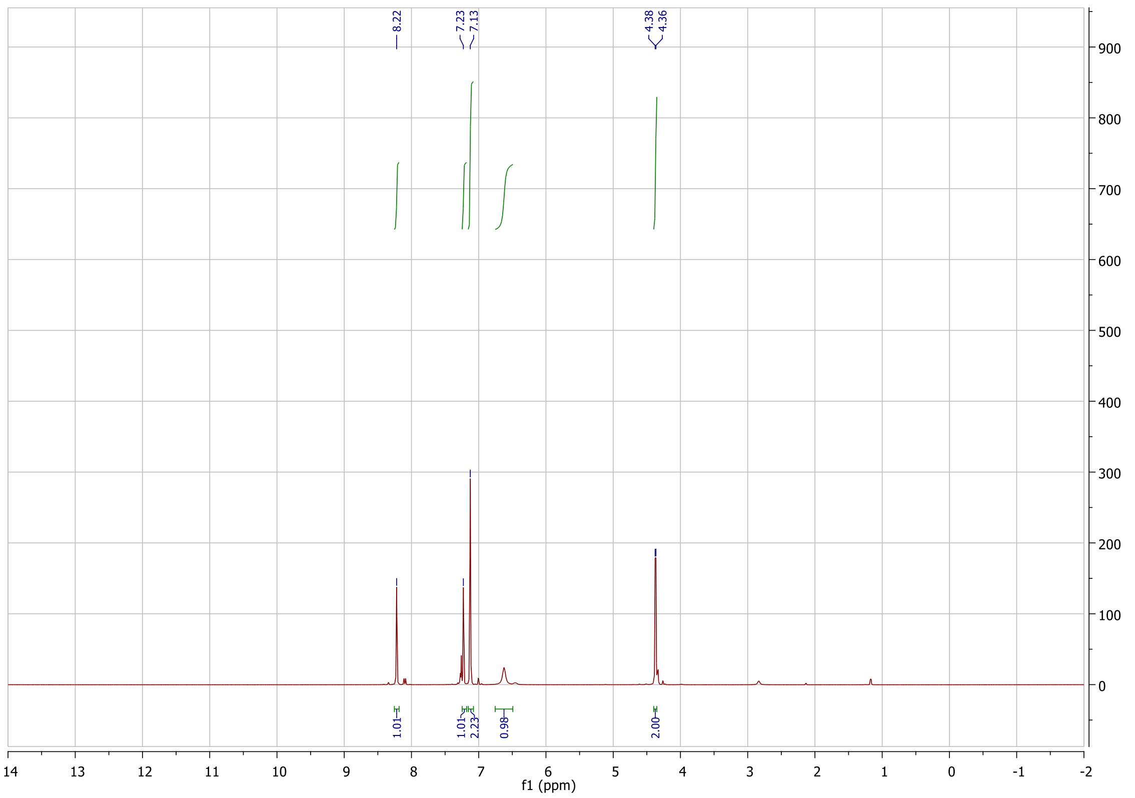

^{13}C NMR spectra of N-(3,5-dichlorobenzyl)formamide SI1

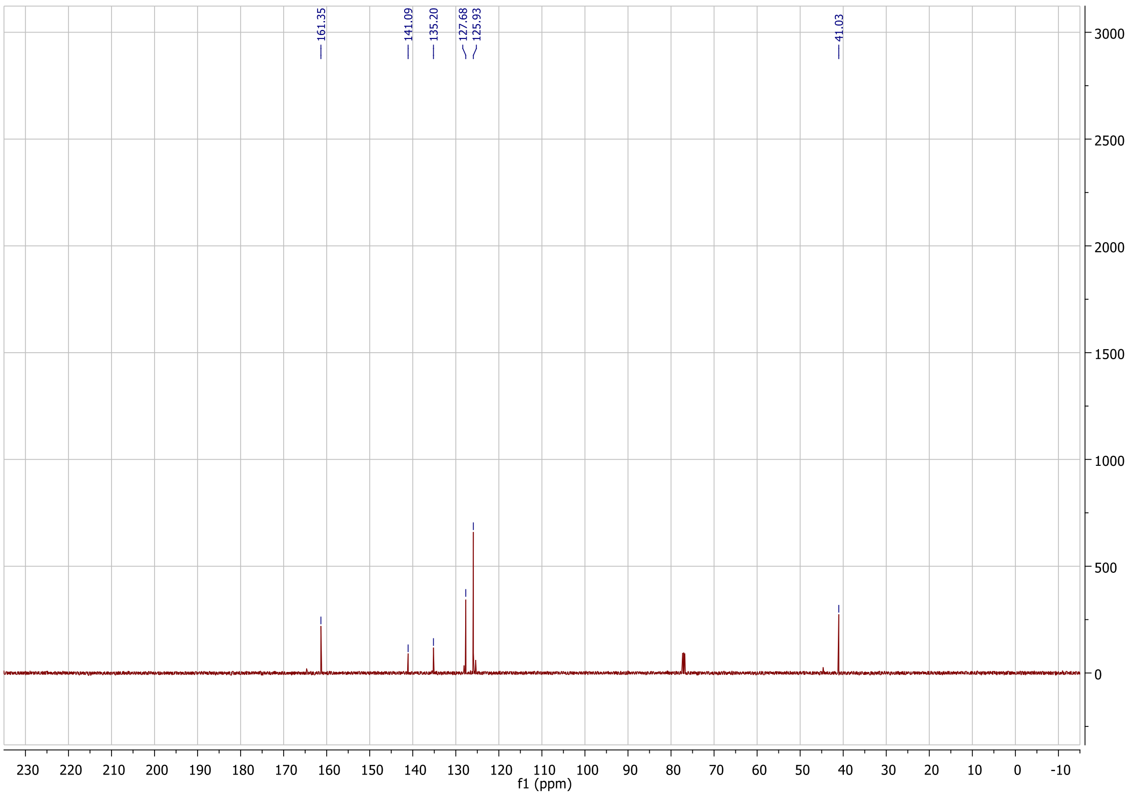

^1H NMR spectra of 1,3-dichloro-5-(isocyanomethyl)benzene SI2

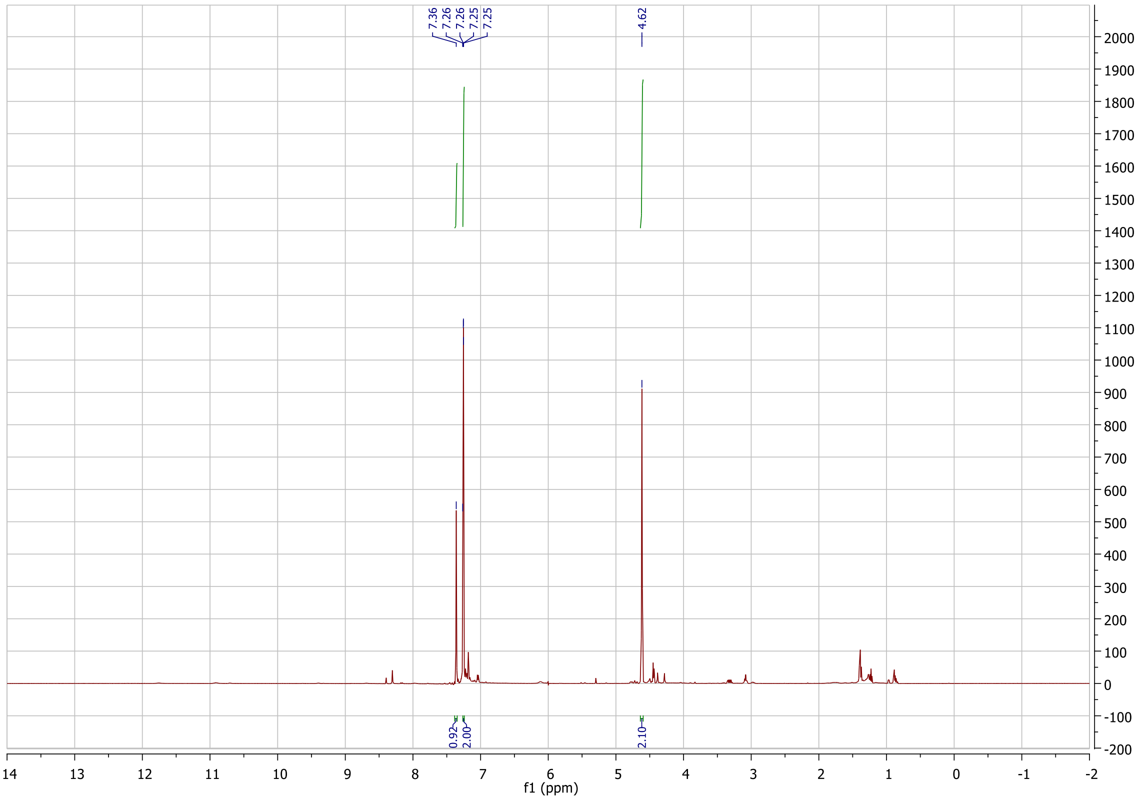

^{1}H NMR spectra of 1-benzhydryl-3-((tosylmethyl)carbamoyl)azetidin-3-yl benzoate 4a

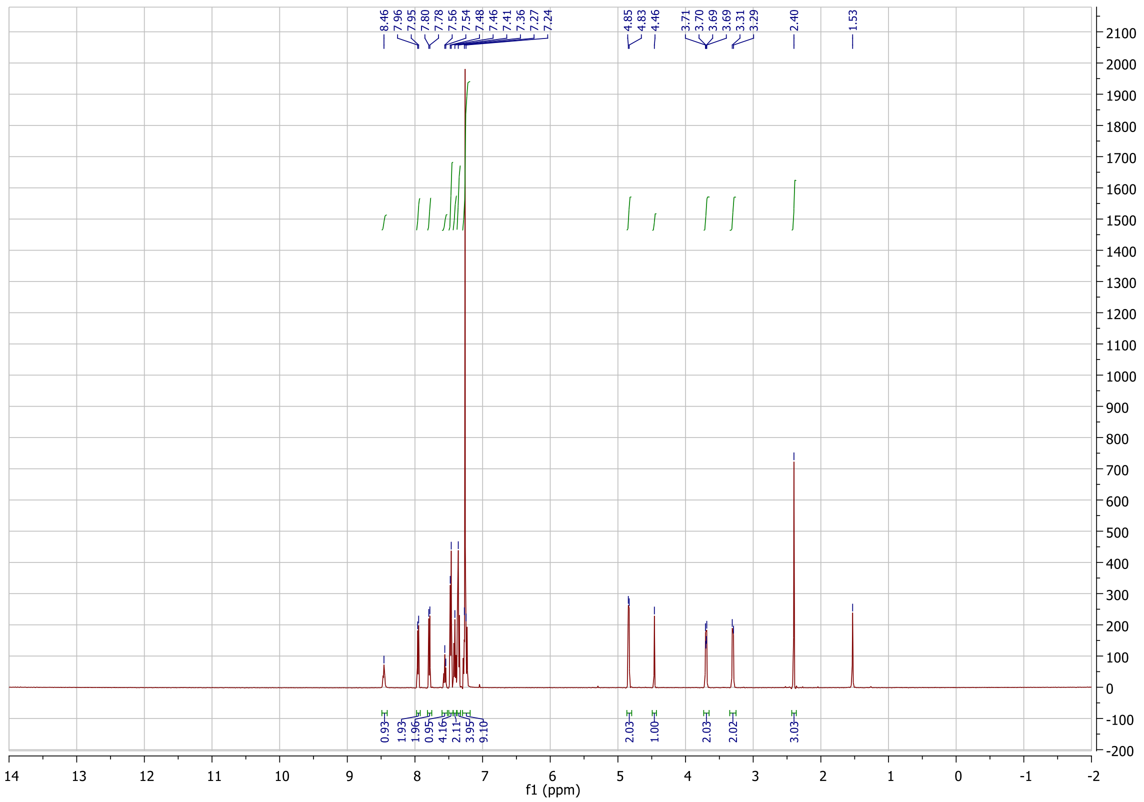

^{13}C NMR spectra of1-benzhydryl-3-((tosylmethyl)carbamoyl)azetidin-3-yl benzoate 4a

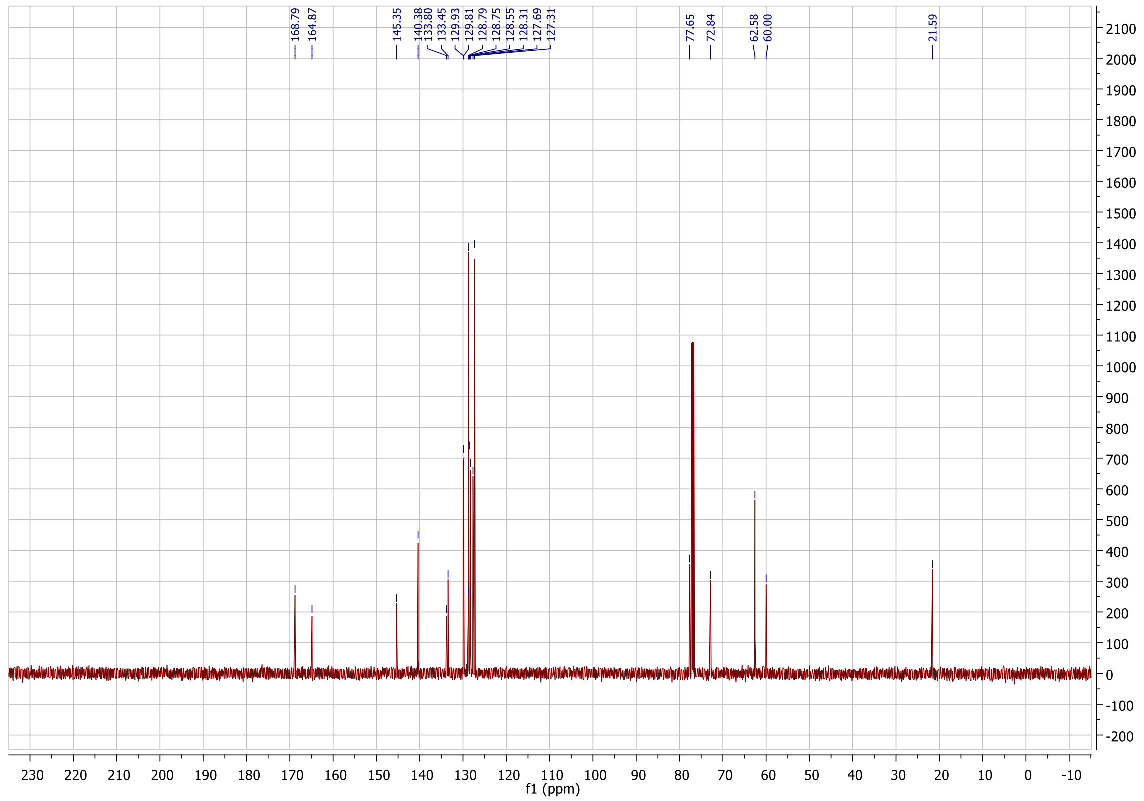

^{1}H NMR spectra of tert-butyl 3-(benzoyloxy)-3-((tosylmethyl)carbamoyl)azetidine-1-carboxylate 4b

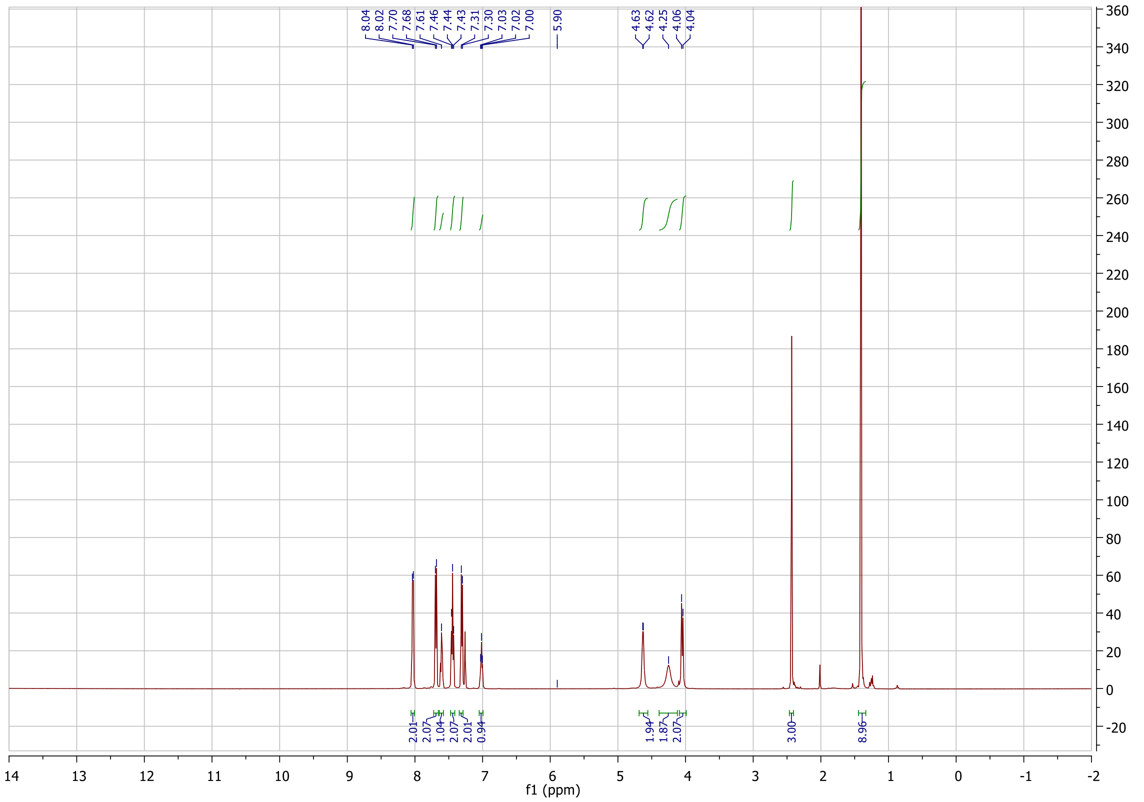

^{13}C NMR spectra of tert-butyl 3-(benzoyloxy)-3-((tosylmethyl)carbamoyl)azetidine-1-carboxylate 4b

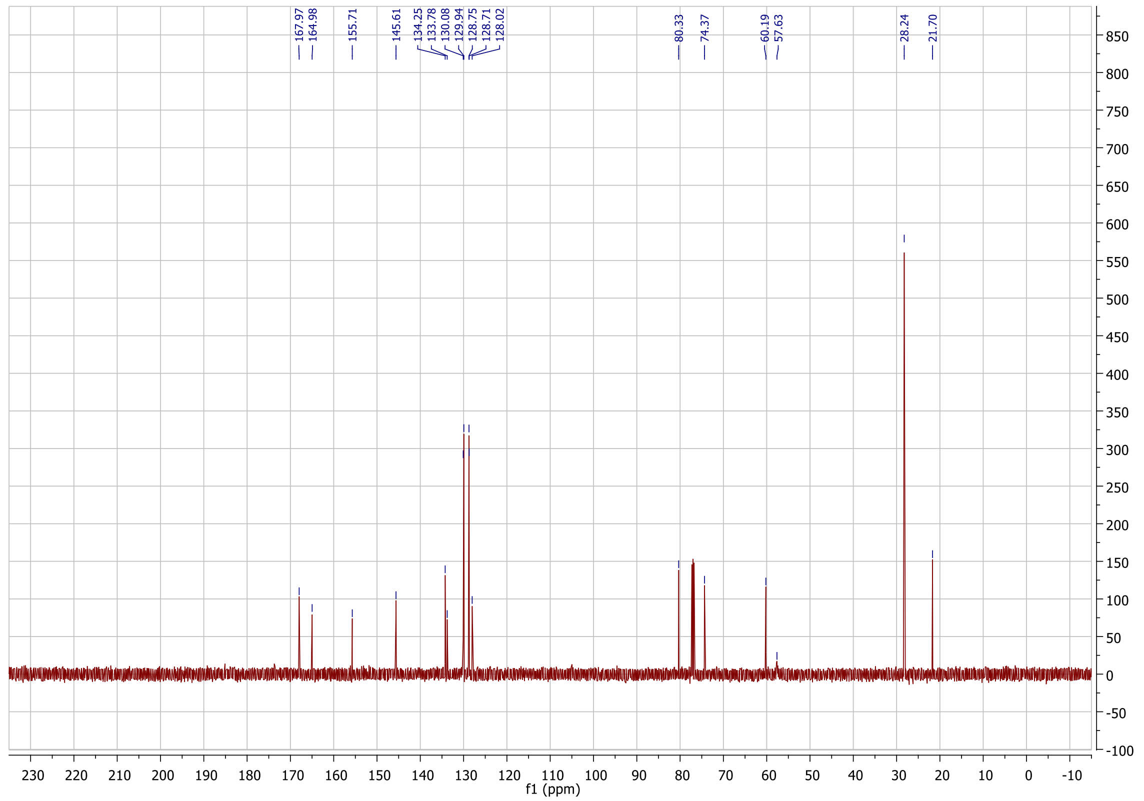

^{1}H NMR spectra of 3-((tosylmethyl)carbamoyl)oxetan-3-yl benzoate 4c

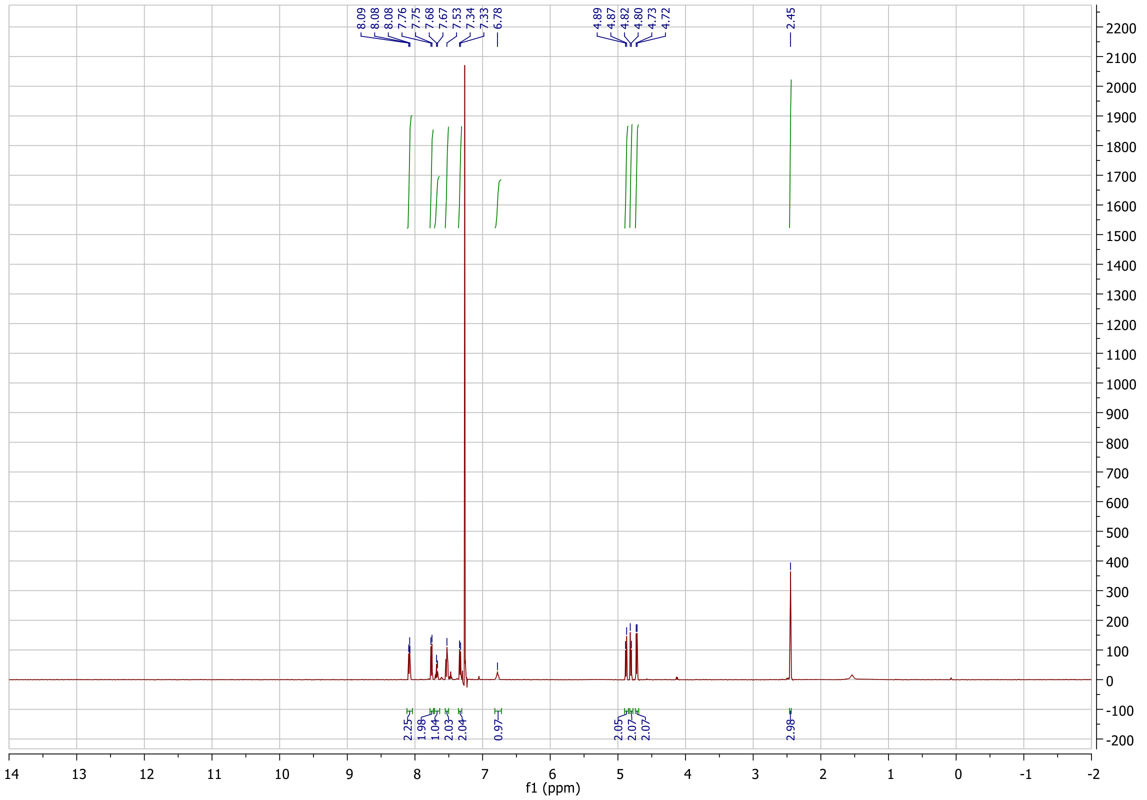

^{13}C NMR spectra of 3-((tosylmethyl)carbamoyl)oxetan-3-yl benzoate 4c

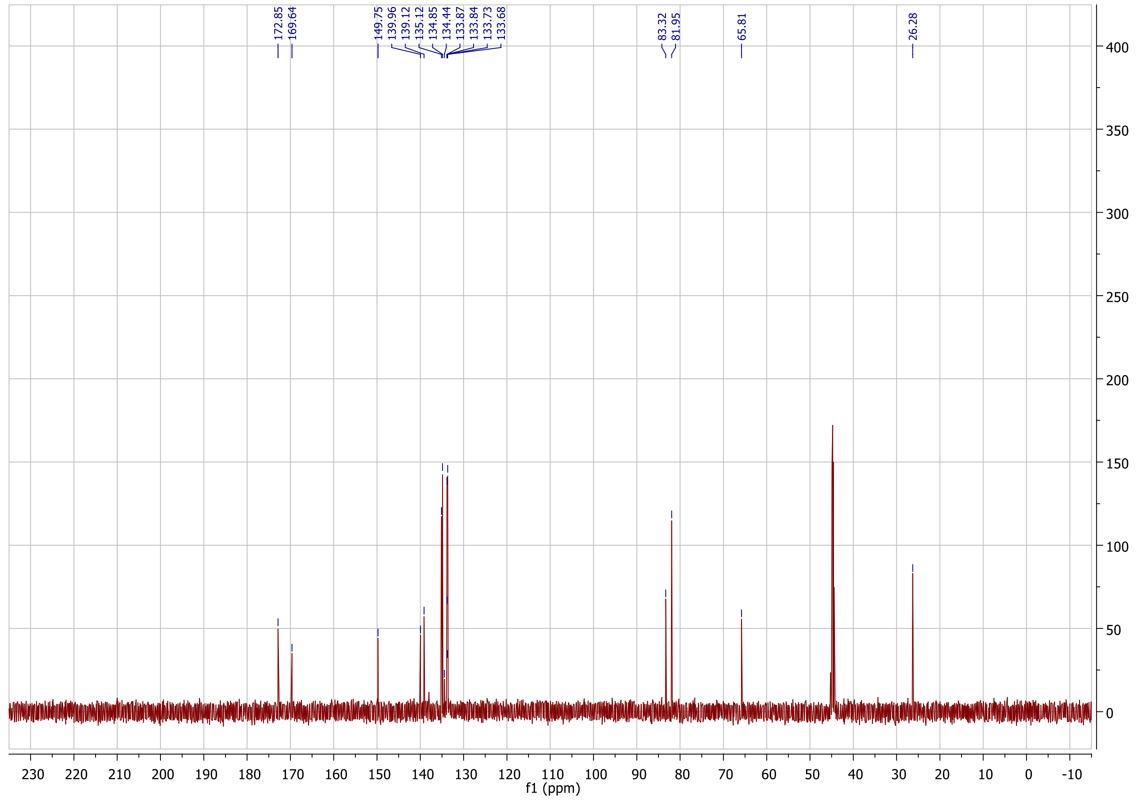

^{1}H NMR spectra of 3-((tosylmethyl)carbamoyl)thietan-3-yl benzoate 4d

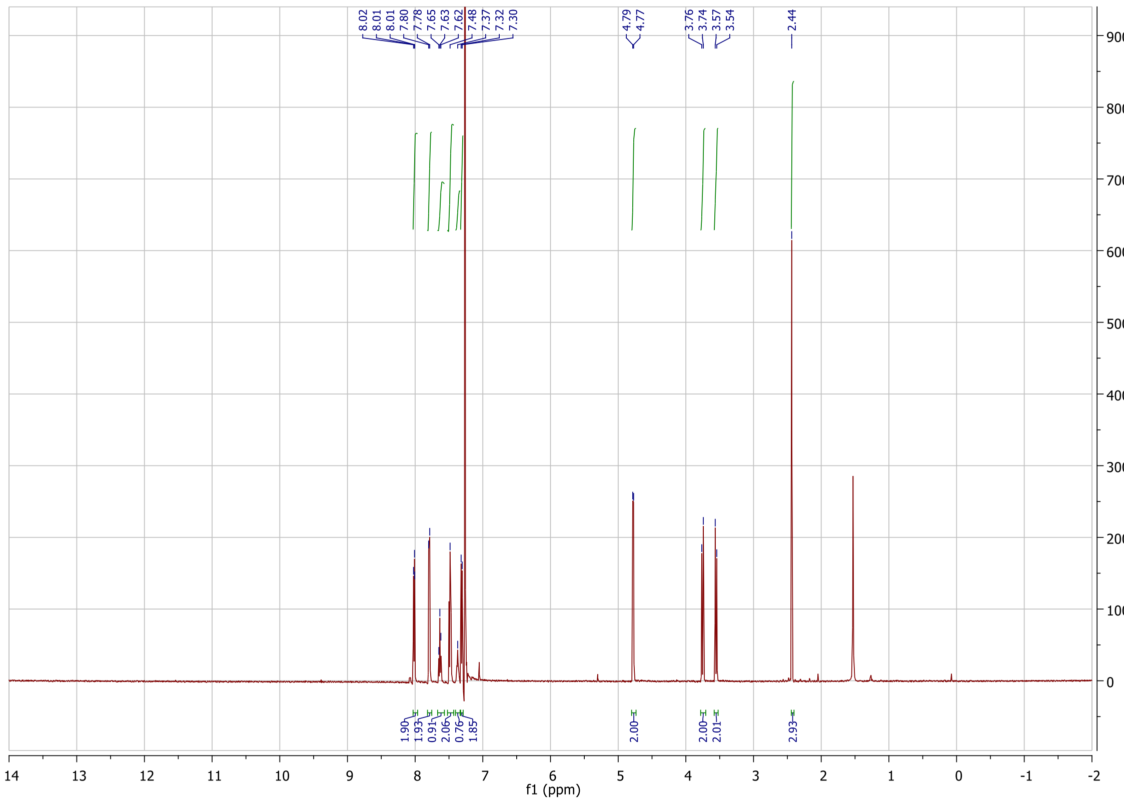

^{13}C NMR spectra of 3-((tosylmethyl)carbamoyl)thietan-3-yl benzoate 4d

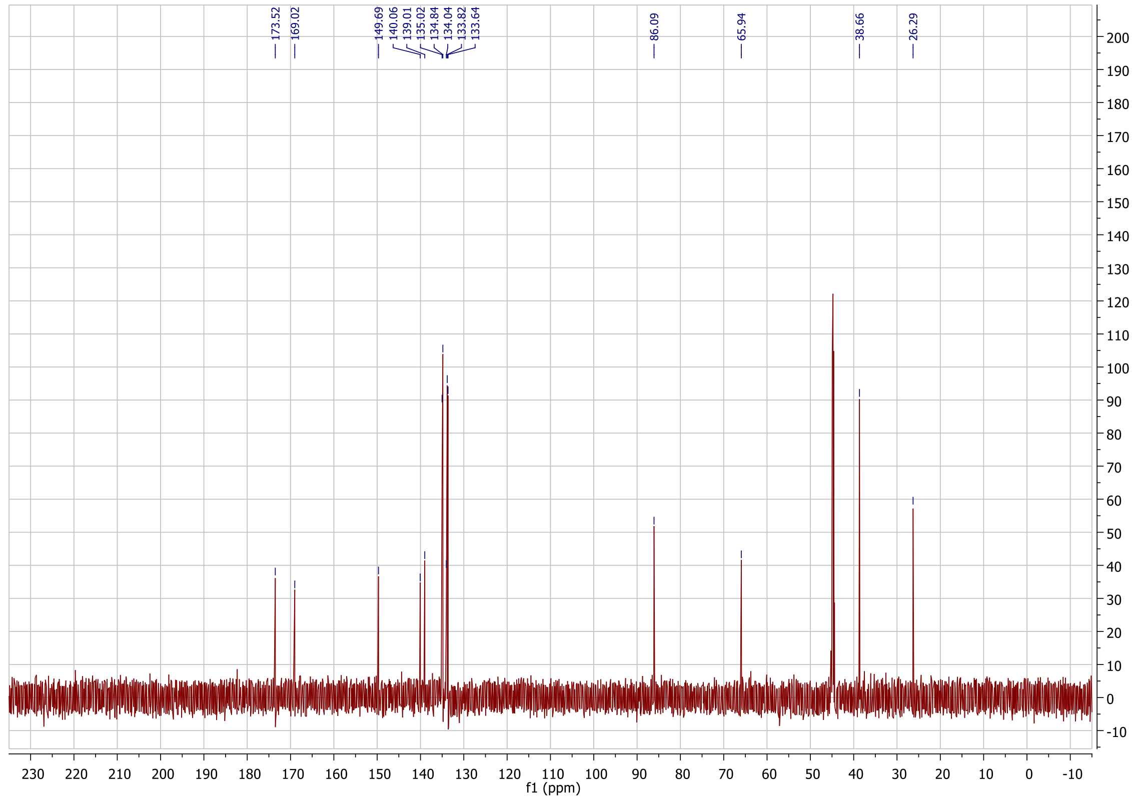

^{1}H NMR spectra of 1-benzhydryl-3-((4-bromophenyl)carbamoyl)azetidin-3-yl benzoate 4e

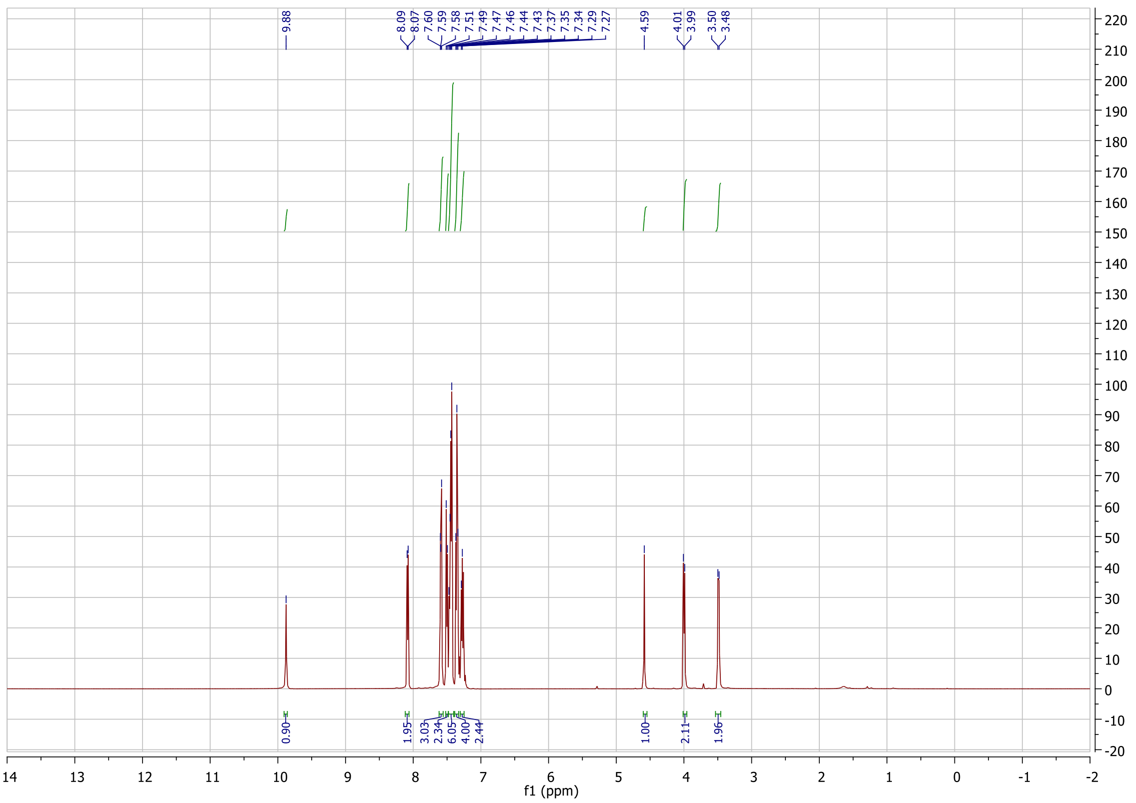

^{13}C NMR spectra of 1-benzhydryl-3-((4-bromophenyl)carbamoyl)azetidin-3-yl benzoate 4e

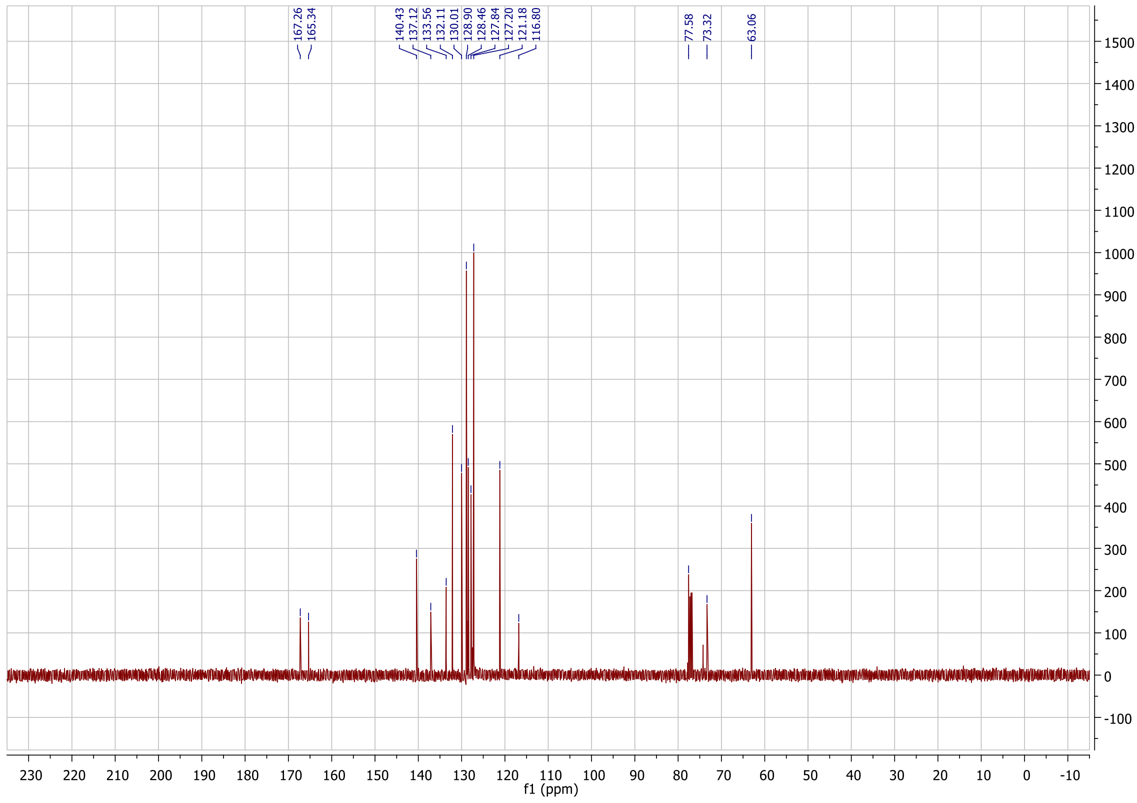

¹H NMR spectra of 1-benzhydryl-3-((4-methoxyphenyl)carbamoyl)azetidin-3-yl benzoate 4f

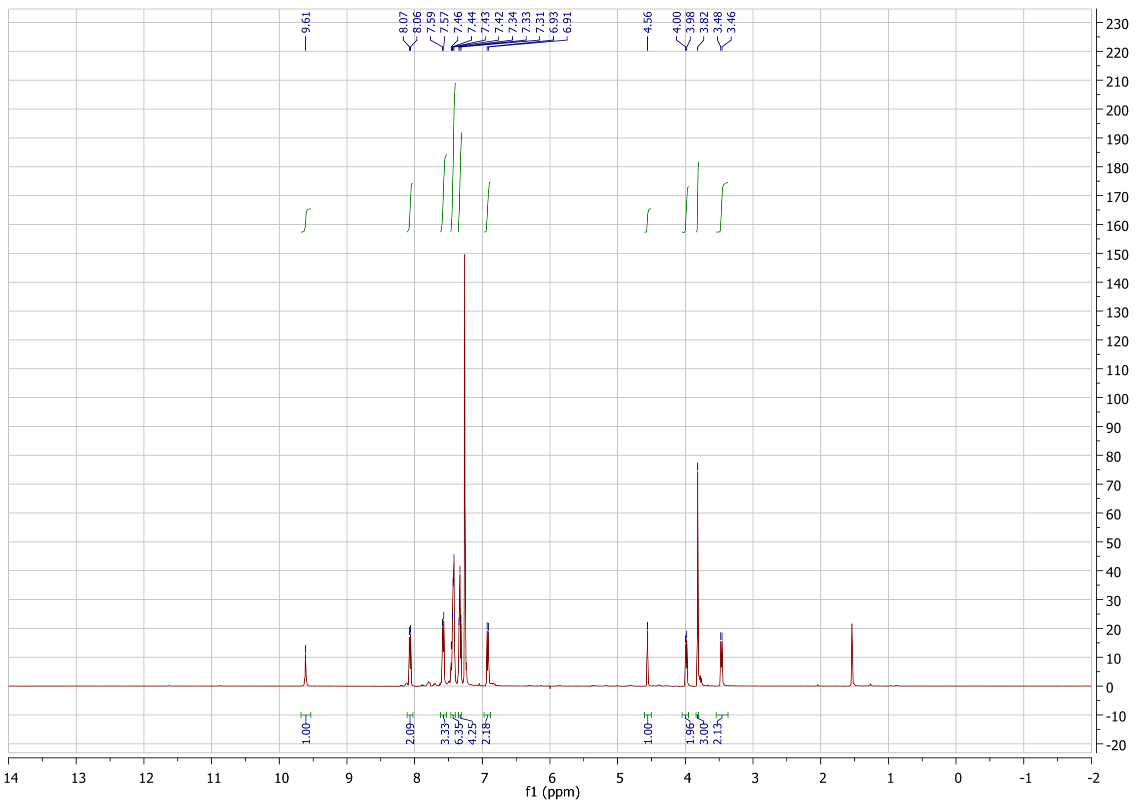

¹³C NMR spectra of 1-benzhydryl-3-((4-methoxyphenyl)carbamoyl)azetidin-3-yl benzoate 4f

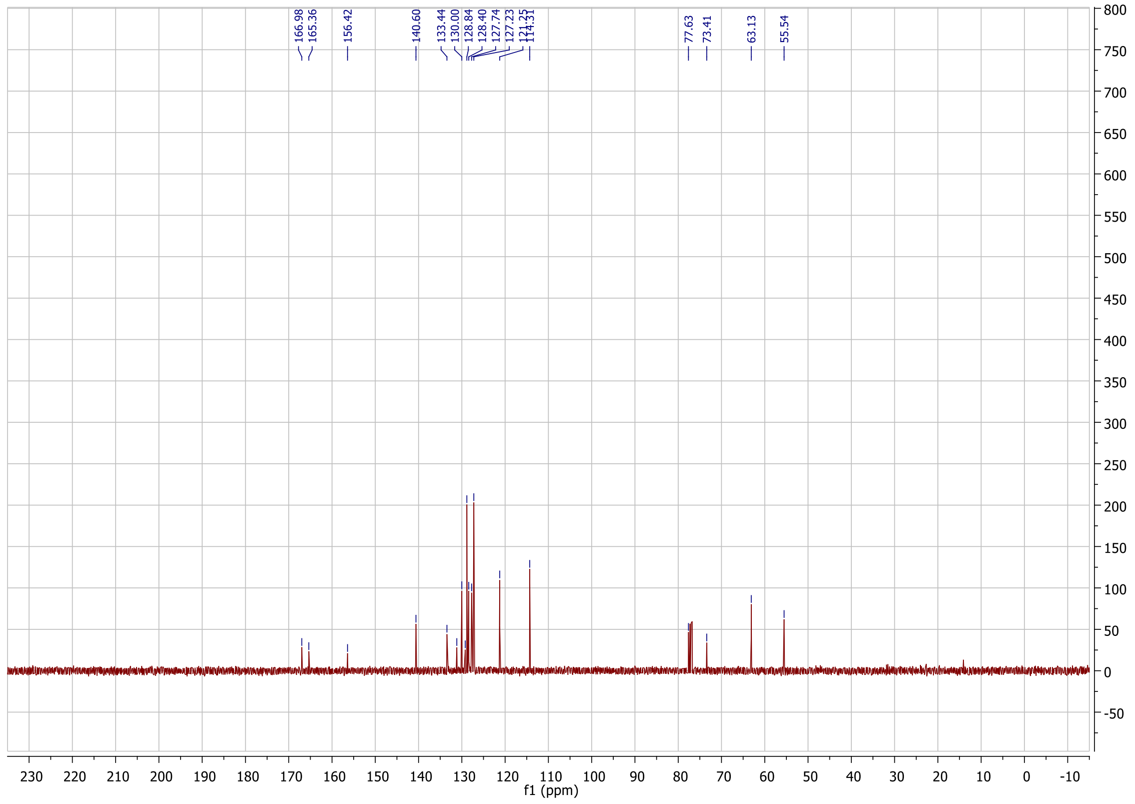

^{1}H NMR spectra of 1-benzhydryl-3-(benzylcarbamoyl)azetidin-3-yl benzoate 4g

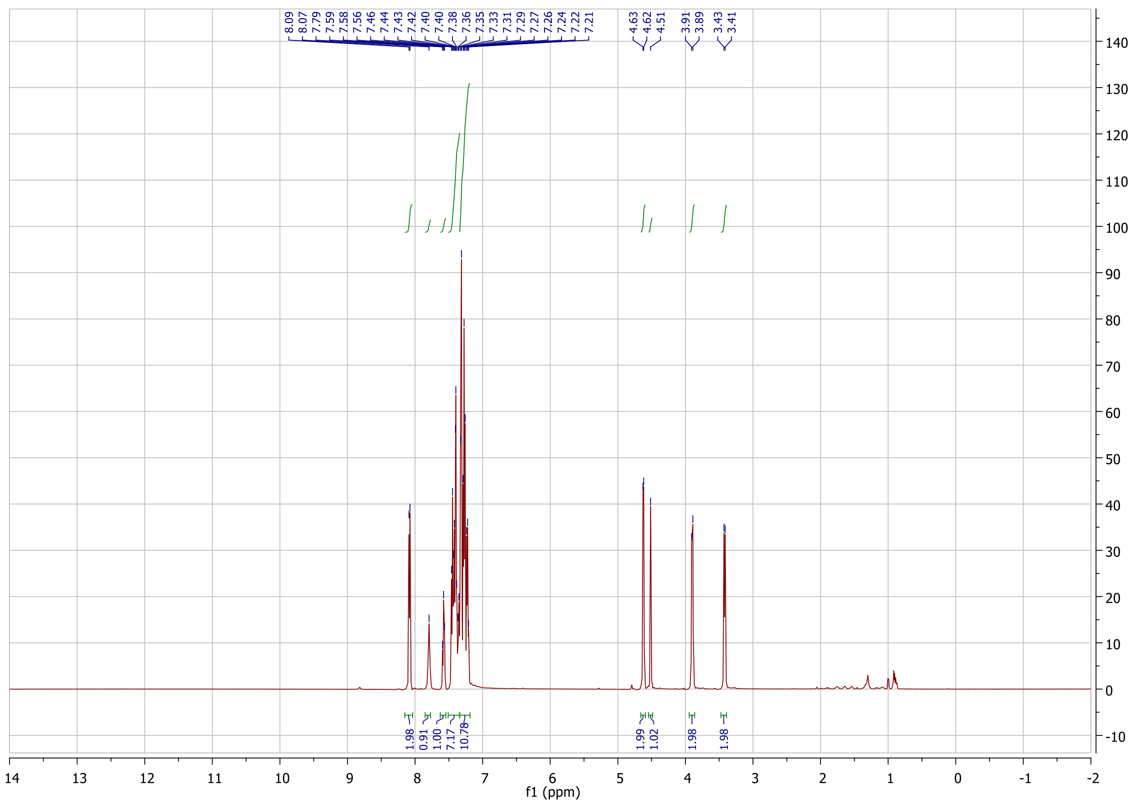

^{13}C NMR spectra of1-benzhydryl-3-(benzylcarbamoyl)azetidin-3-yl benzoate 4g

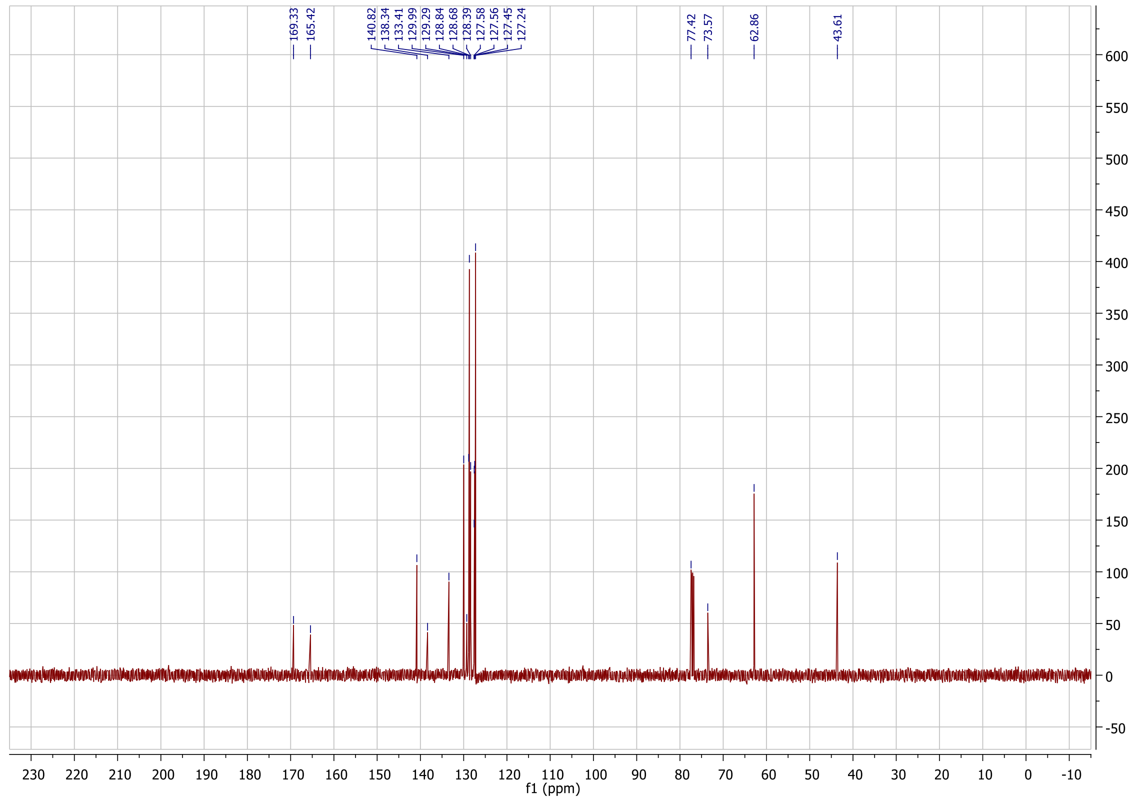

^{1}H NMR spectra of 1-benzhydryl-3-((3,5-dichlorobenzyl)carbamoyl)azetidin-3-yl benzoate 4h

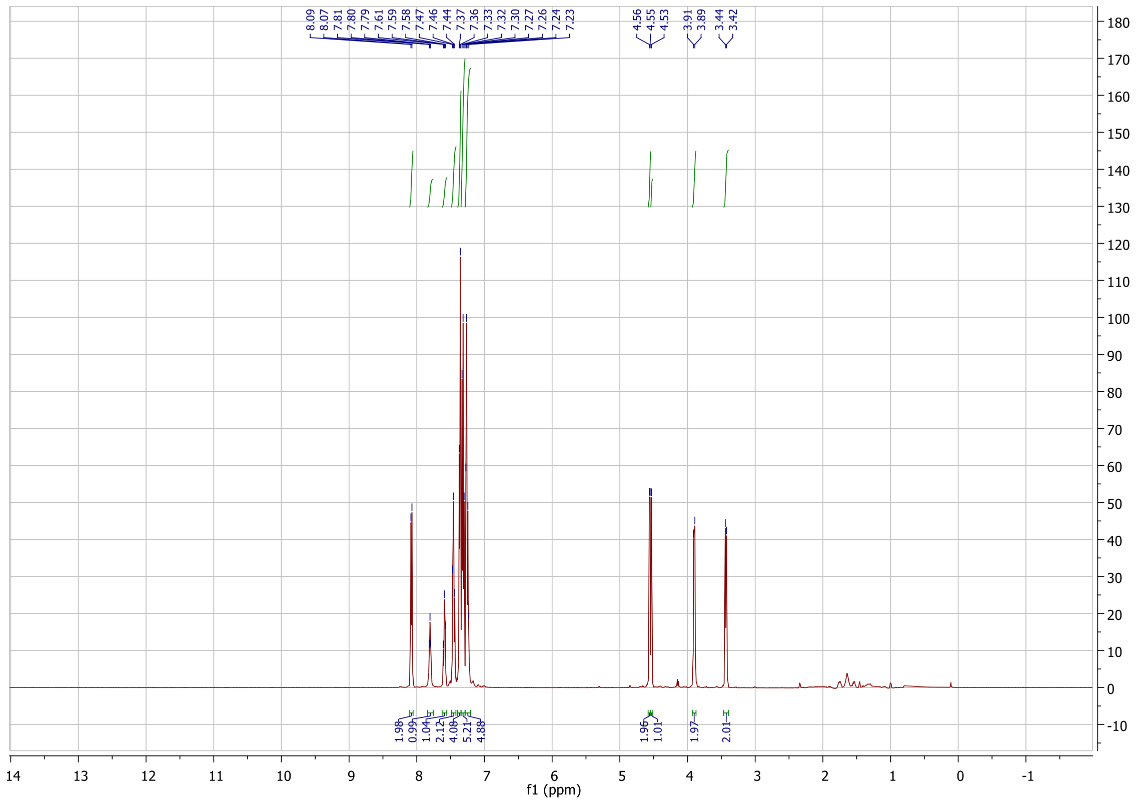

^{13}C NMR spectra of1-benzhydryl-3-((3,5-dichlorobenzyl)carbamoyl)azetidin-3-yl benzoate 4h

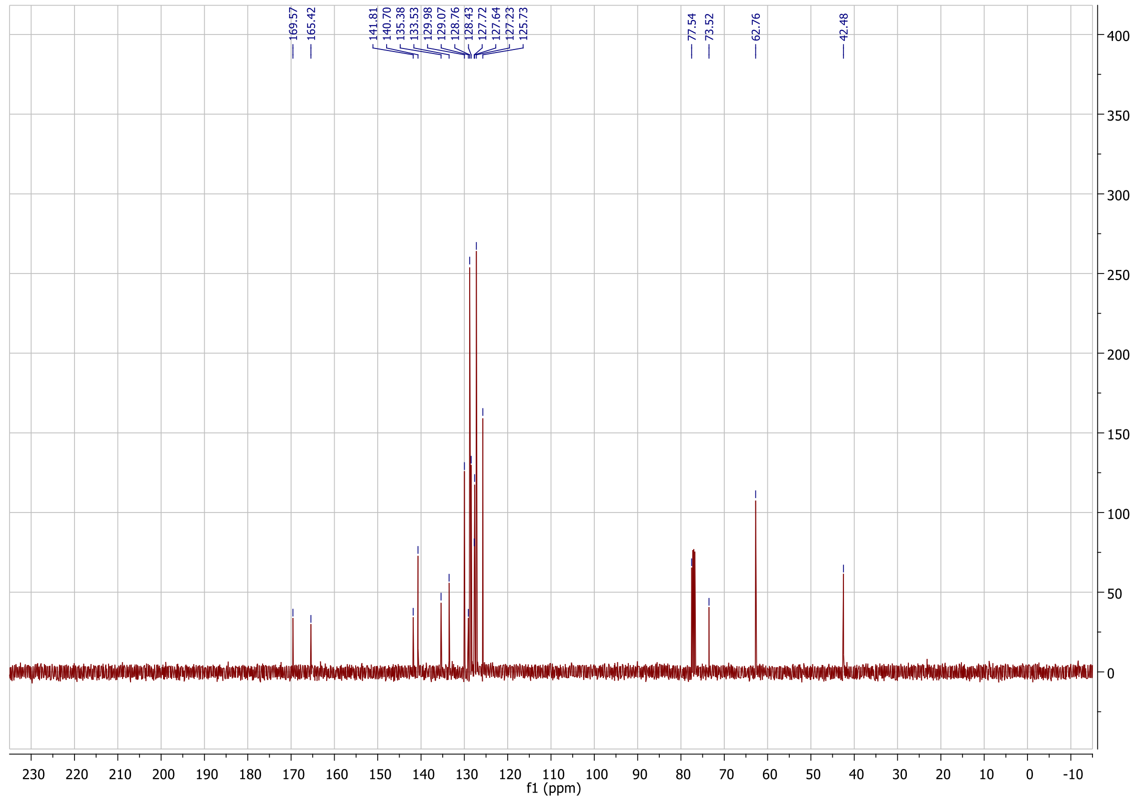

^{1}H NMR spectra of 1-benzhydryl-3-(cyclohexylcarbamoyl)azetidin-3-yl benzoate 4i

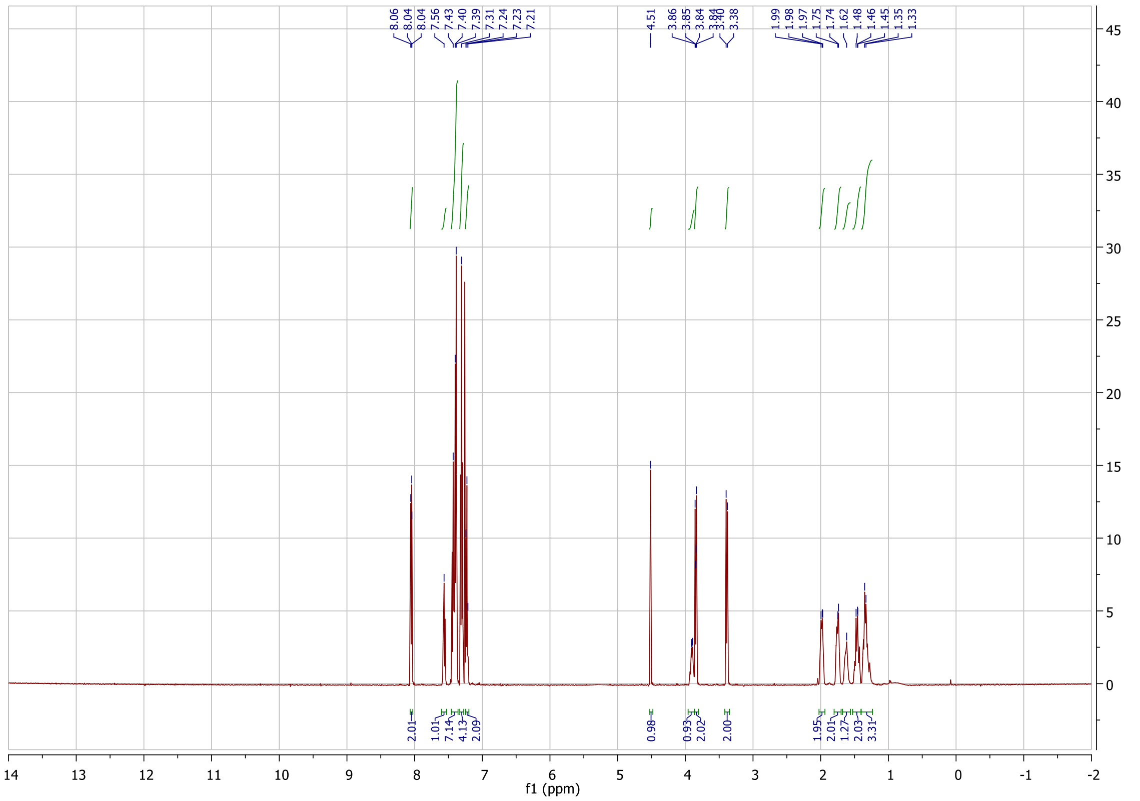

^{13}C NMR spectra of1-benzhydryl-3-(cyclohexylcarbamoyl)azetidin-3-yl benzoate 4i

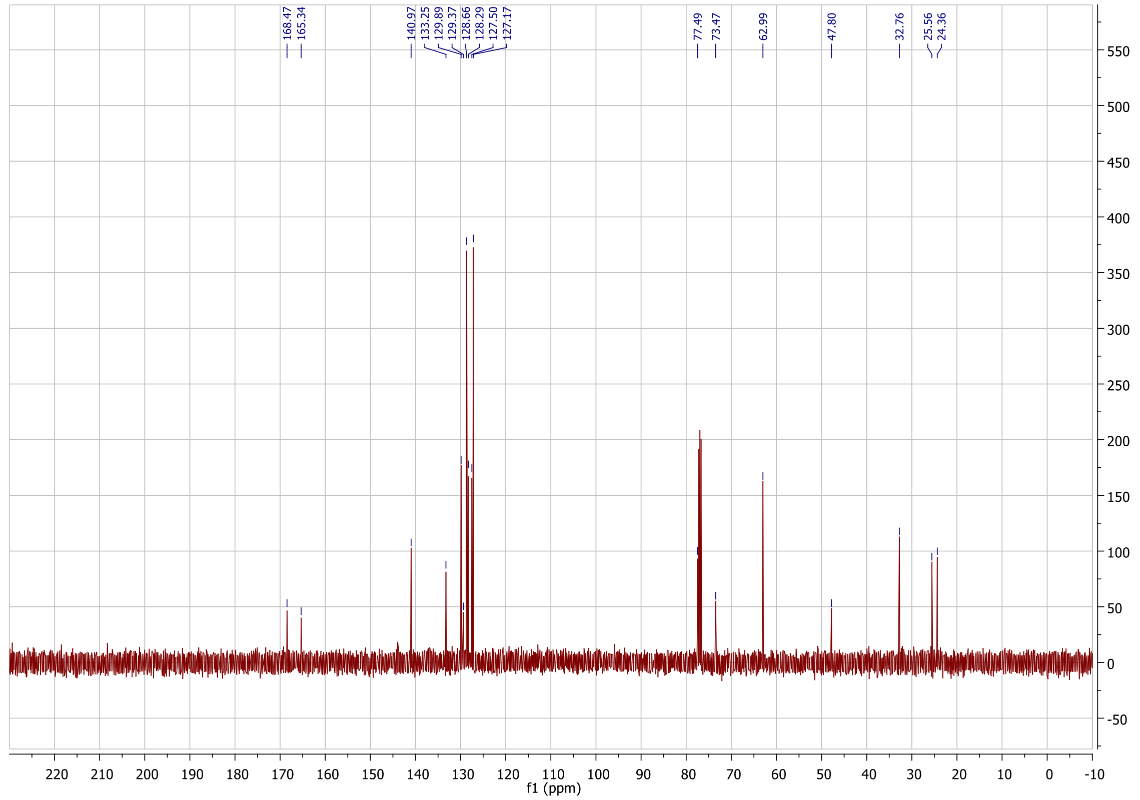

^{1}H NMR spectra of 1-benzhydryl-3-(butylcarbamoyl)azetidin-3-yl benzoate 4j

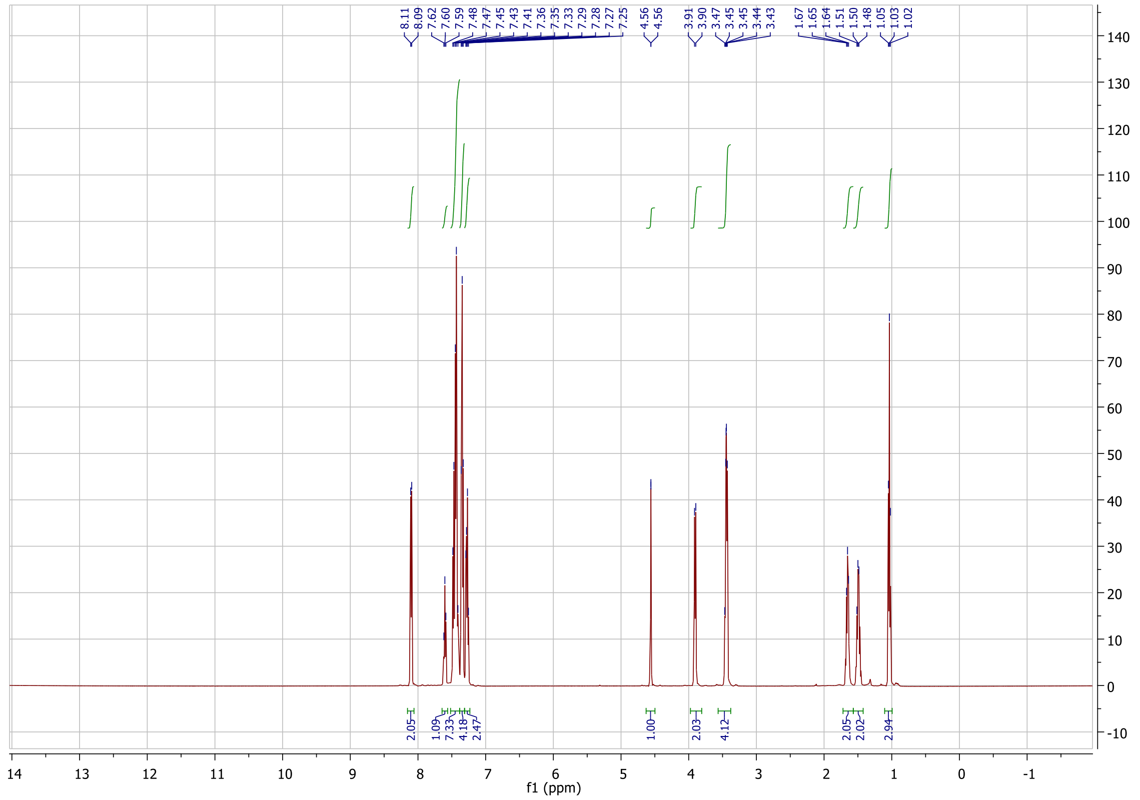

^{13}C NMR spectra of 1-benzhydryl-3-(butylcarbamoyl)azetidin-3-yl benzoate 4j

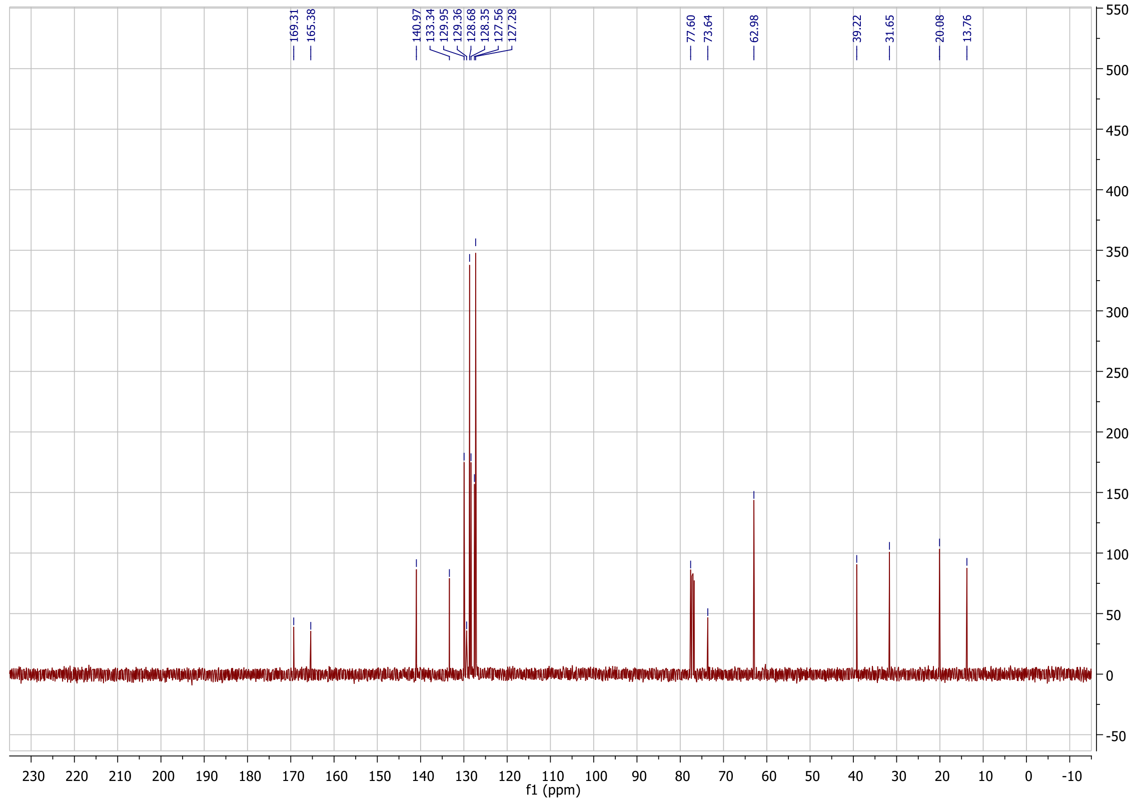

^{1}H NMR spectra of 1-benzhydryl-3-(tert-butylcarbamoyl)azetidin-3-yl benzoate 4k

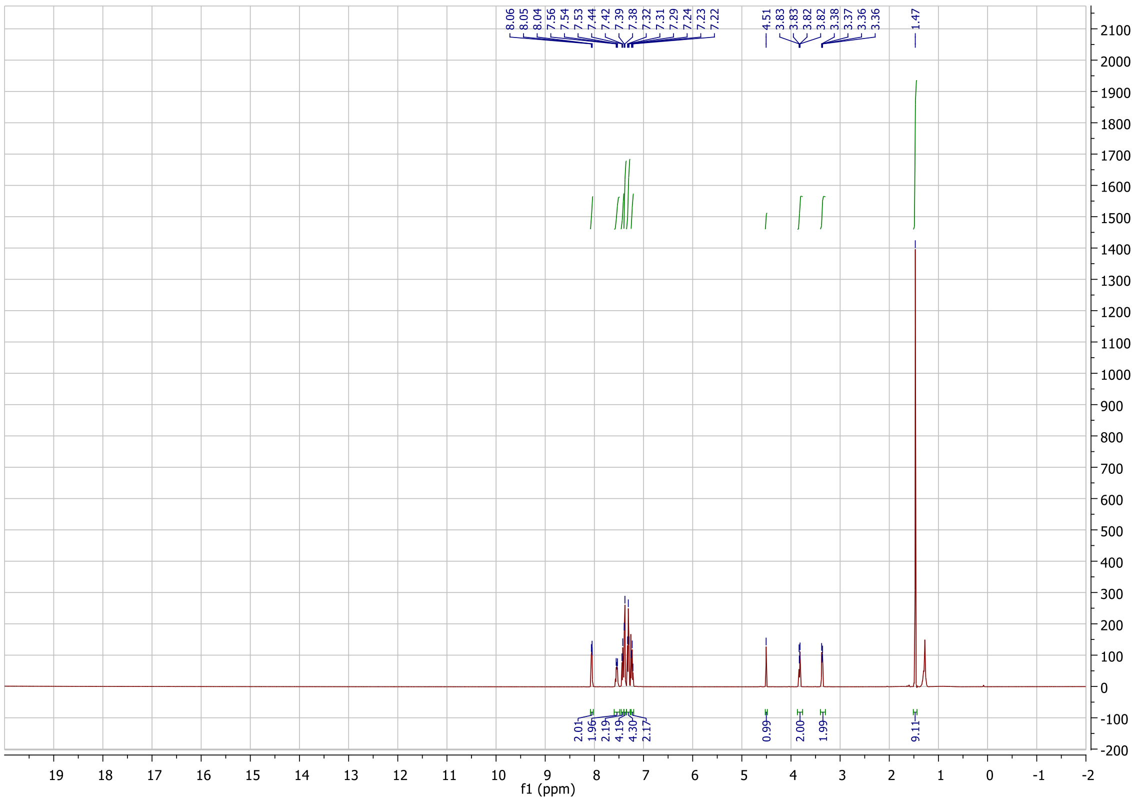

^{13}C NMR spectra of 1-benzhydryl-3-(tert-butylcarbamoyl)azetidin-3-yl benzoate 4k

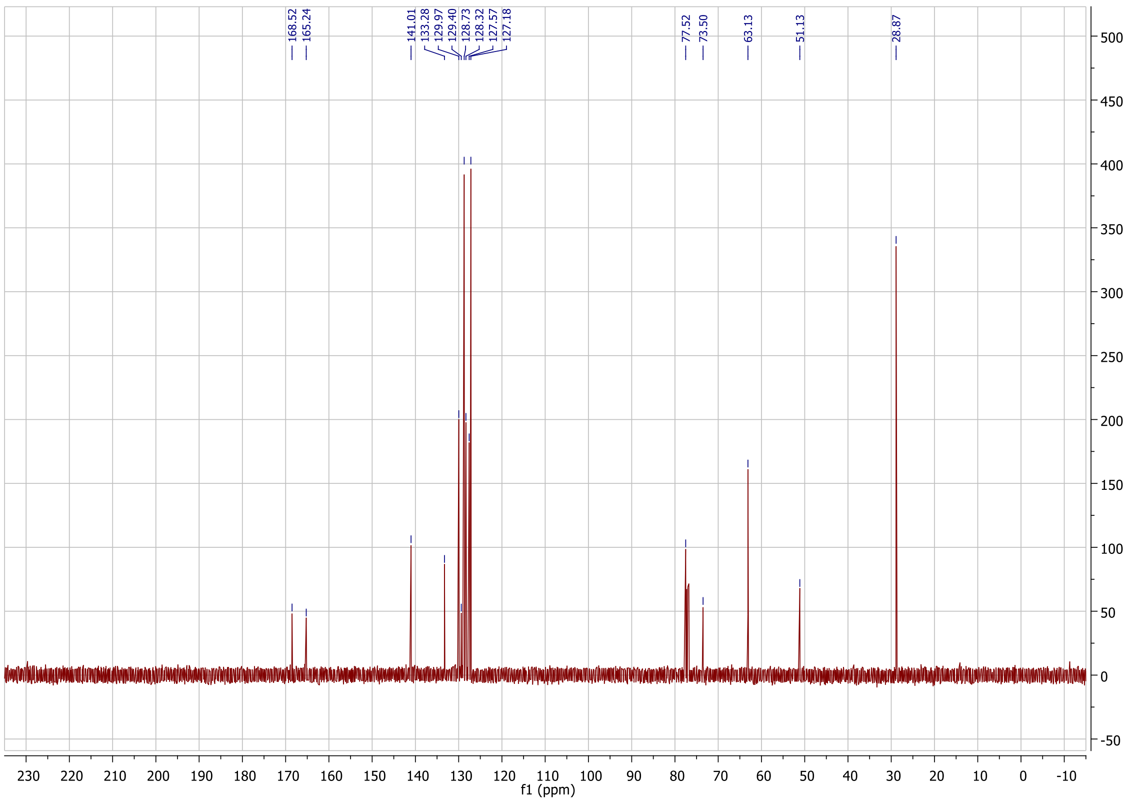

^{1}H NMR spectra of 1-benzhydryl-3-((tosylmethyl)carbamoyl)azetidin-3-yl 4-methylbenzoate 4l

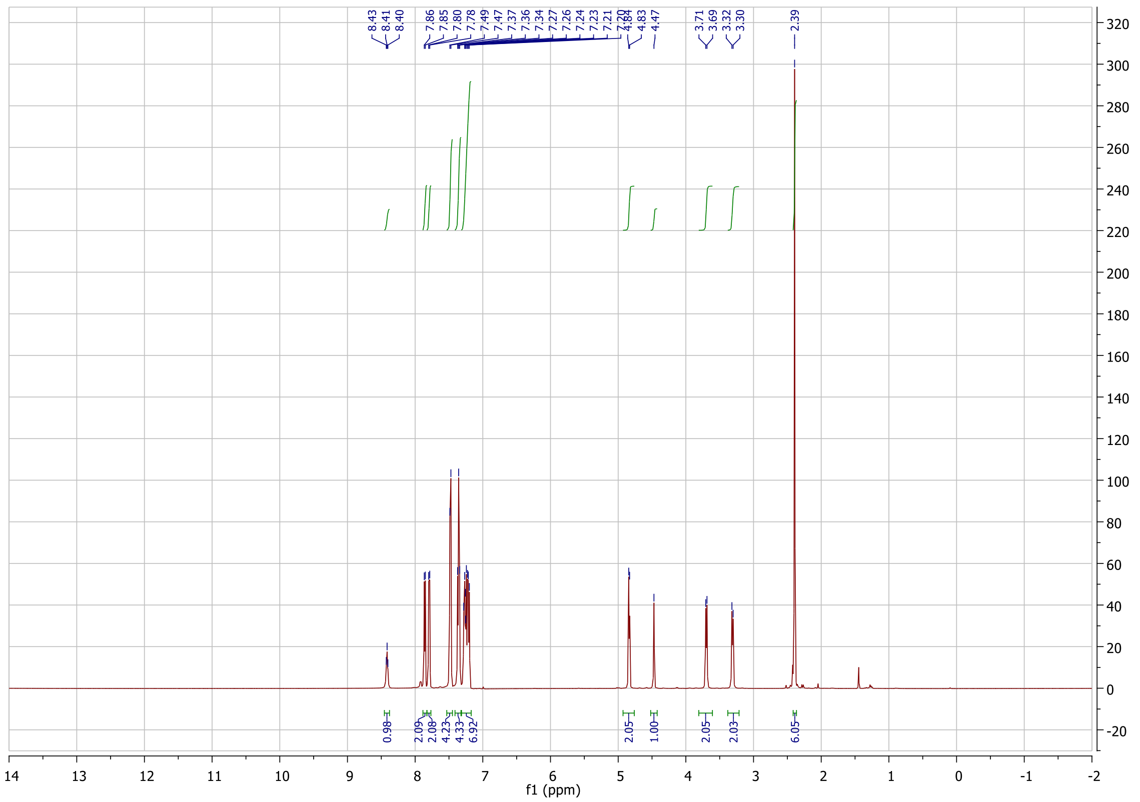

^{13}C NMR spectra of 1-benzhydryl-3-((tosylmethyl)carbamoyl)azetidin-3-yl 4-methylbenzoate 4l

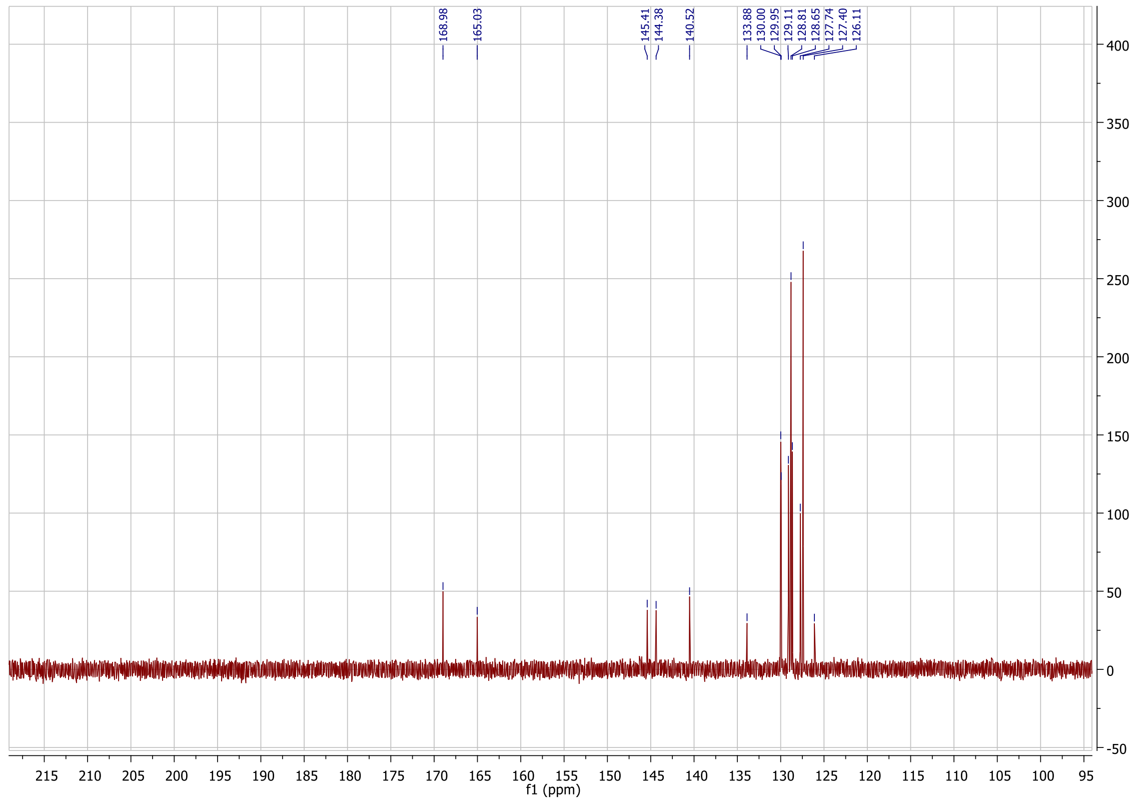

^{1}H NMR spectra of 1-benzhydryl-3-((tosylmethyl)carbamoyl)azetidin-3-yl 3-chlorobenzoate 4m

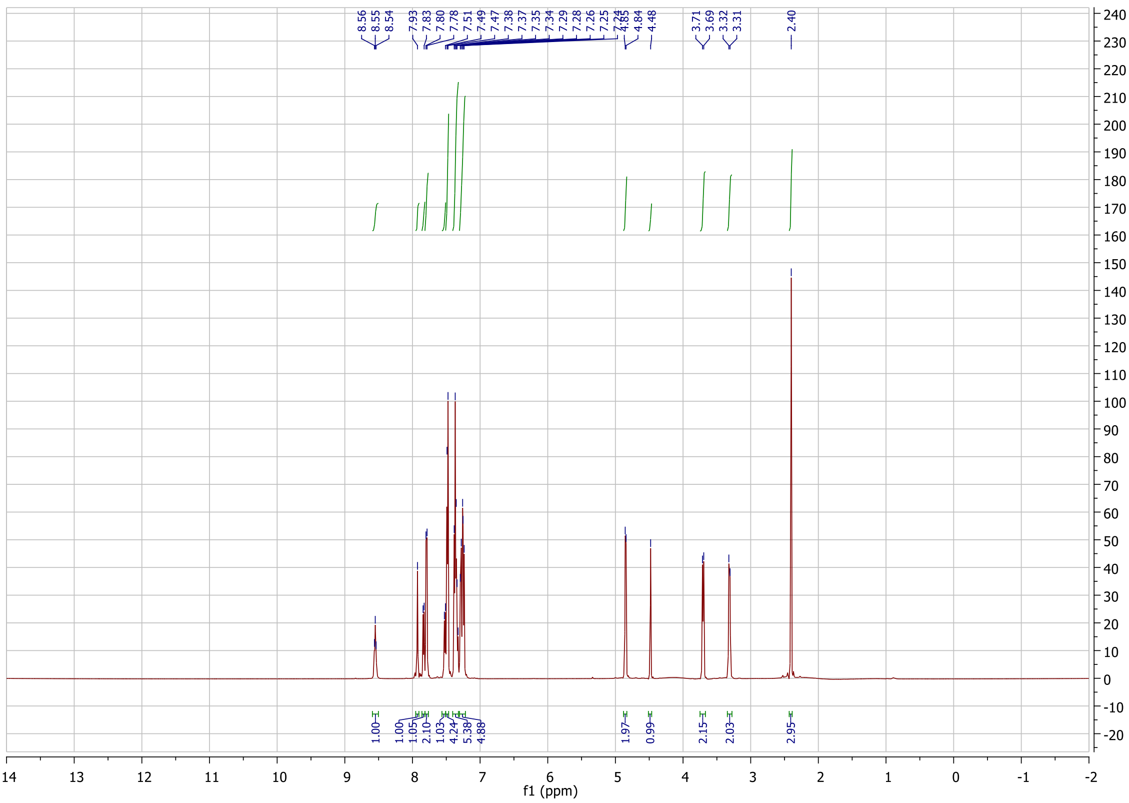

^{13}C NMR spectra of 1-benzhydryl-3-((tosylmethyl)carbamoyl)azetidin-3-yl 3-chlorobenzoate 4m

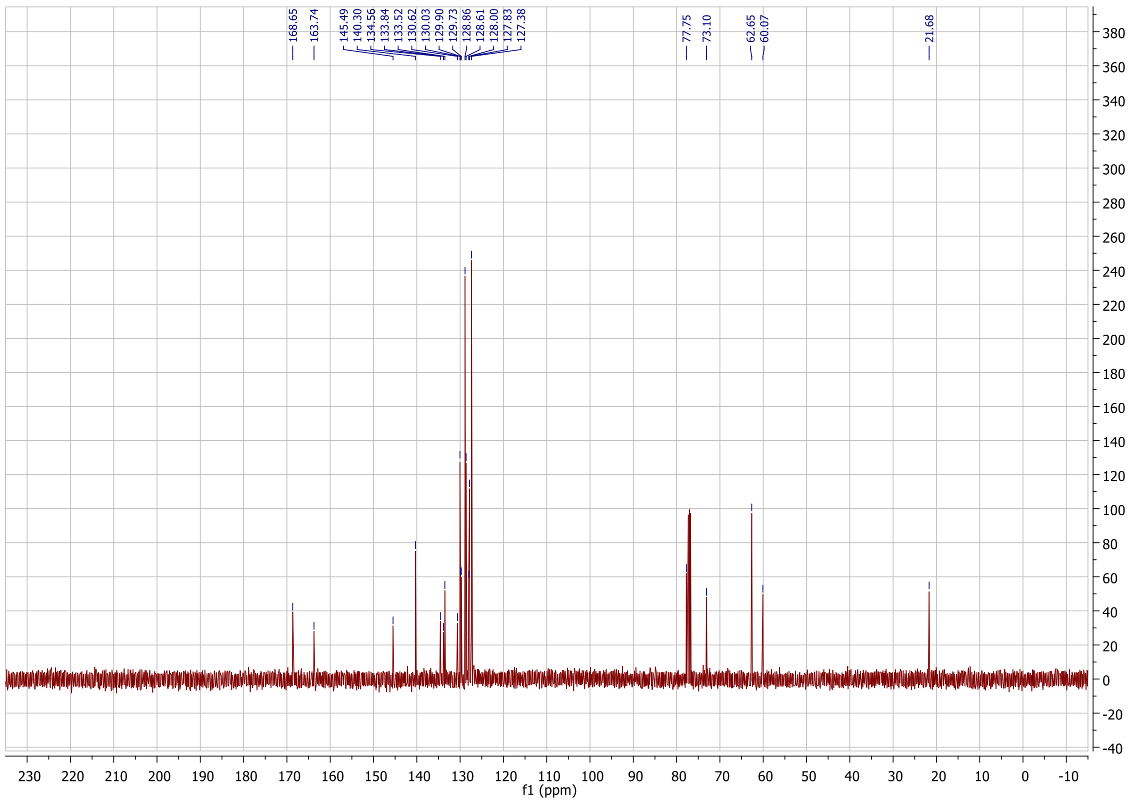

^{1}H NMR spectra of 1-benzhydryl-3-((tosylmethyl)carbamoyl)azetidin-3-yl 2-iodobenzoate 4n

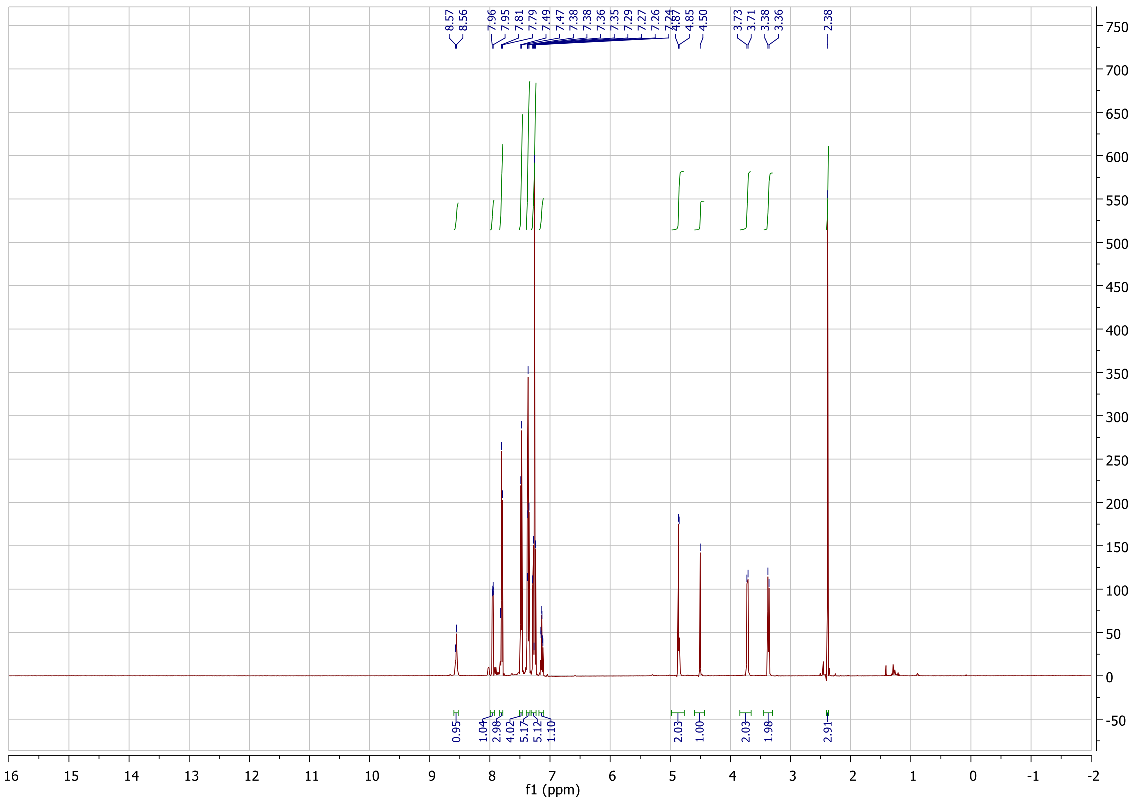

^{13}C NMR spectra of 1-benzhydryl-3-((tosylmethyl)carbamoyl)azetidin-3-yl 2-iodobenzoate 4n

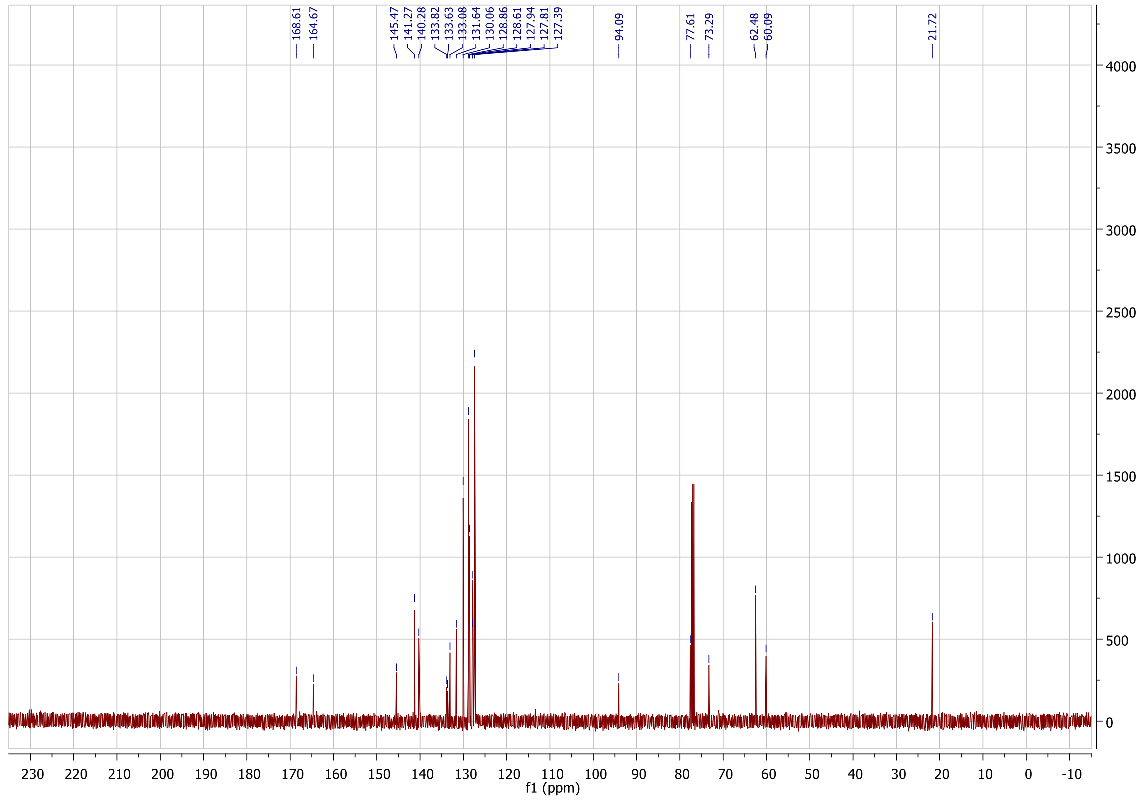

^{1}H NMR spectra of 1-benzhydryl-3-((tosylmethyl)carbamoyl)azetidin-3-yl 2-chlorobenzoate 4o

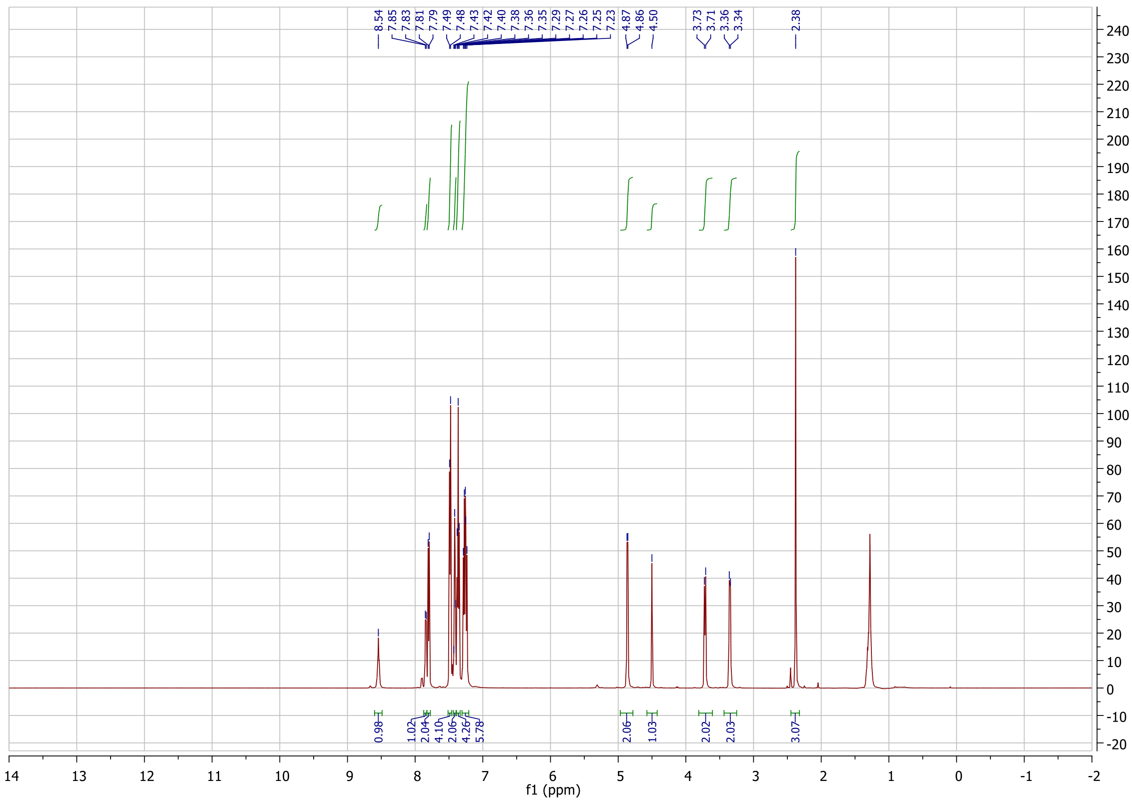

^{13}C NMR spectra of 1-benzhydryl-3-((tosylmethyl)carbamoyl)azetidin-3-yl 2-chlorobenzoate 4o

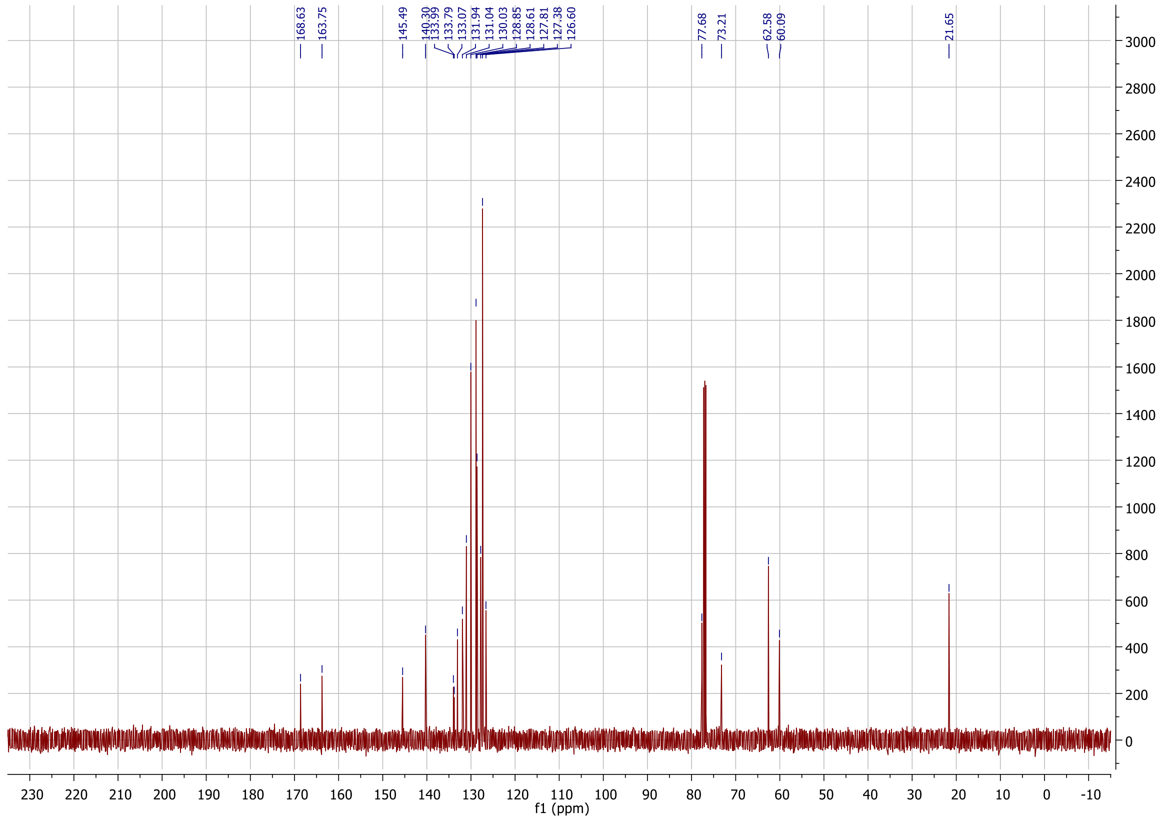

^{1}H NMR spectra of 1-benzhydryl-3-((tosylmethyl)carbamoyl)azetidin-3-yl 3,4-dichlorobenzoate 4p

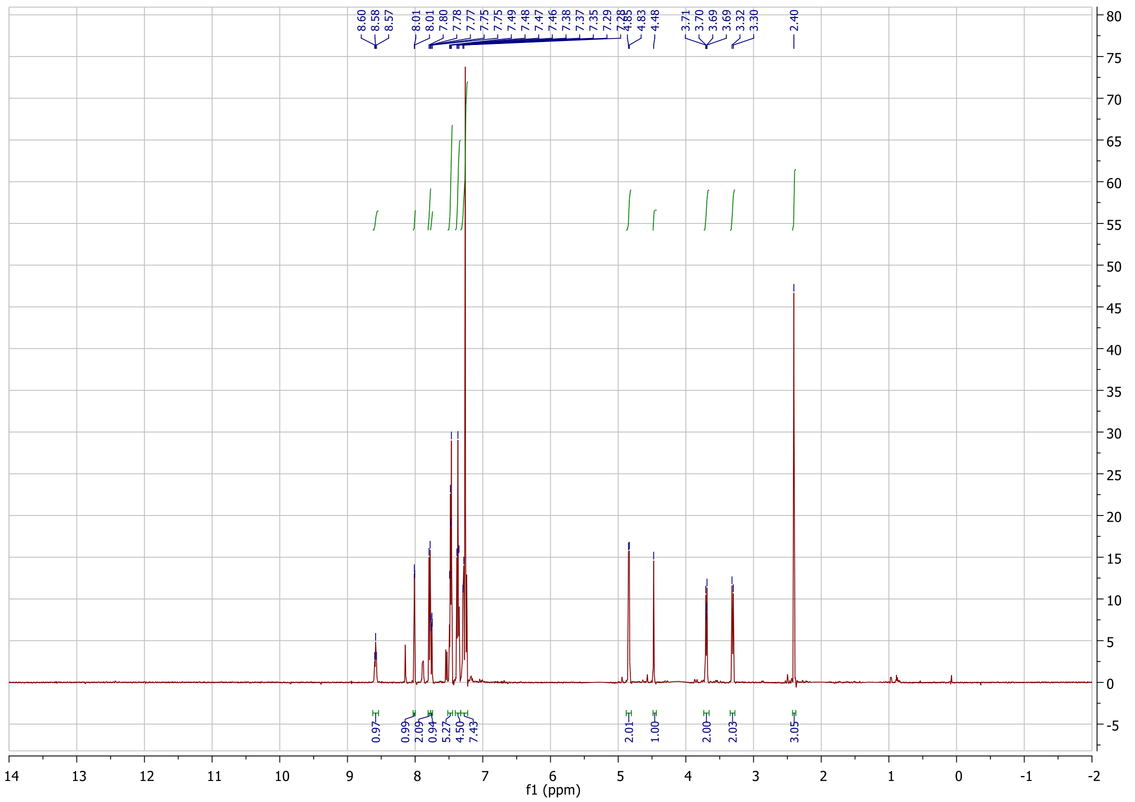

^{13}C NMR spectra of 1-benzhydryl-3-((tosylmethyl)carbamoyl)azetidin-3-yl 3,4-dichlorobenzoate 4p

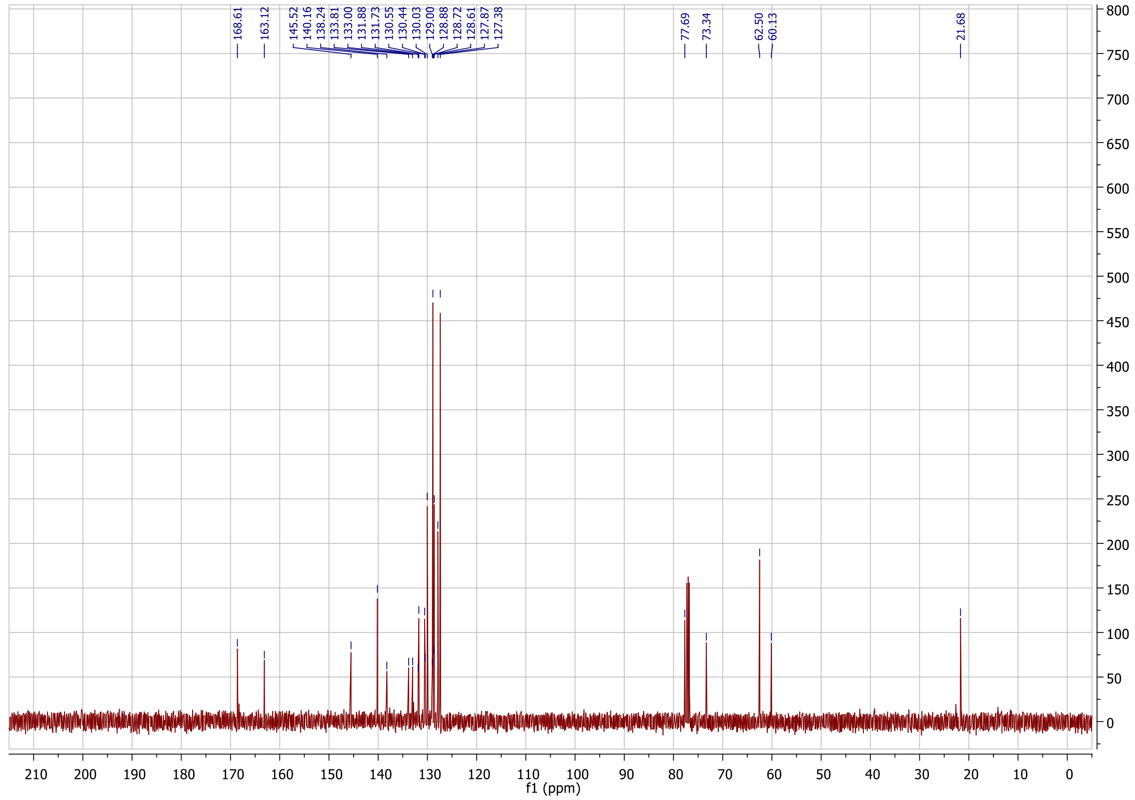

^{1}H NMR spectra of 1-benzhydryl-3-((tosylmethyl)carbamoyl)azetidin-3-yl 3,5-bis(trifluoromethyl)benzoate 4q

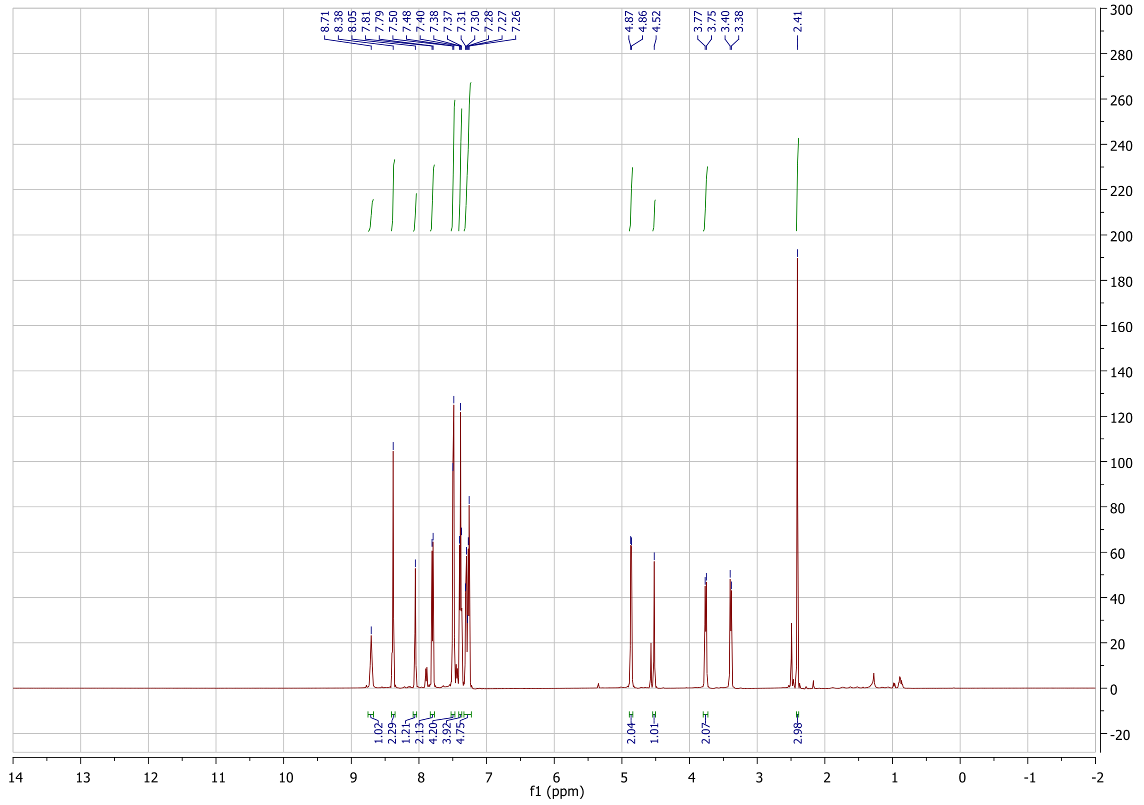

^{13}C NMR spectra of 1-benzhydryl-3-((tosylmethyl)carbamoyl)azetidin-3-yl 3,5-bis(trifluoromethyl)benzoate 4q

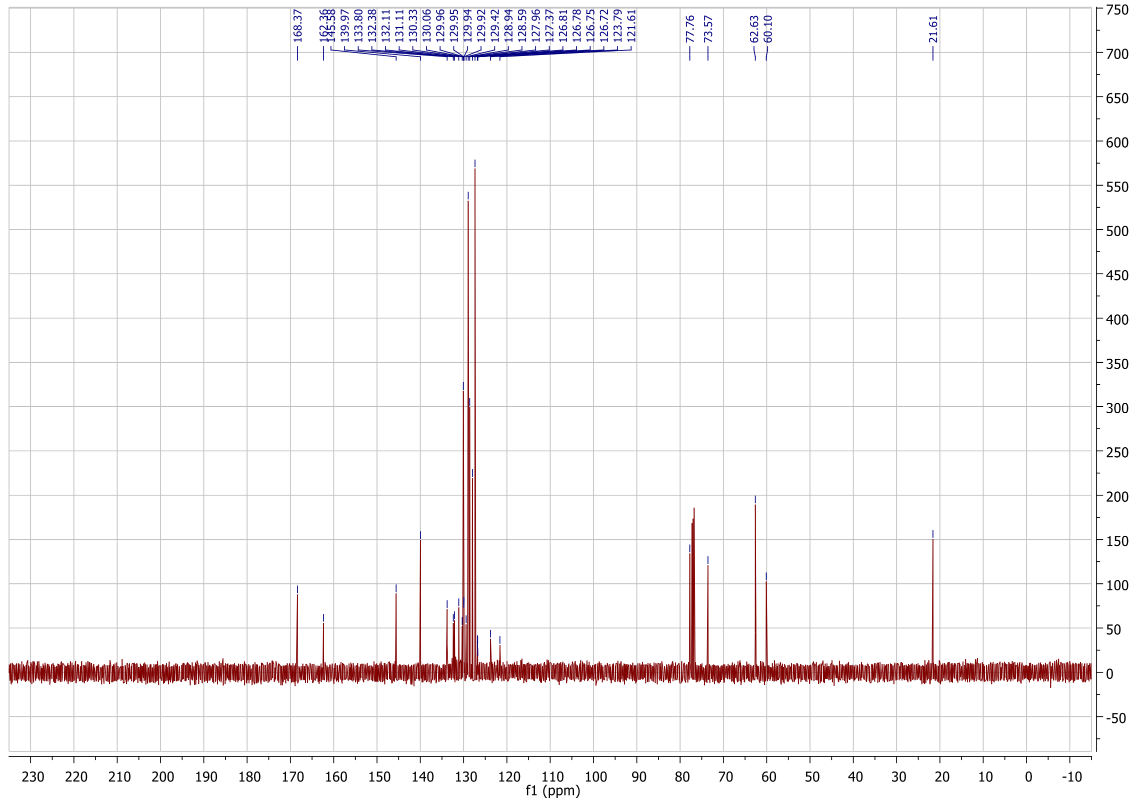

^{1}H NMR spectra of 1-benzhydryl-3-((tosylmethyl)carbamoyl)azetidin-3-yl furan-2-carboxylate 4r

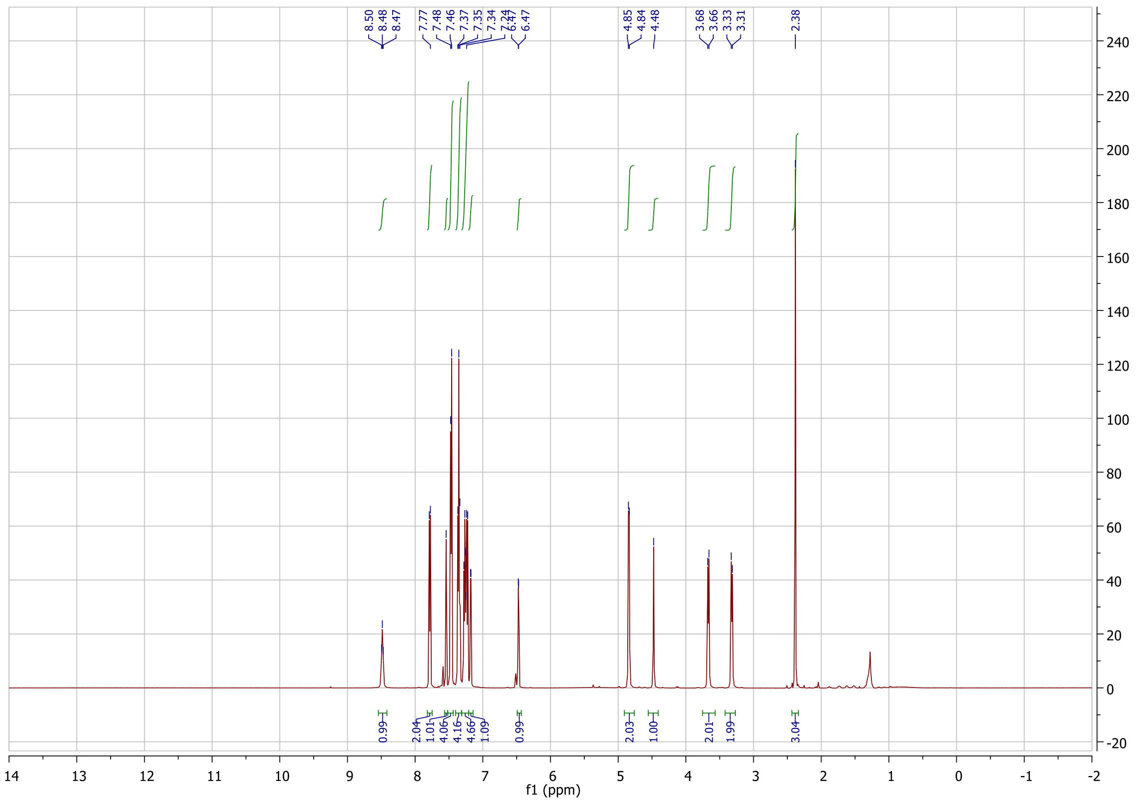

^{13}C NMR spectra of 1-benzhydryl-3-((tosylmethyl)carbamoyl)azetidin-3-yl furan-2-carboxylate 4r

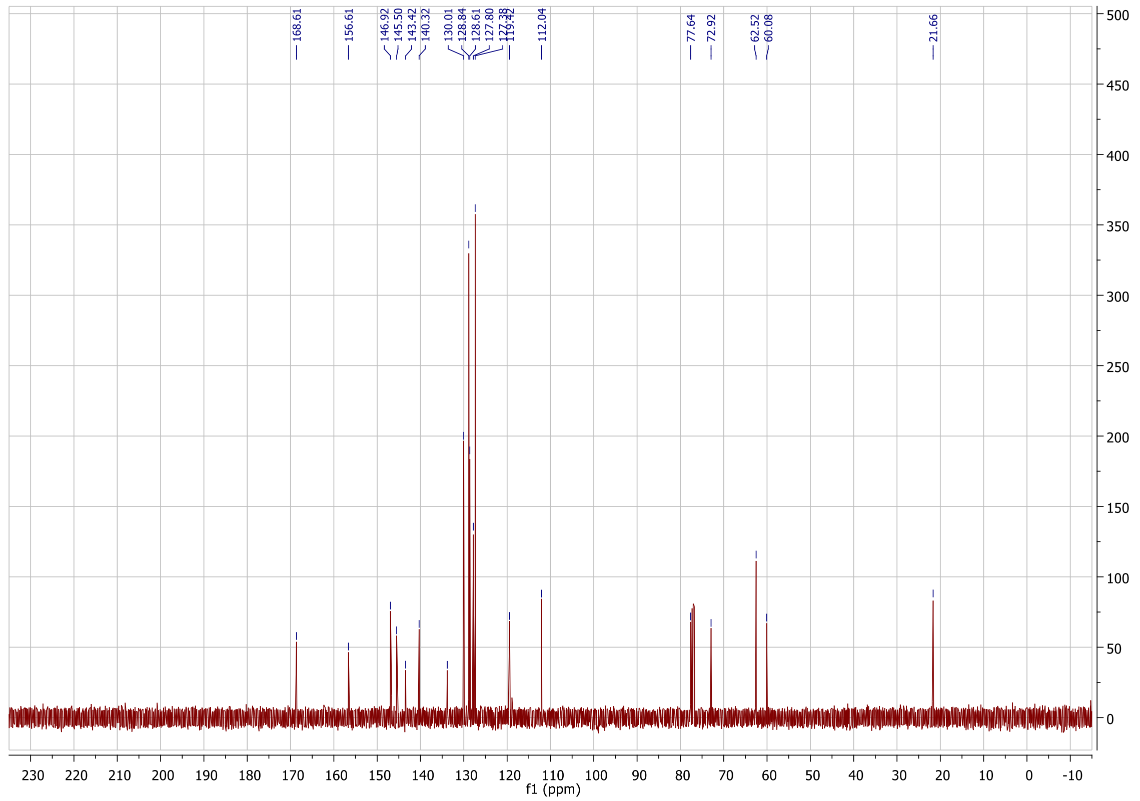

^{1}H NMR spectra of 1-benzhydryl-3-((tosylmethyl)carbamoyl)azetidin-3-yl acetate 4s

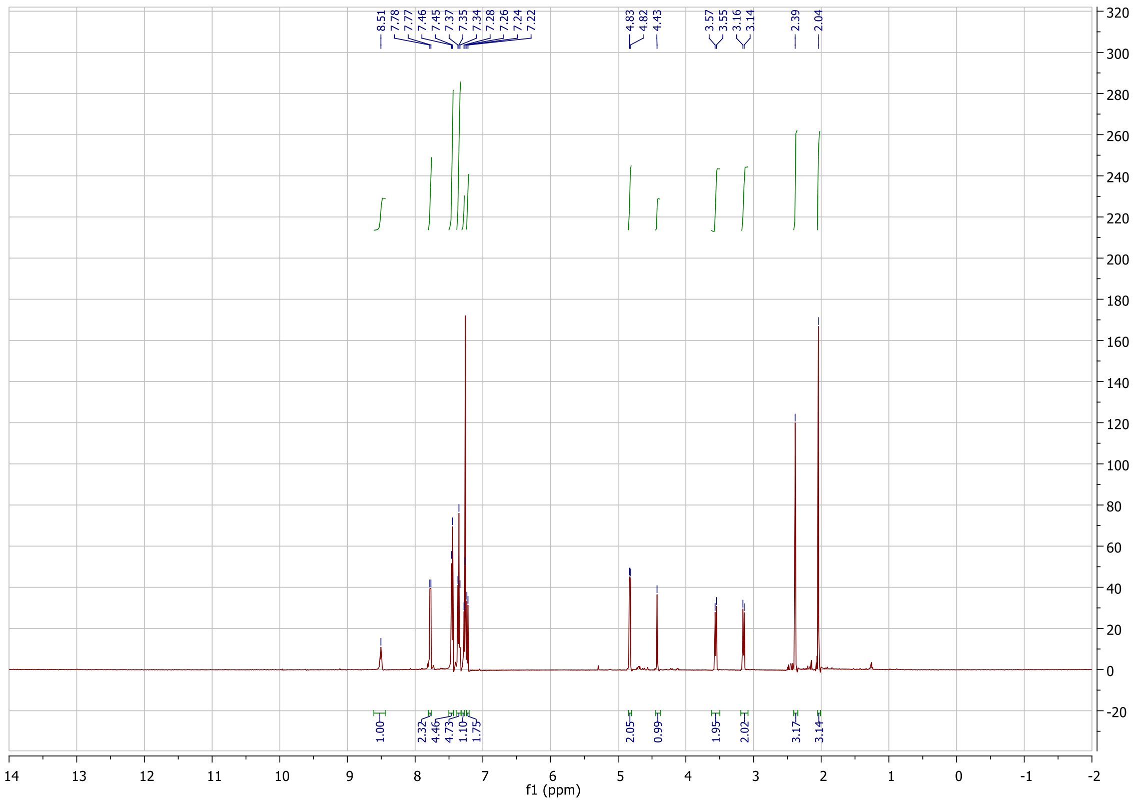

^{13}C NMR spectra of 1-benzhydryl-3-((tosylmethyl)carbamoyl)azetidin-3-yl acetate 4s

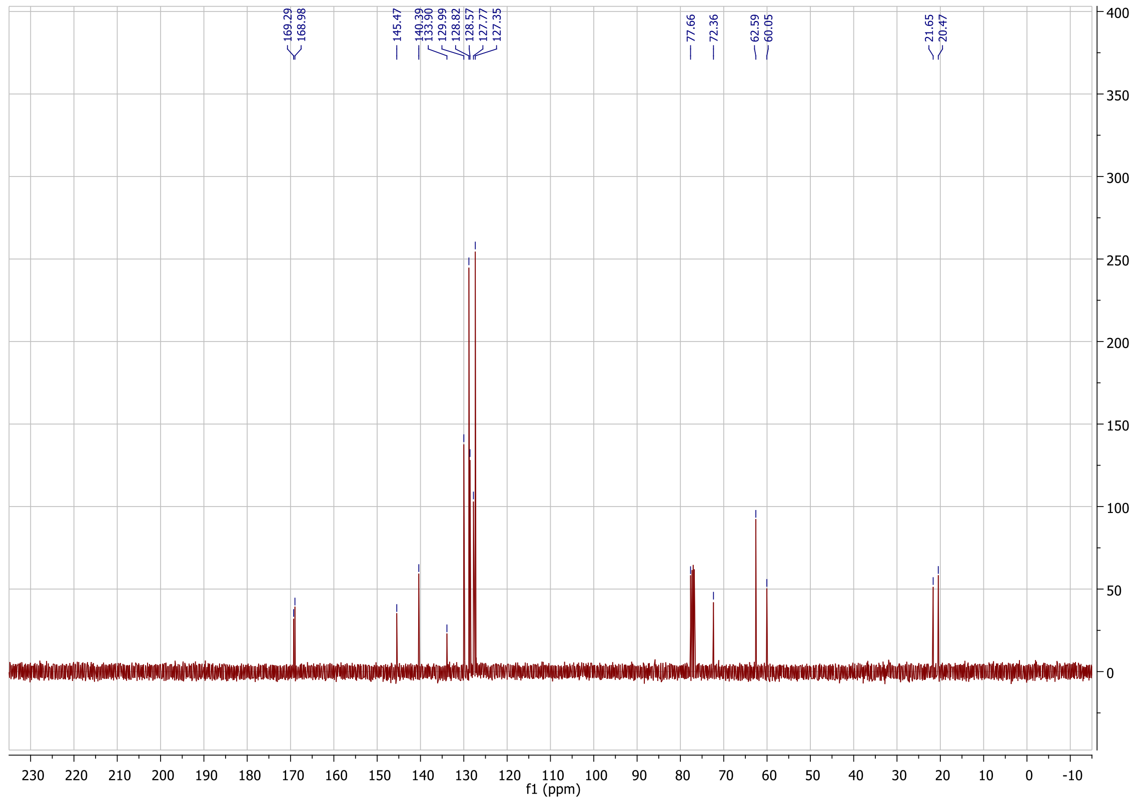

^{1}H NMR spectra of 1-benzhydryl-3-((tosylmethyl)carbamoyl)azetidin-3-yl pivalate 4t

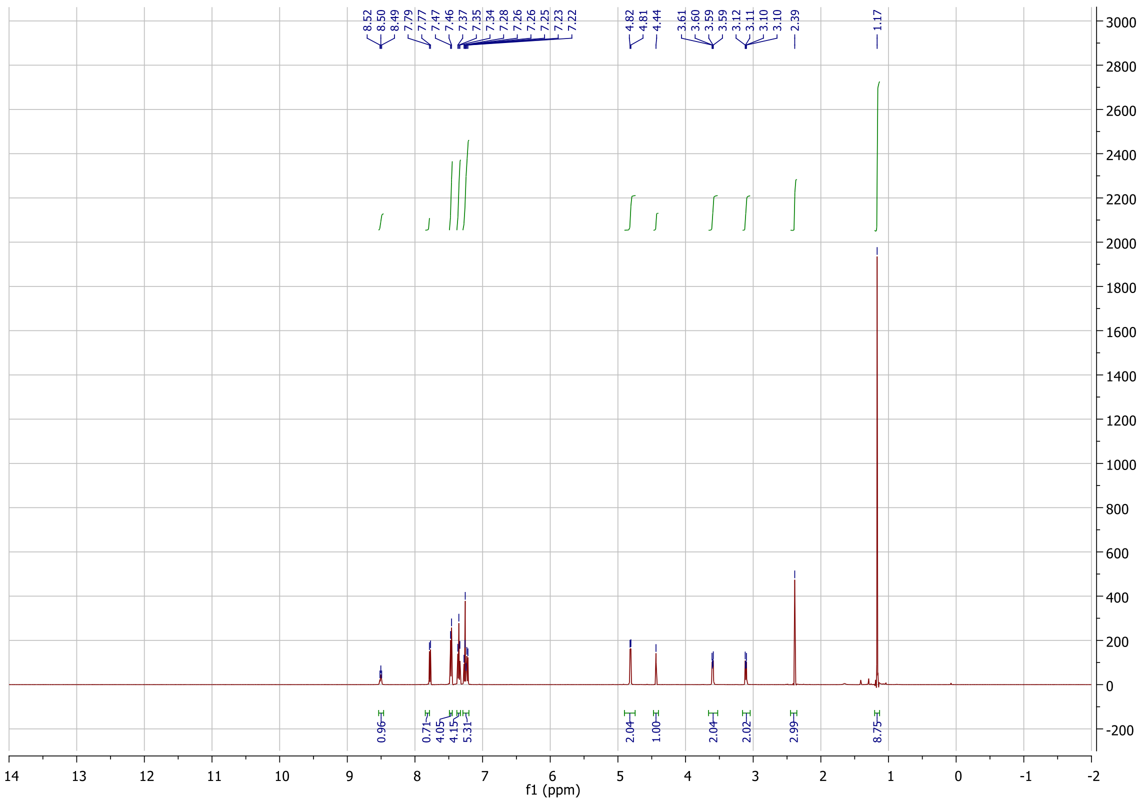

^{13}C NMR spectra of 1-benzhydryl-3-((tosylmethyl)carbamoyl)azetidin-3-yl pivalate 4t

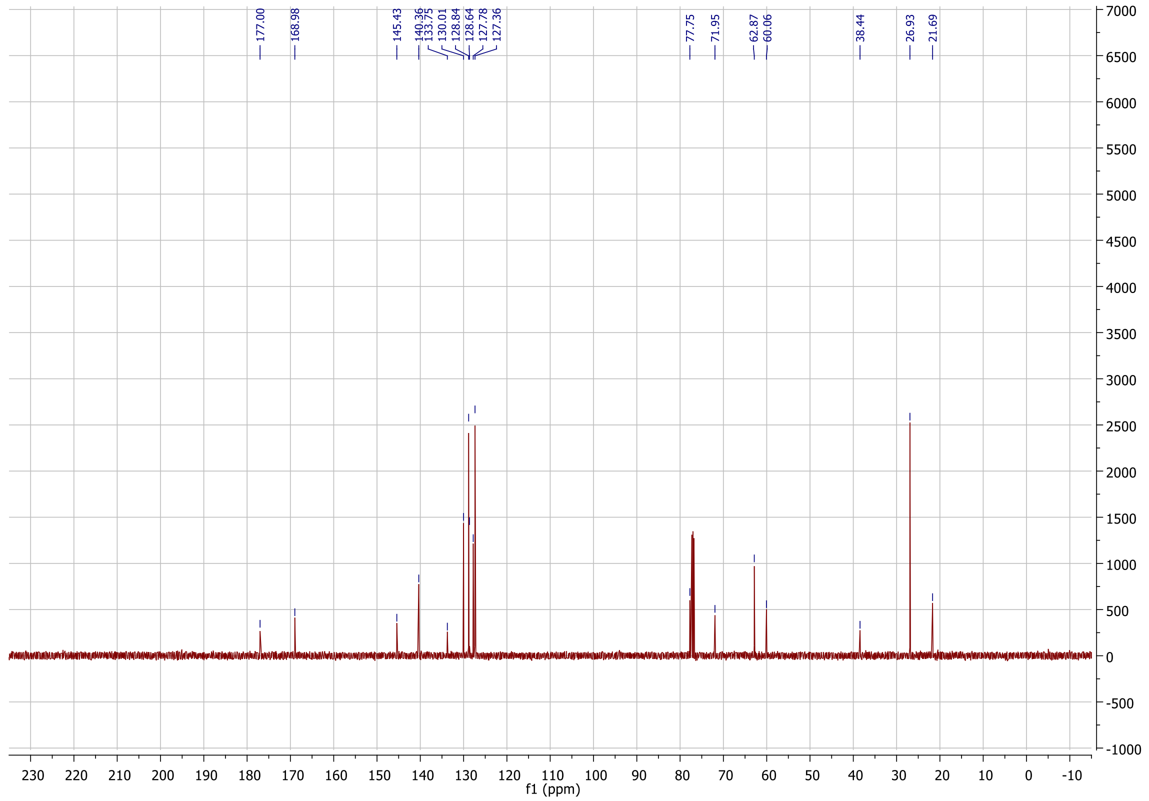

^{1}H NMR spectra of 1-benzhydryl-3-((tosylmethyl)carbamoyl)azetidin-3-yl 2-chloroacetate 4u

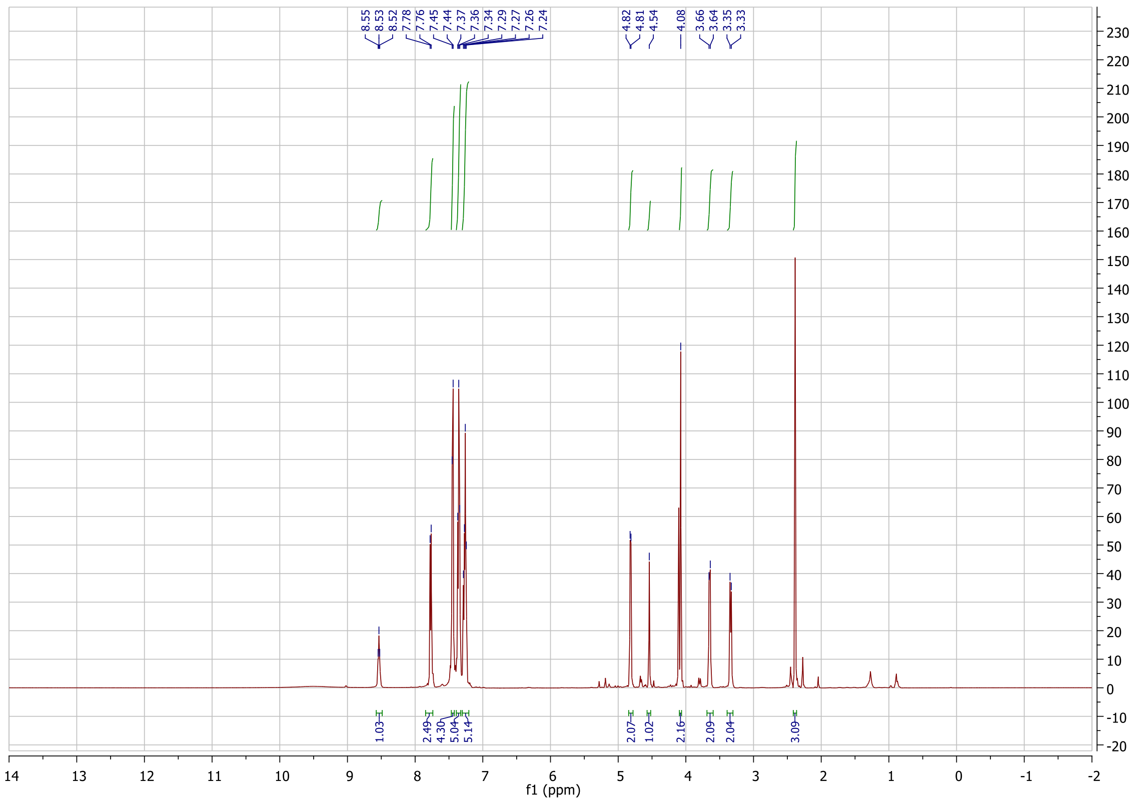

^{13}C NMR spectra of 1-benzhydryl-3-((tosylmethyl)carbamoyl)azetidin-3-yl 2-chloroacetate 4u

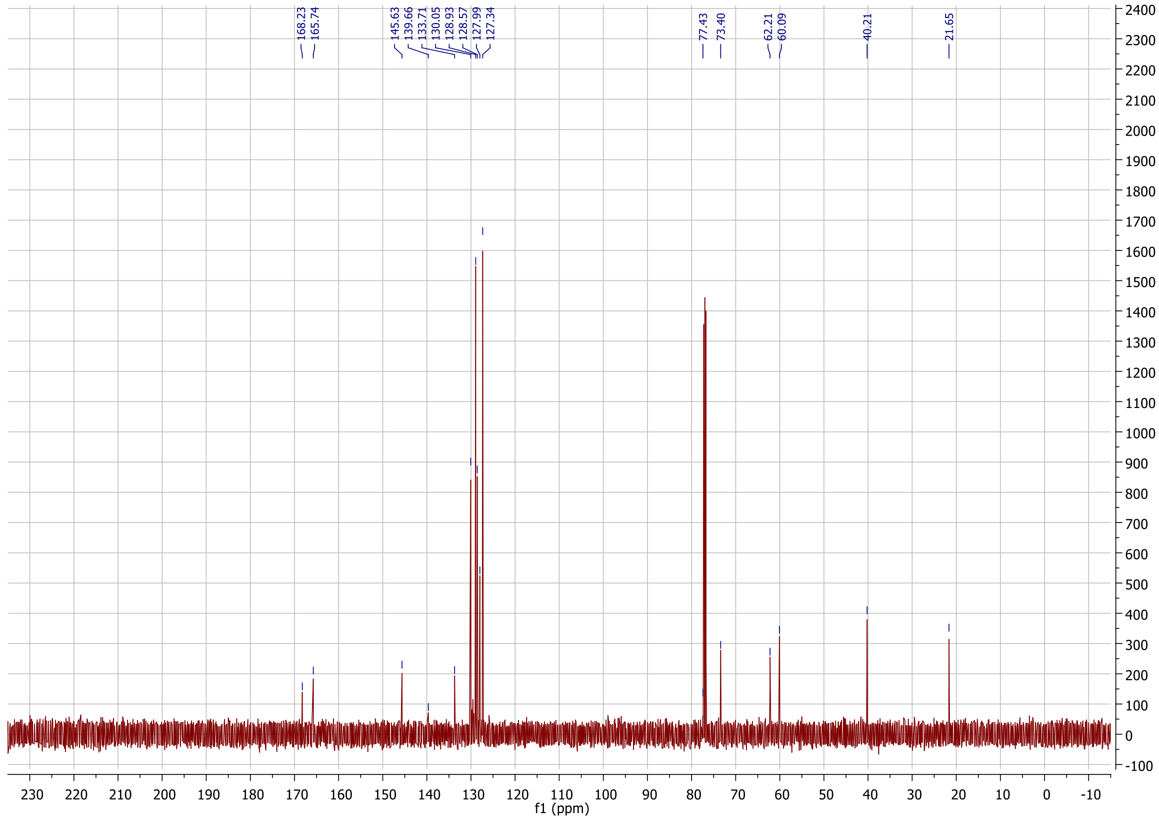

^{1}H NMR spectra of 1-benzhydryl-3-((tosylmethyl)carbamoyl)azetidin-3-yl methyl malonate 4v

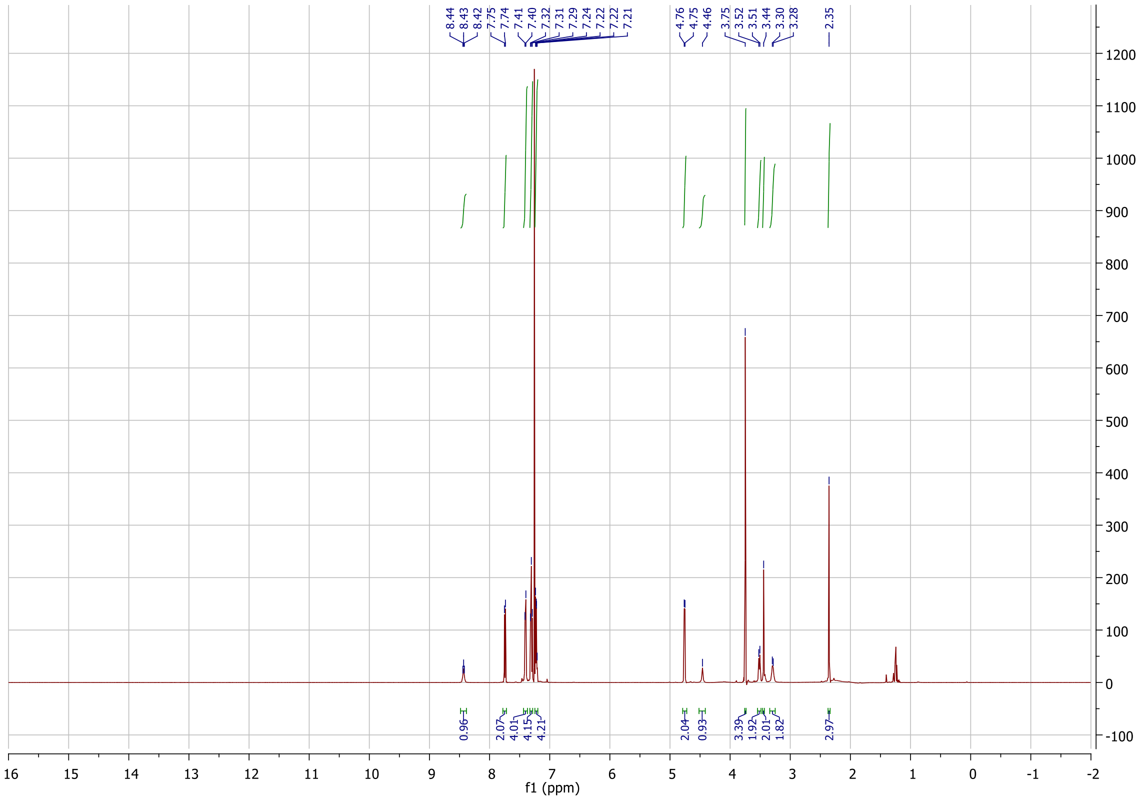

^{13}C NMR spectra of1-benzhydryl-3-((tosylmethyl)carbamoyl)azetidin-3-yl methyl malonate 4v

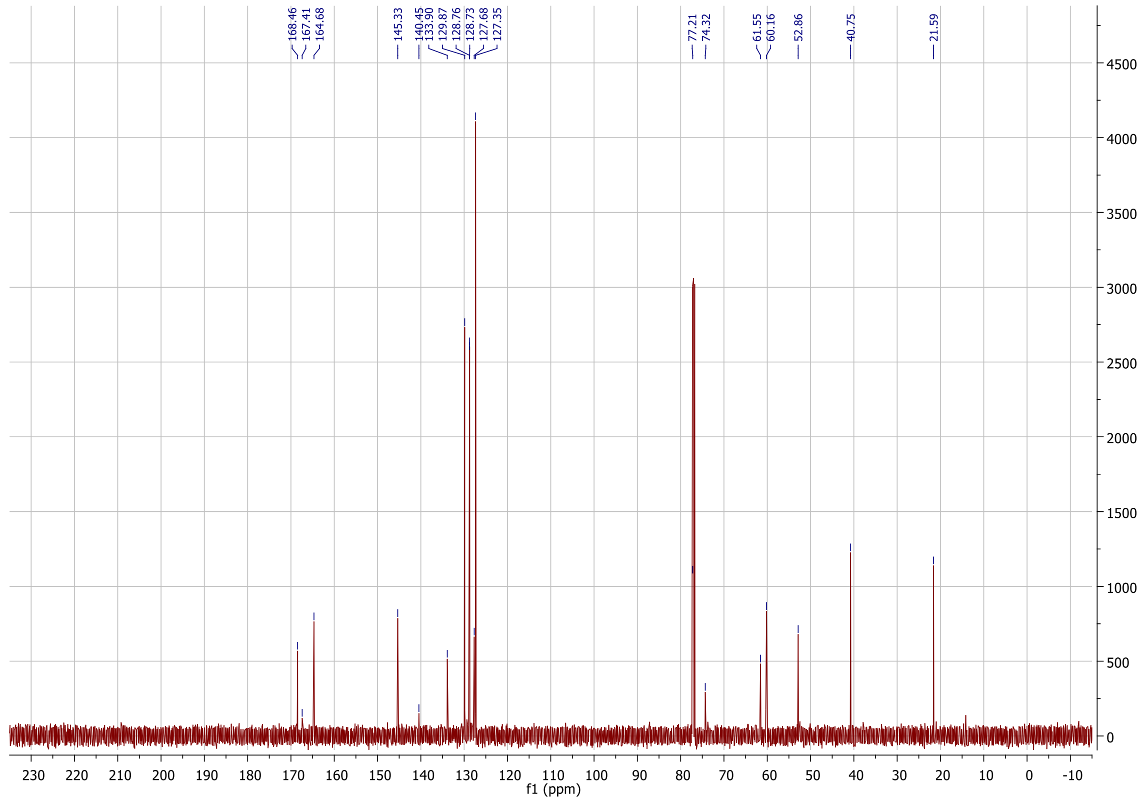

^1H NMR spectra of 1-benzhydryl-3-(4-nitrophenoxy)-N-(tosylmethyl)azetidine-3-carboxamide 5a

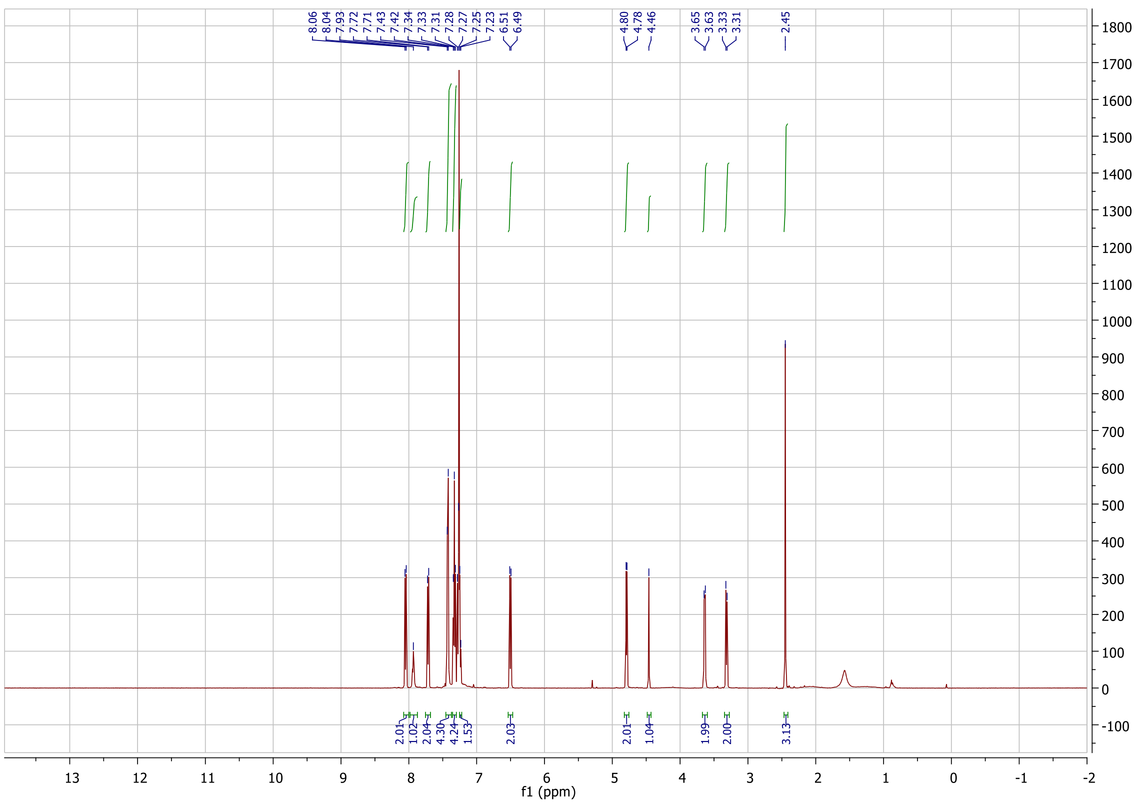

^{13}C NMR spectra of 1-benzhydryl-3-(4-nitrophenoxy)-N-(tosylmethyl)azetidine-3-carboxamide 5a

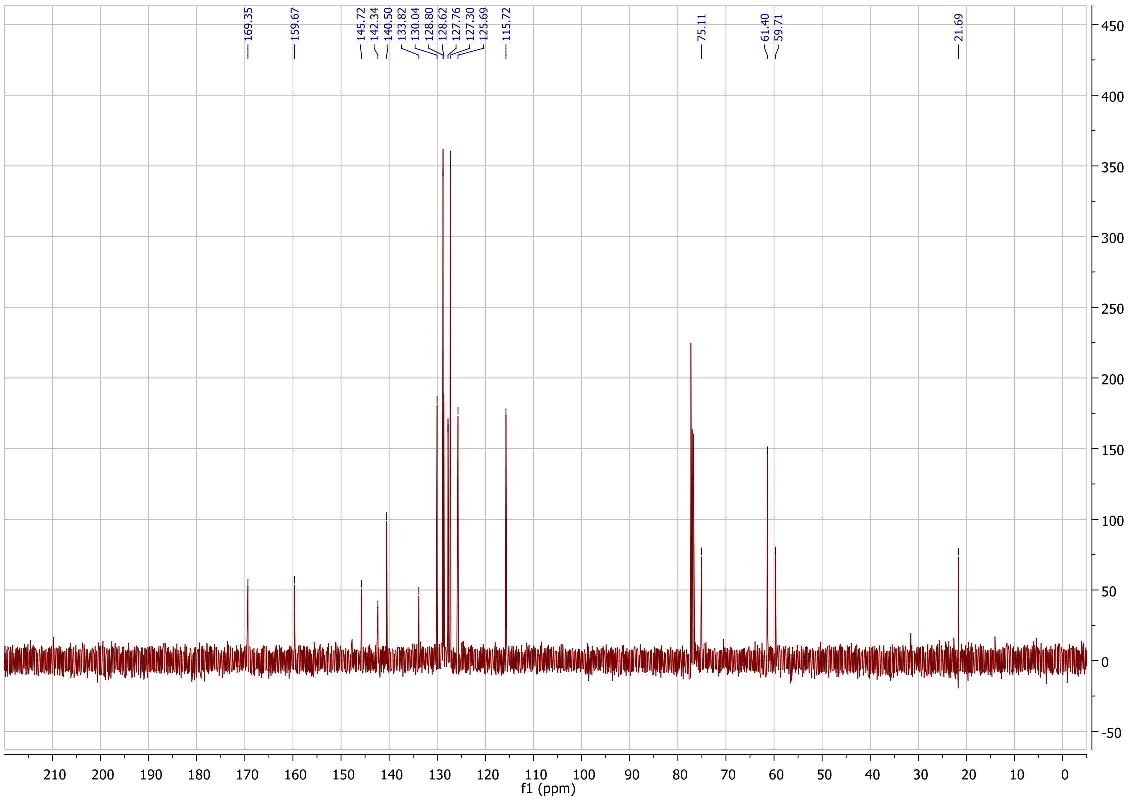

^{1}H NMR spectra of tert-butyl 3-(2,4-dinitrophenoxy)-3-((tosylmethyl)carbamoyl)azetidine-1-carboxylate 5b

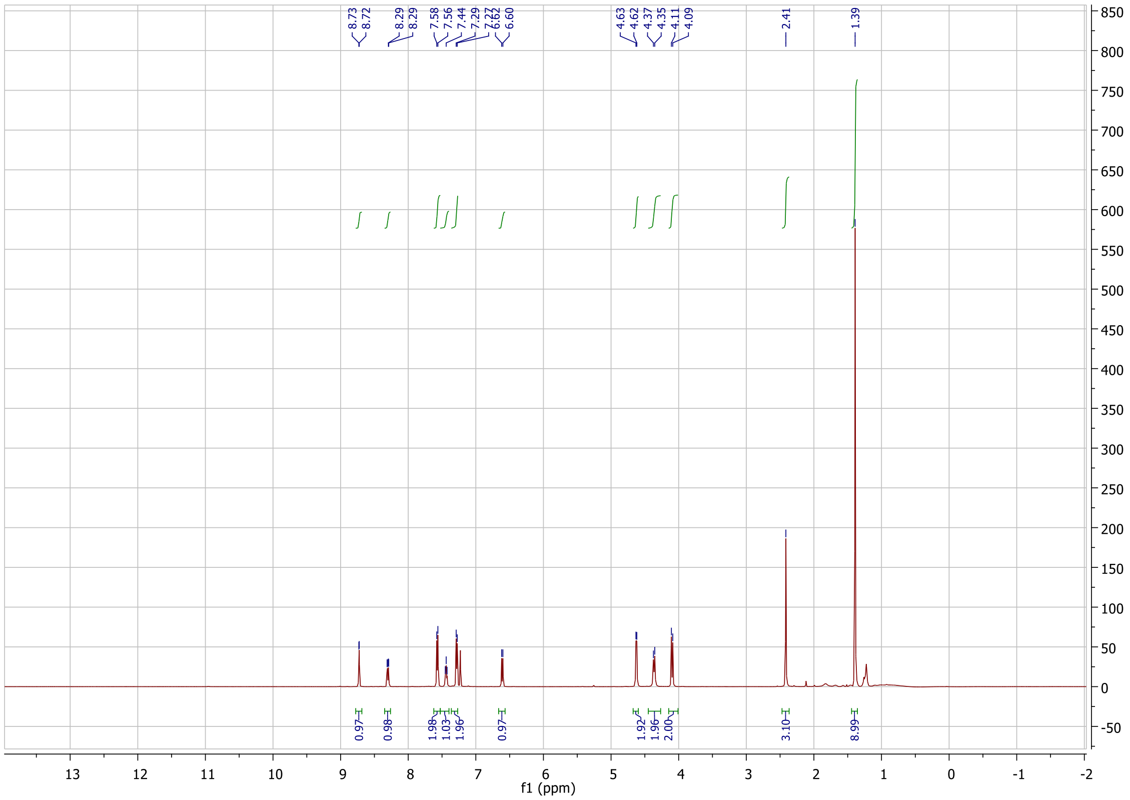

^{13}C NMR spectra of tert-butyl 3-(2,4-dinitrophenoxy)-3-((tosylmethyl)carbamoyl)azetidine-1-carboxylate 5b

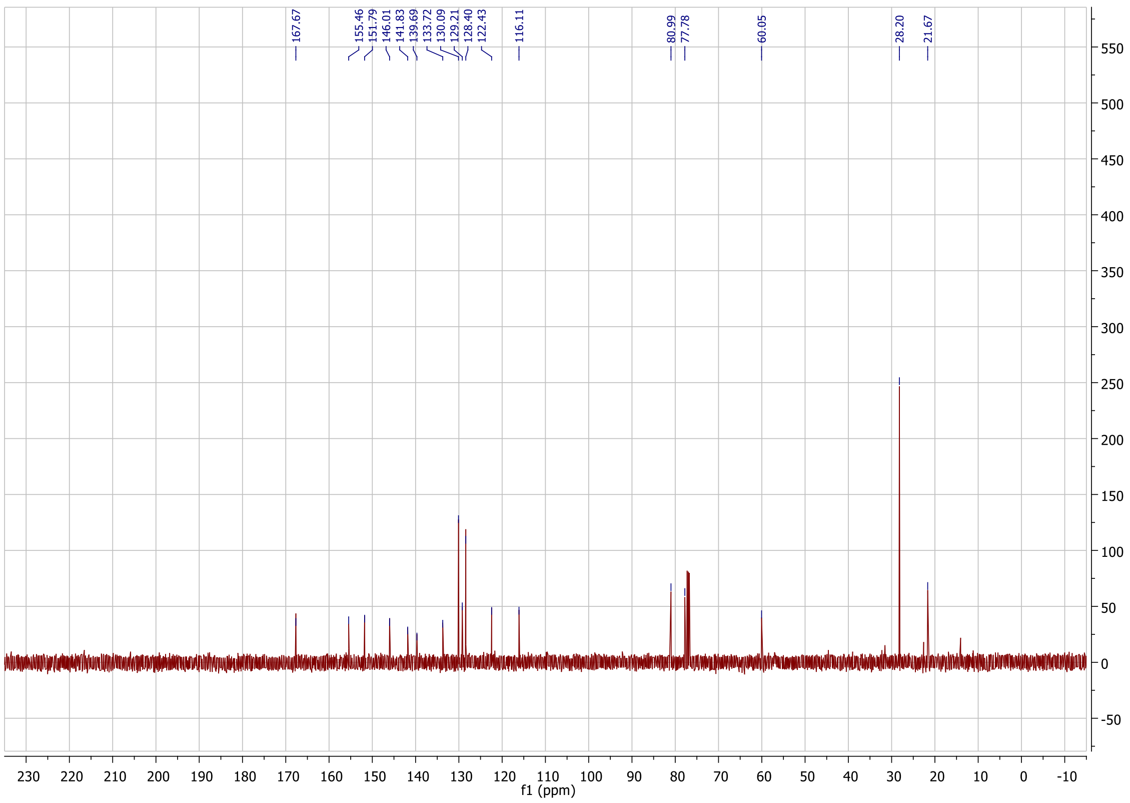

^{1}H NMR spectra of 1-benzhydryl-3-(N-butylbenzamido)-N-(tosylmethyl)azetidine-3-carboxamide 6a

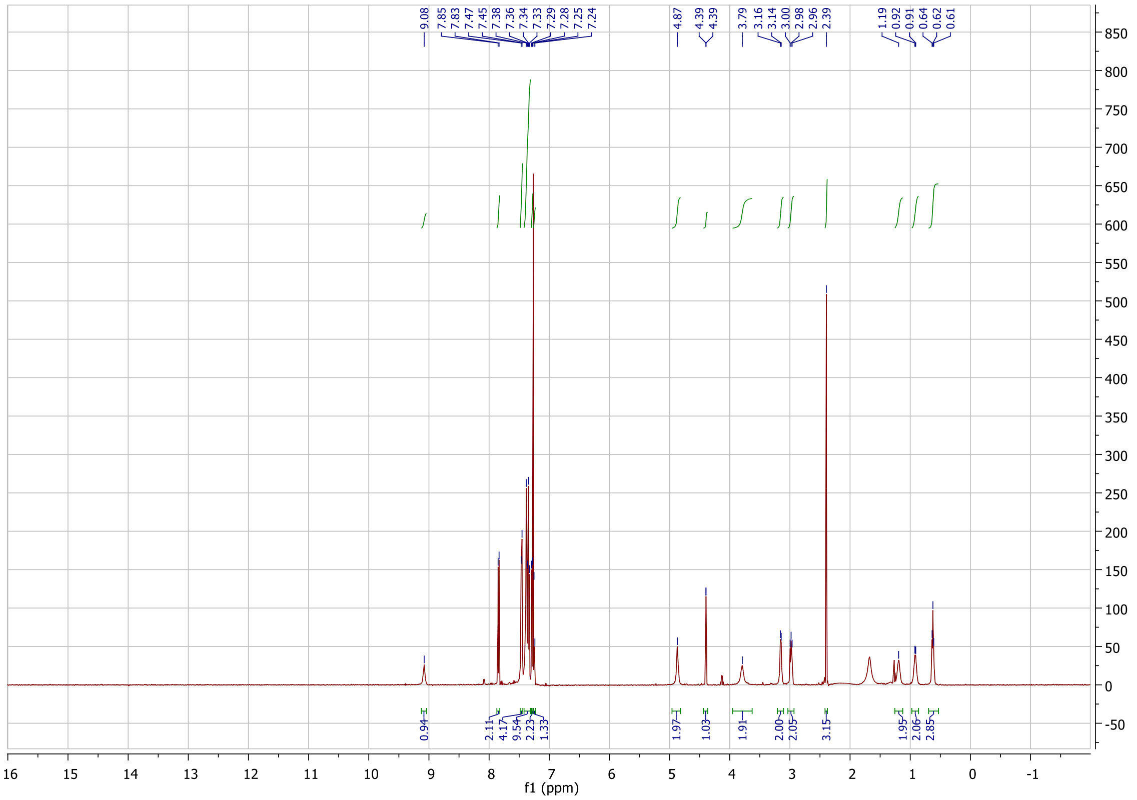

^{13}C NMR spectra of 1-benzhydryl-3-(N-butylbenzamido)-N-(tosylmethyl)azetidine-3-carboxamide 6a

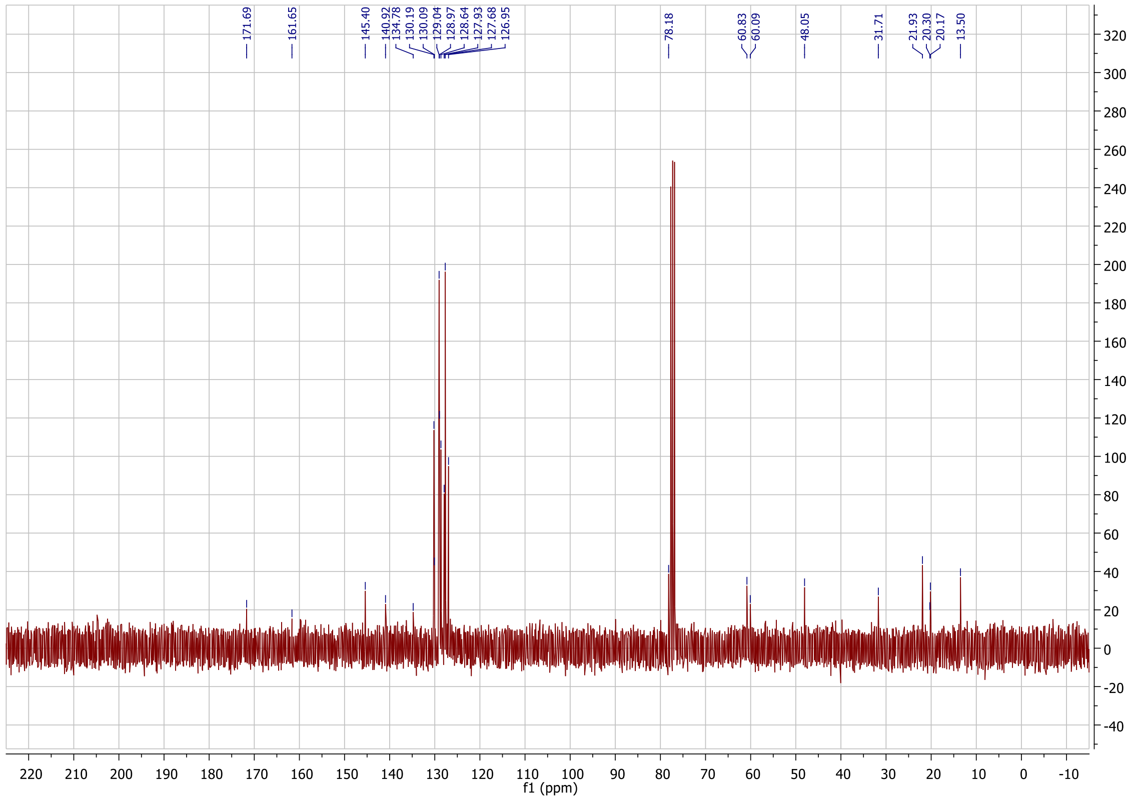

^{1}H NMR spectra of tert-butyl 3-(N-butylbenzamido)-3-((tosylmethyl)carbamoyl)azetidine-1-carboxylate 6b

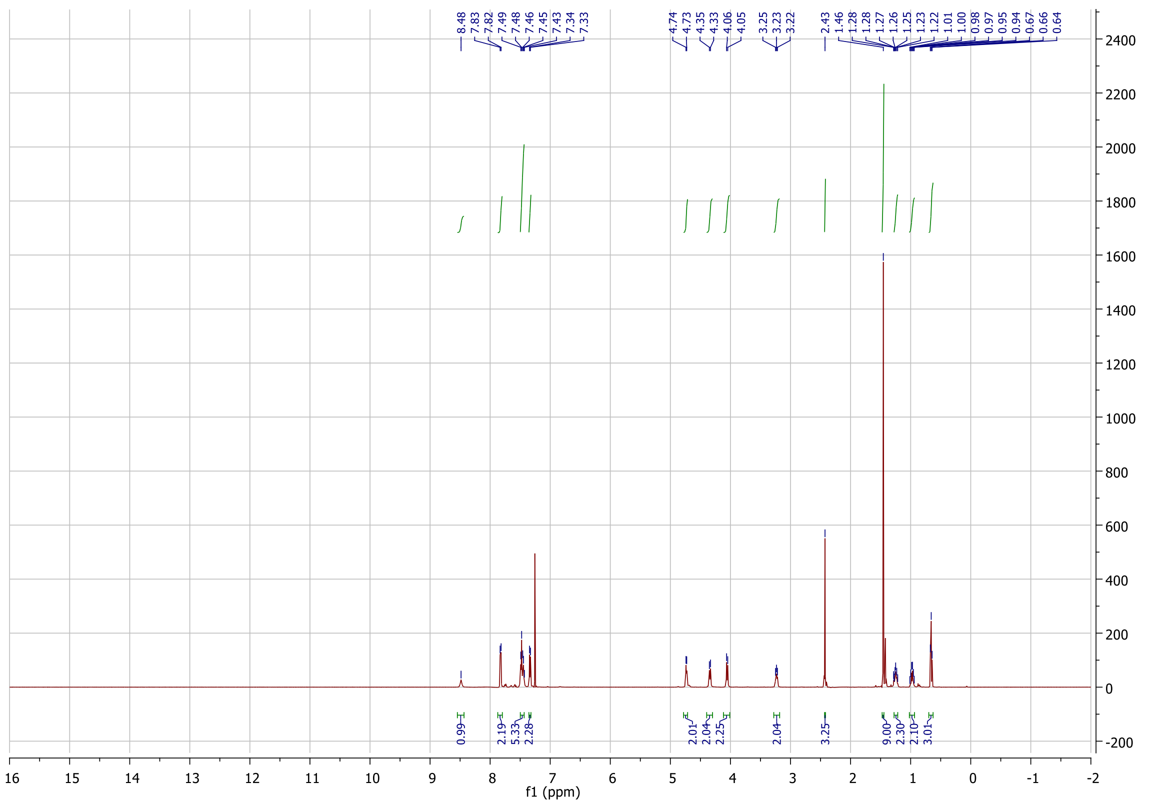

^{13}C NMR spectra of tert-butyl 3-(N-butylbenzamido)-3-((tosylmethyl)carbamoyl)azetidine-1-carboxylate 6b

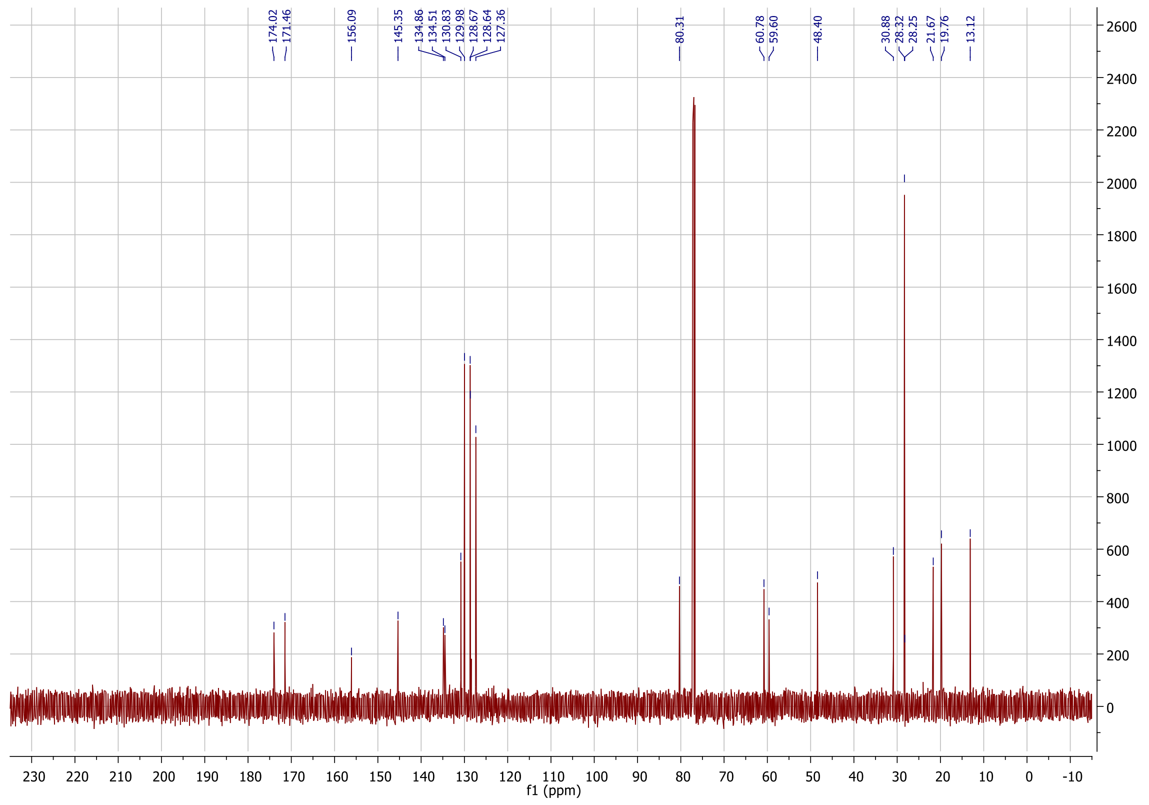

^{1}H NMR spectra of 3-(N-benzyl-2-chlorobenzamido)-N-(tosylmethyl)oxetane-3-carboxamide 6c

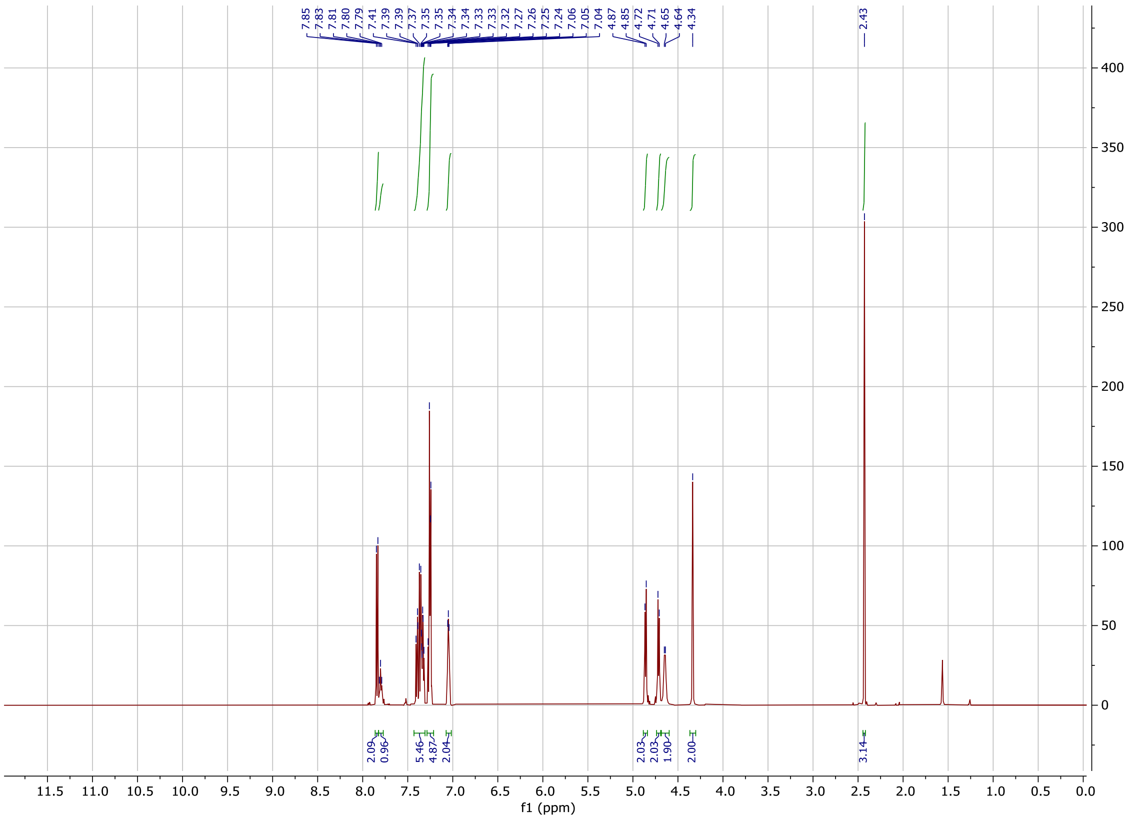

^{13}C NMR spectra of 3-(N-benzyl-2-chlorobenzamido)-N-(tosylmethyl)oxetane-3-carboxamide 6c

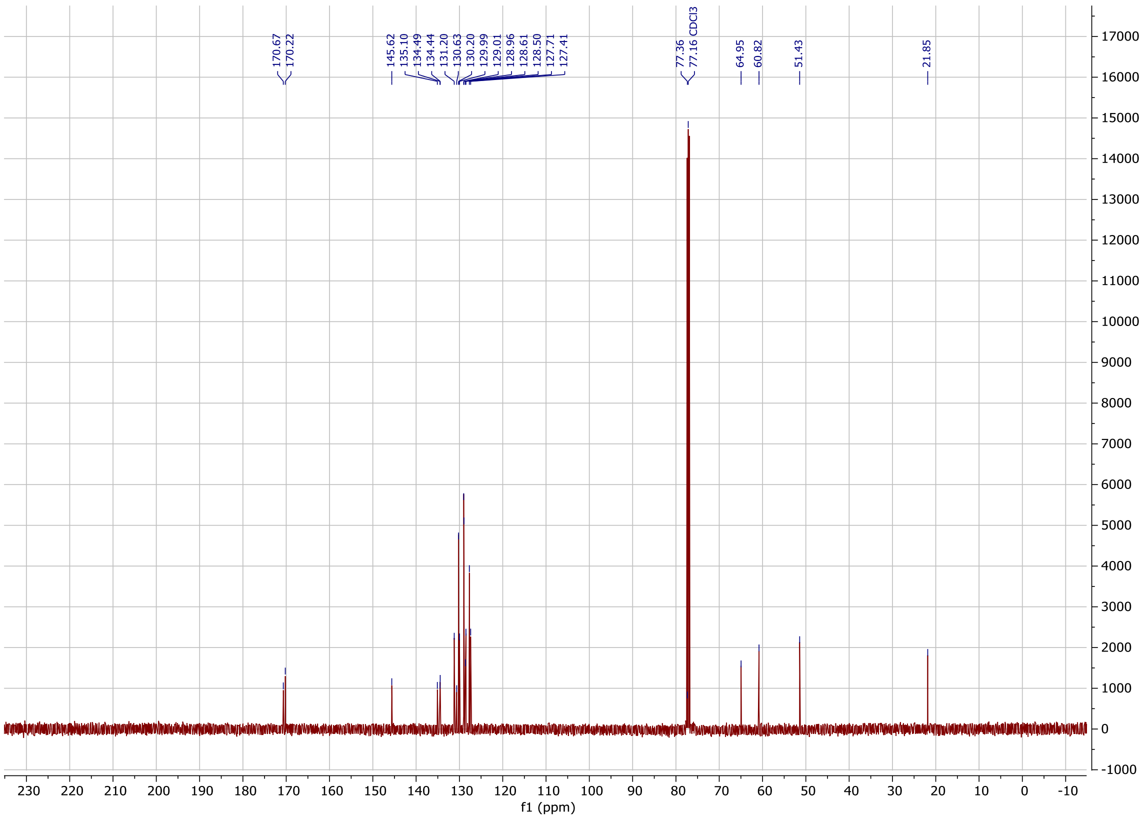

^{1}H NMR spectra of ethyl 2-(1-benzhydryl-3-(N-butylbenzamido)azetidine-3-carboxamido)acetate 6d

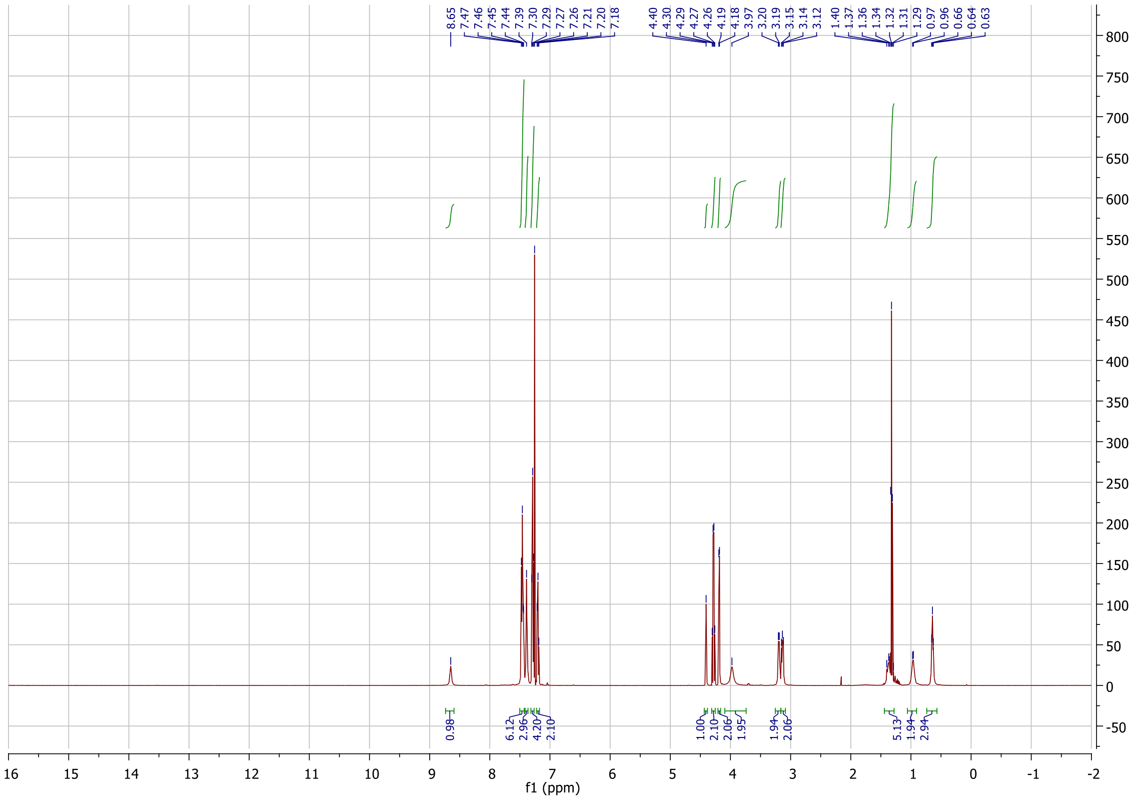

^{13}C NMR spectra of ethyl 2-(1-benzhydryl-3-(N-butylbenzamido)azetidine-3-carboxamido)acetate 6d

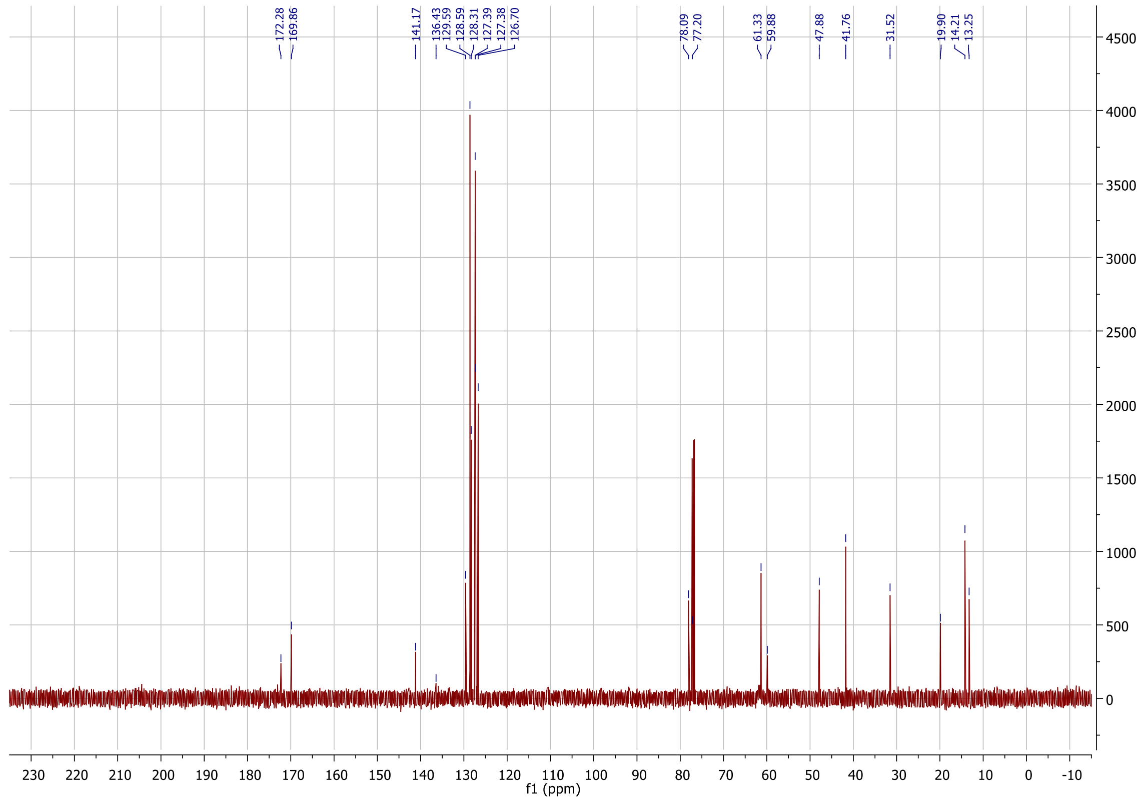

^{1}H NMR spectra of 1-benzhydryl-3-(N-butyl-2-chlorobenzamido)-N-(tosylmethyl)azetidine-3-carboxamide 6e

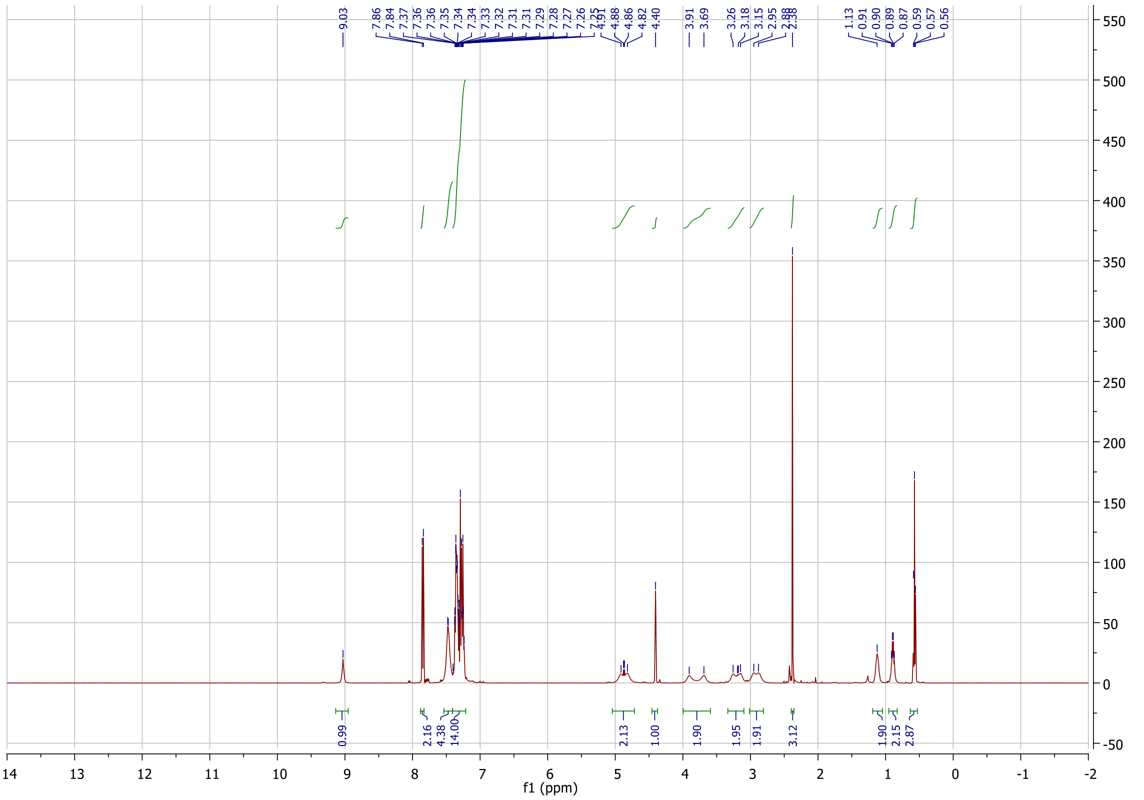

^{13}C NMR spectra of 1-benzhydryl-3-(N-butyl-2-chlorobenzamido)-N-(tosylmethyl)azetidine-3-carboxamide 6e

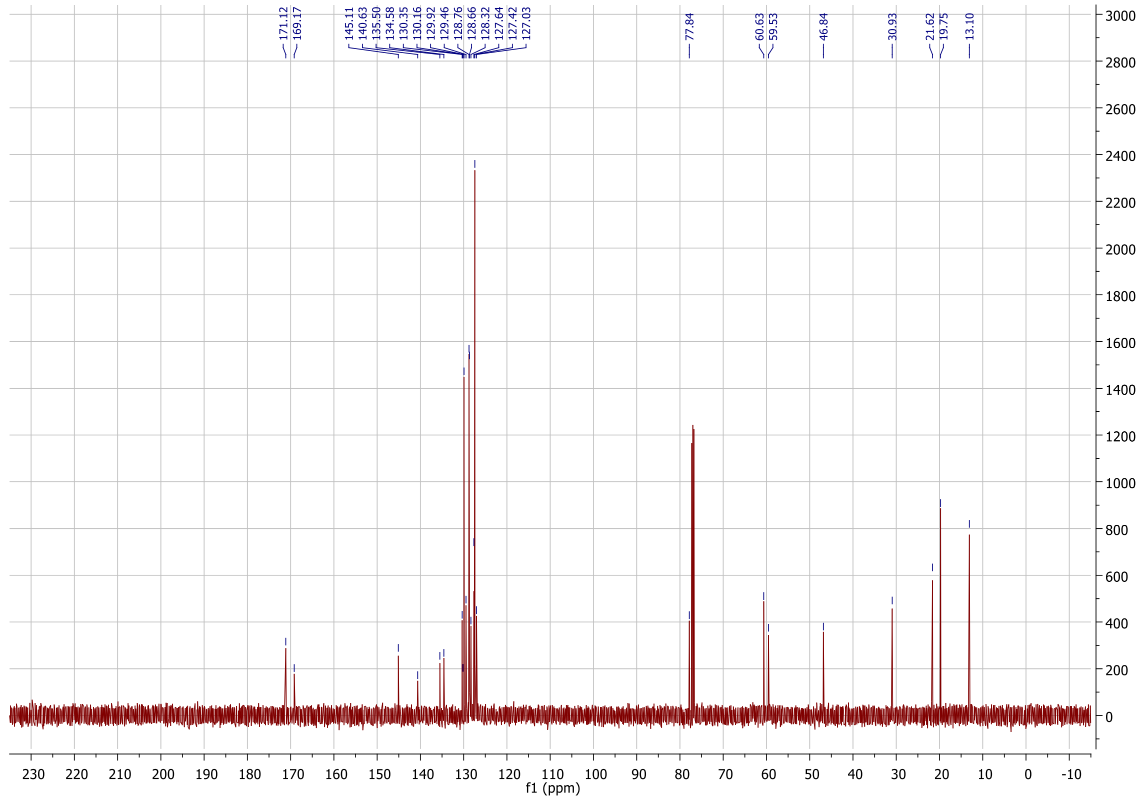

^{1}H NMR spectra of 1-benzhydryl-3-(N-butylcinnamamido)-N-(tosylmethyl)azetidine-3-carboxamide 6f

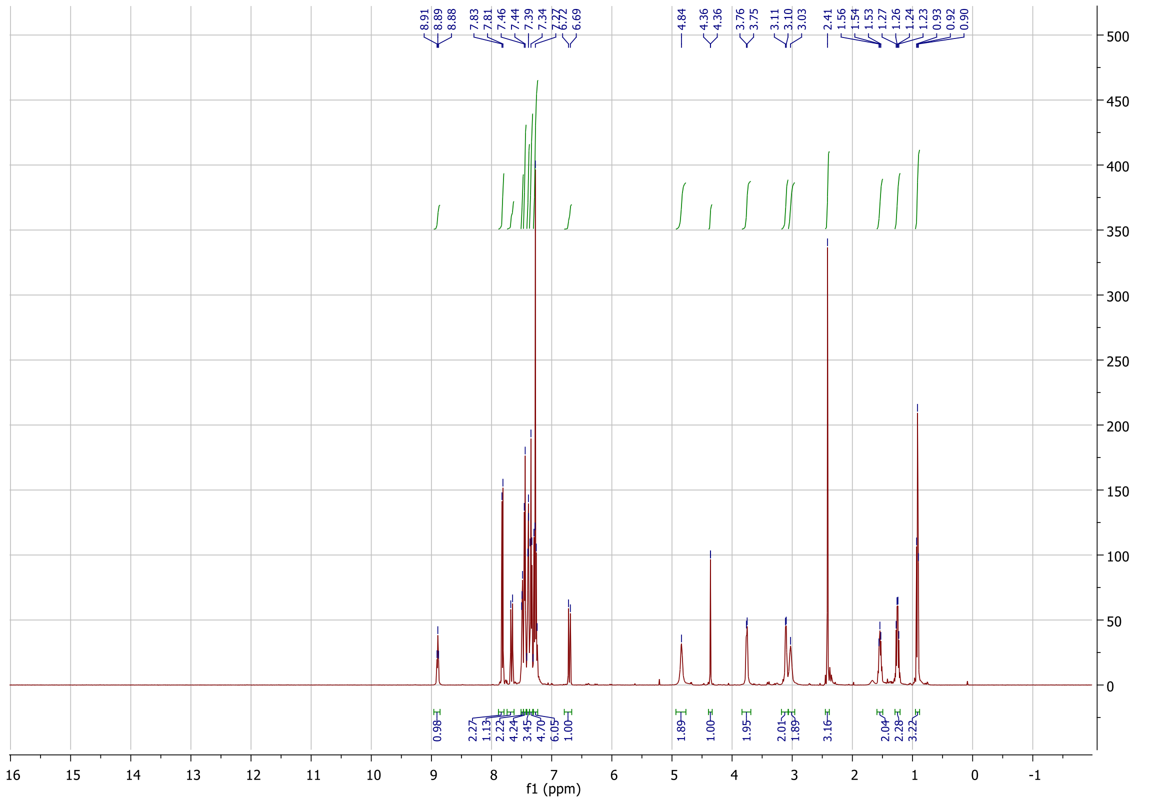

^{13}C NMR spectra of 1-benzhydryl-3-(N-butylcinnamamido)-N-(tosylmethyl)azetidine-3-carboxamide 6f

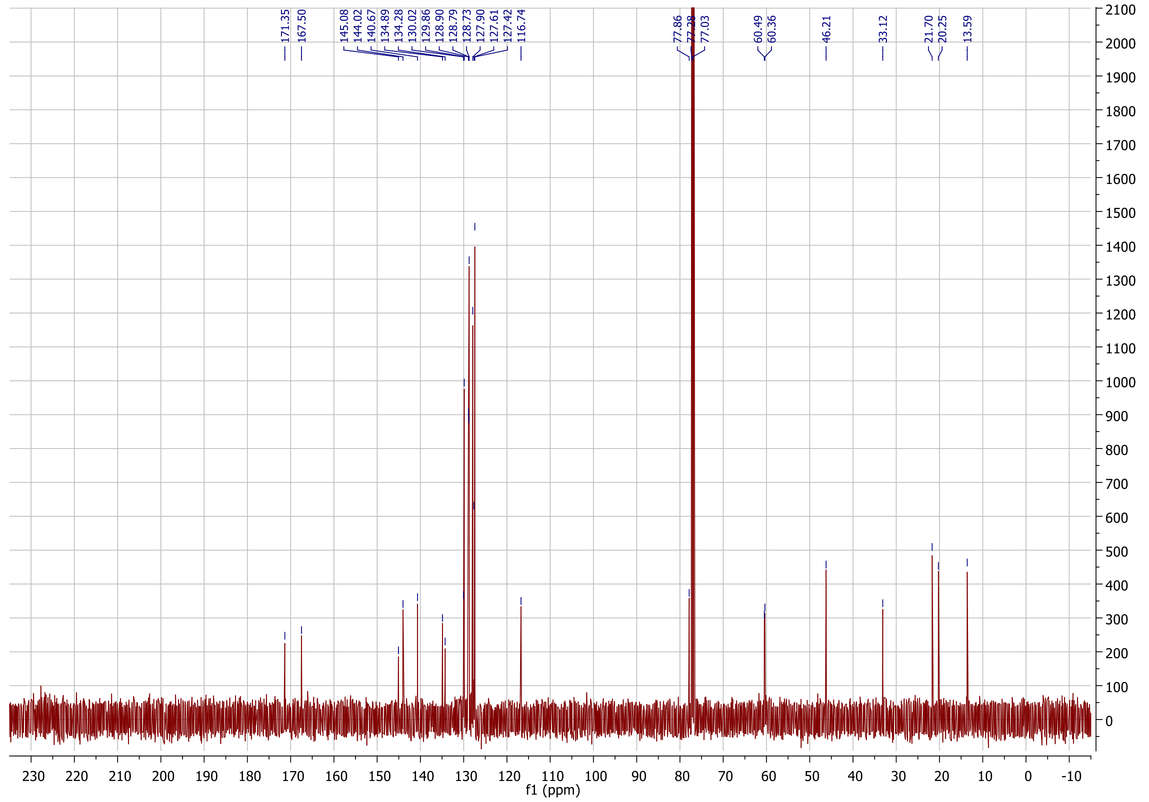

^{1}H NMR spectra of 1-benzhydryl-3-(N-benzylbenzamido)-N-(tosylmethyl)azetidine-3-carboxamide 6g

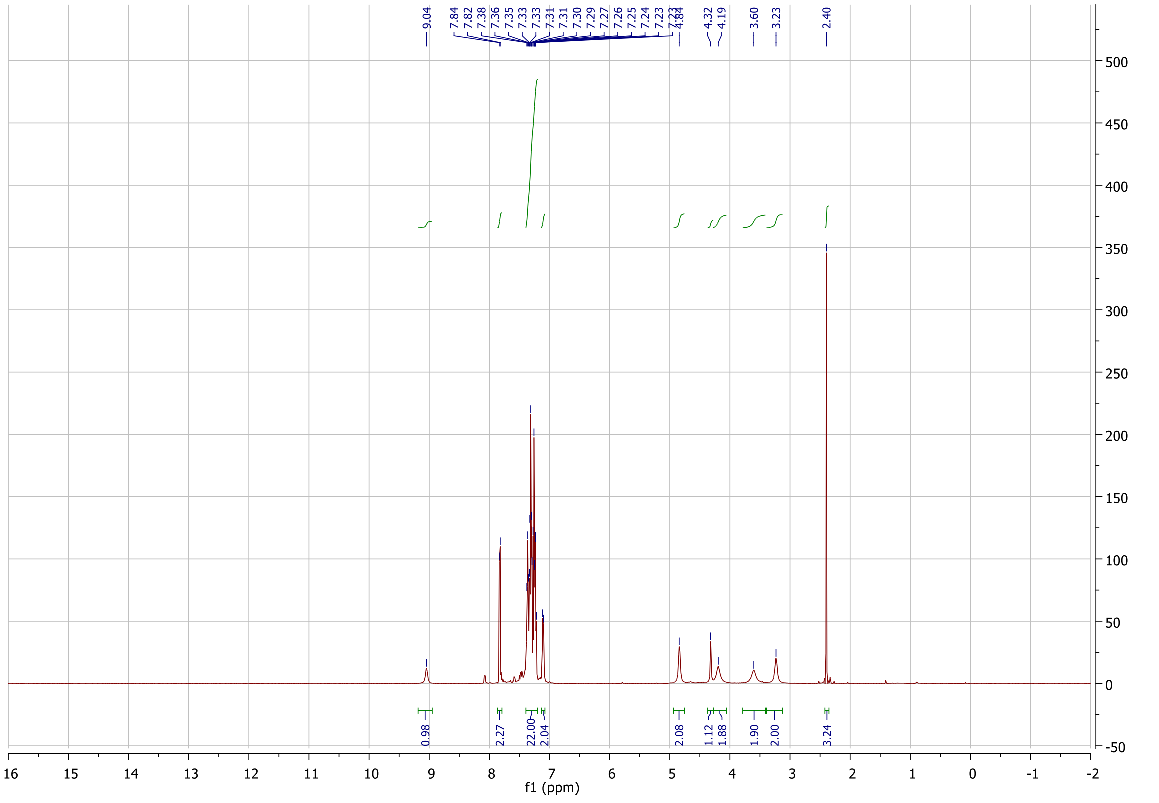

^{13}C NMR spectra of 1-benzhydryl-3-(N-benzylbenzamido)-N-(tosylmethyl)azetidine-3-carboxamide 6g

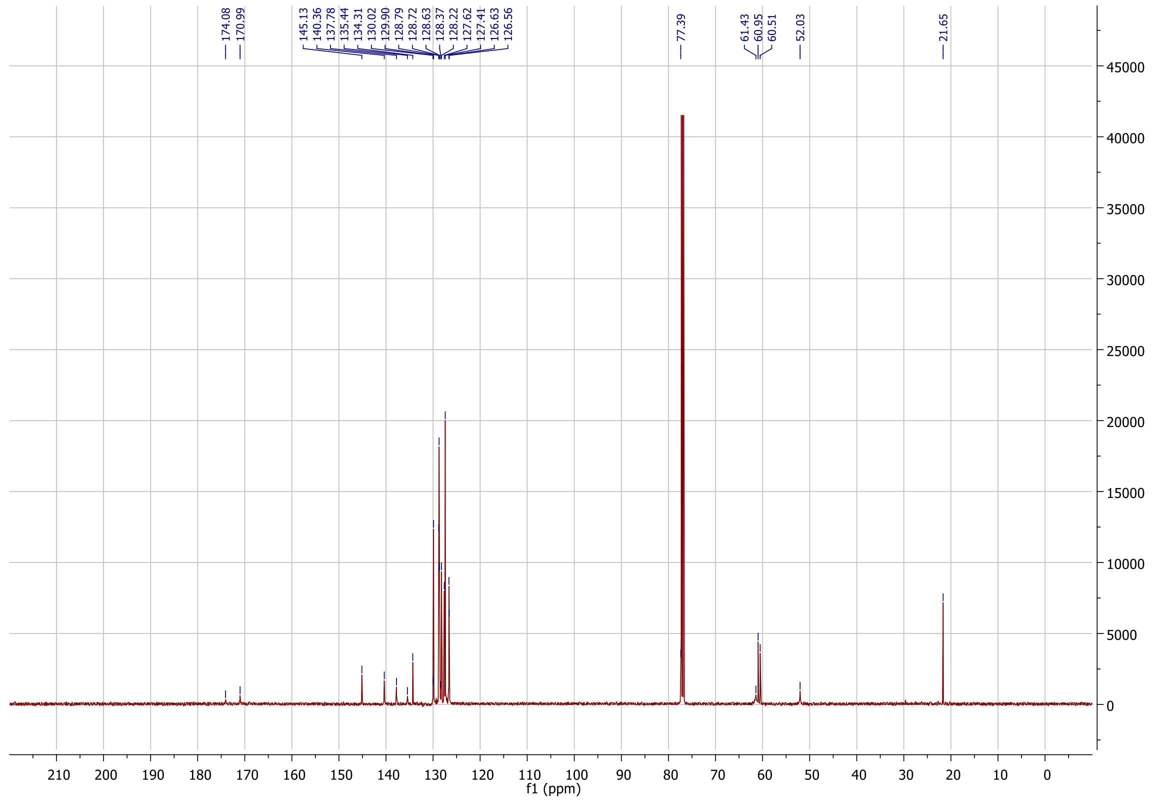

^{1}H NMR spectra of tert-butyl 3-(N-benzyl-2-chlorobenzamido)-3-((tosylmethyl)carbamoyl)azetidine-1-carboxylate 6h

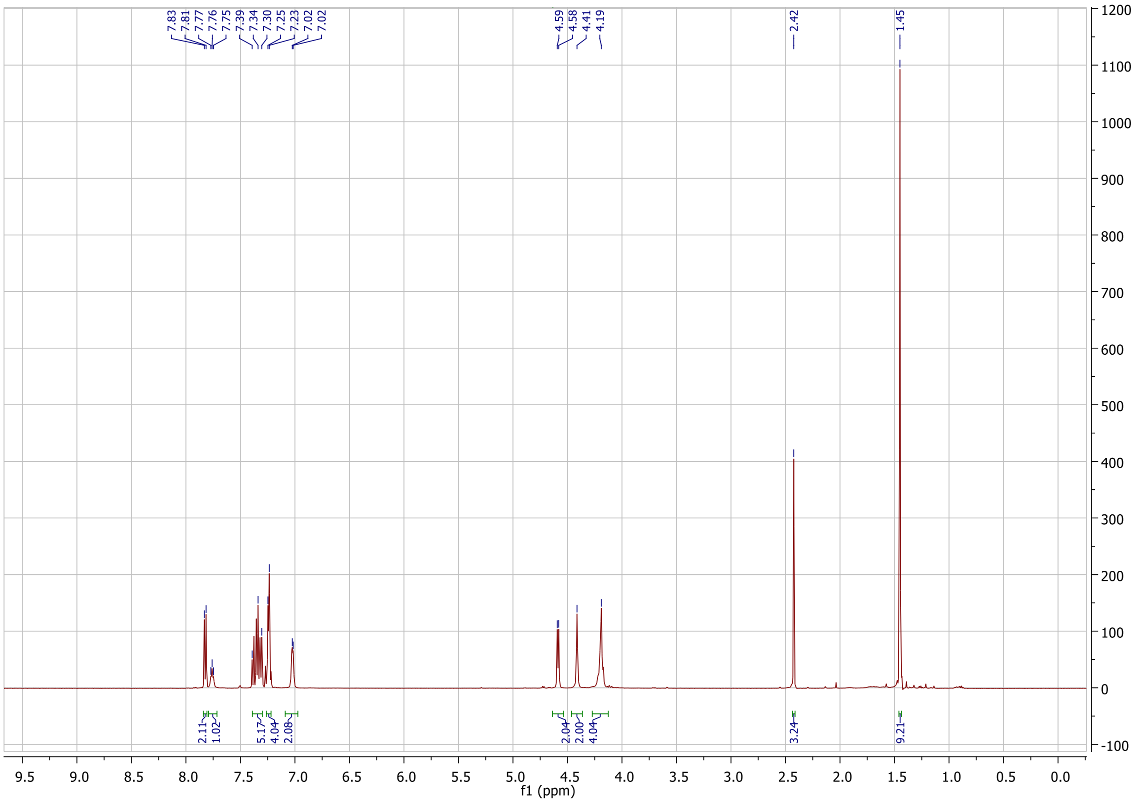

^{13}C NMR spectra of tert-butyl 3-(N-benzyl-2-chlorobenzamido)-3-((tosylmethyl)carbamoyl)azetidine-1-carboxylate 6h

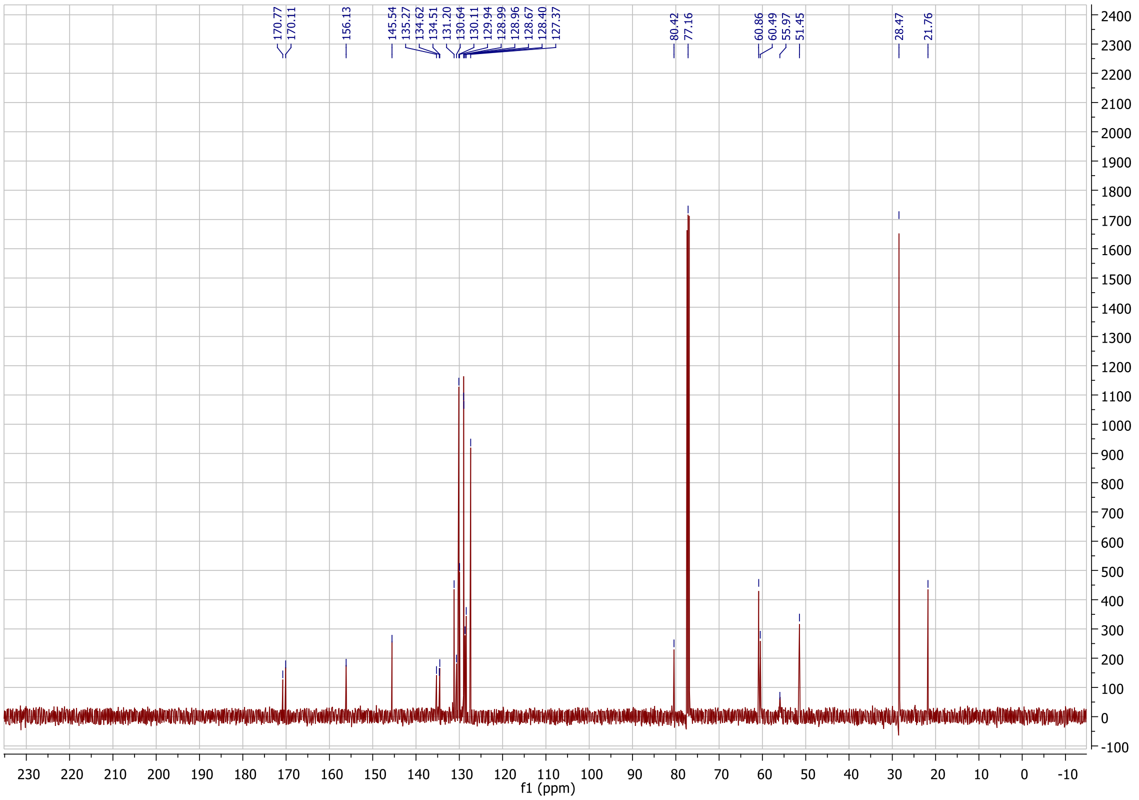

^{1}H NMR spectra of Tert-butyl 3-(N-(3,5-bis(trifluoromethyl)benzyl)-2-chlorobenzamido)-3-((tosylmethyl)carbamoyl)azetidine-1-carboxylate 6i

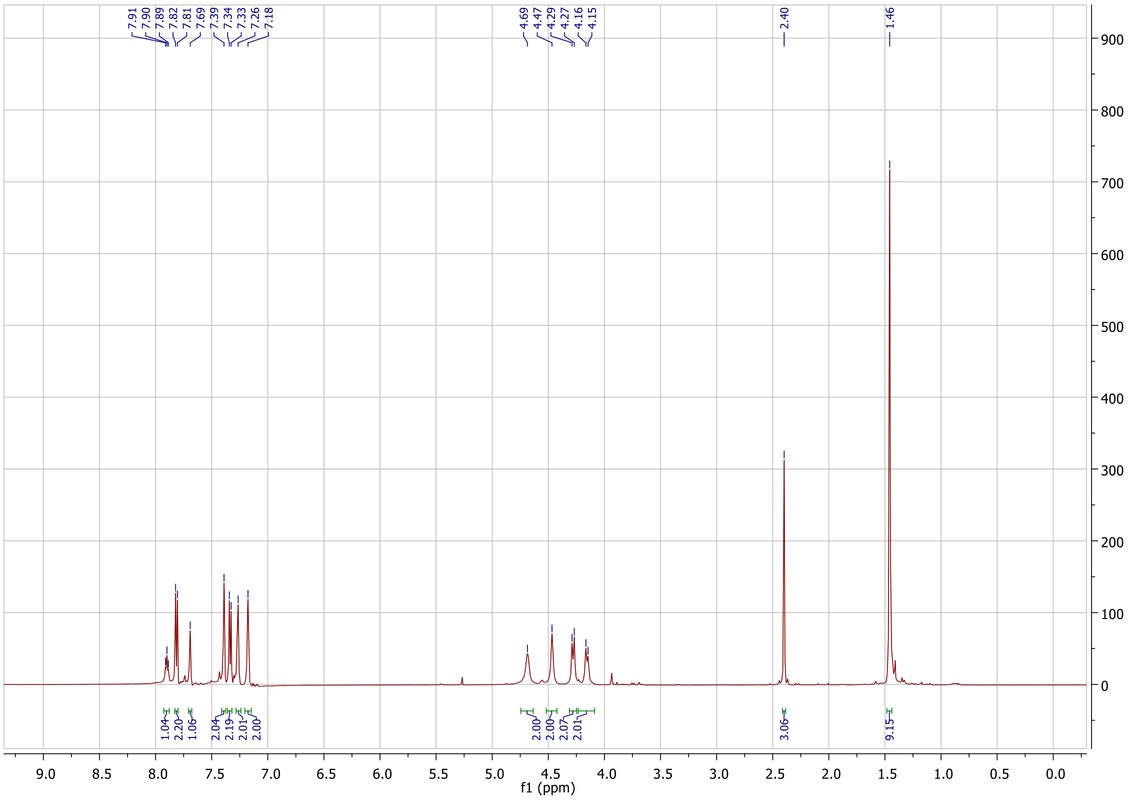

^{13}C NMR spectra of Tert-butyl 3-(N-(3,5-bis(trifluoromethyl)benzyl)-2-chlorobenzamido)-3-((tosylmethyl)carbamoyl)azetidine-1-carboxylate 6i

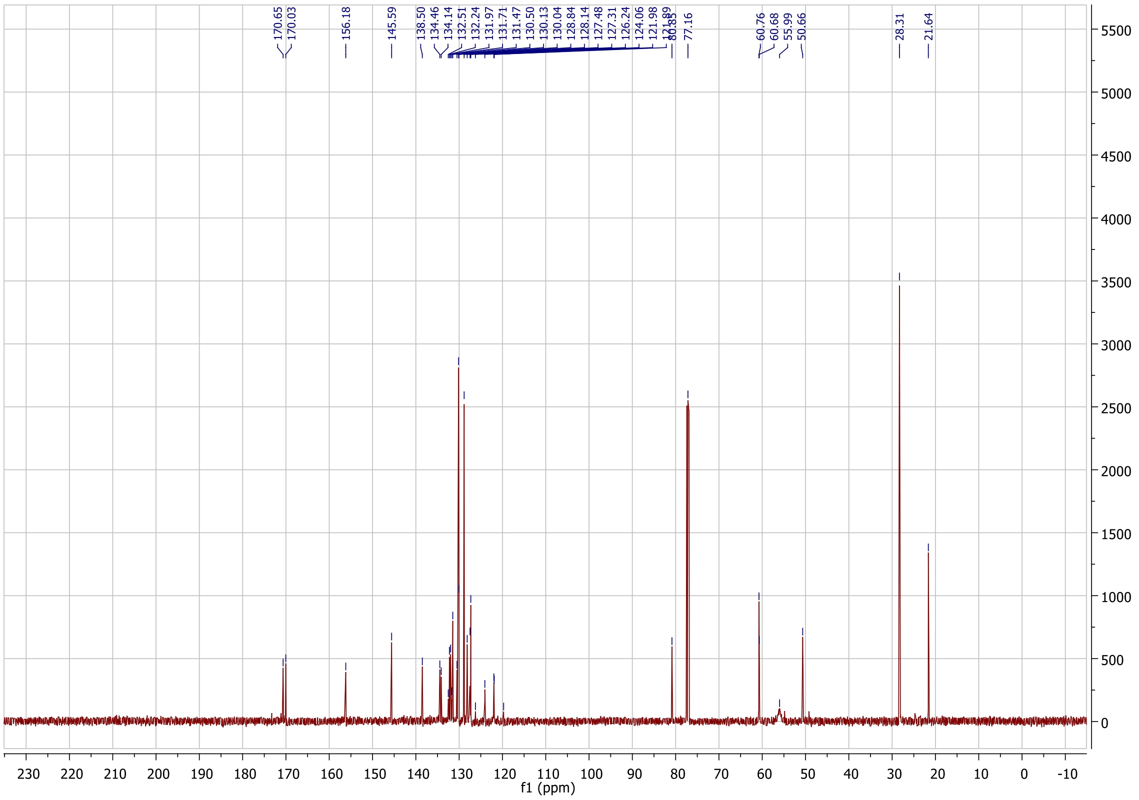

^{1}H NMR spectra of Tert-butyl 3-(2-chloro-N-(thiophen-2-ylmethyl)benzamido)-3-((tosylmethyl)carbamoyl)azetidine-1-carboxylate 6j

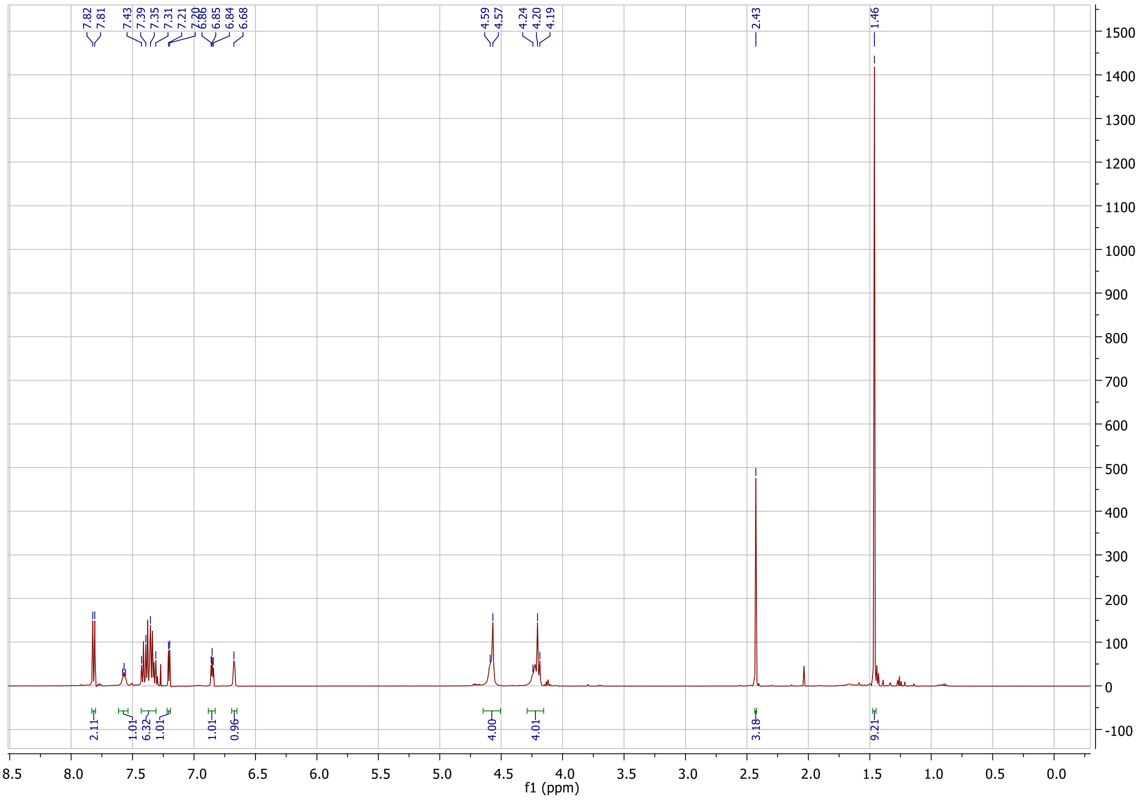

^{13}C NMR spectra of Tert-butyl 3-(2-chloro-N-(thiophen-2-ylmethyl)benzamido)-3-((tosylmethyl)carbamoyl)azetidine-1-carboxylate 6j

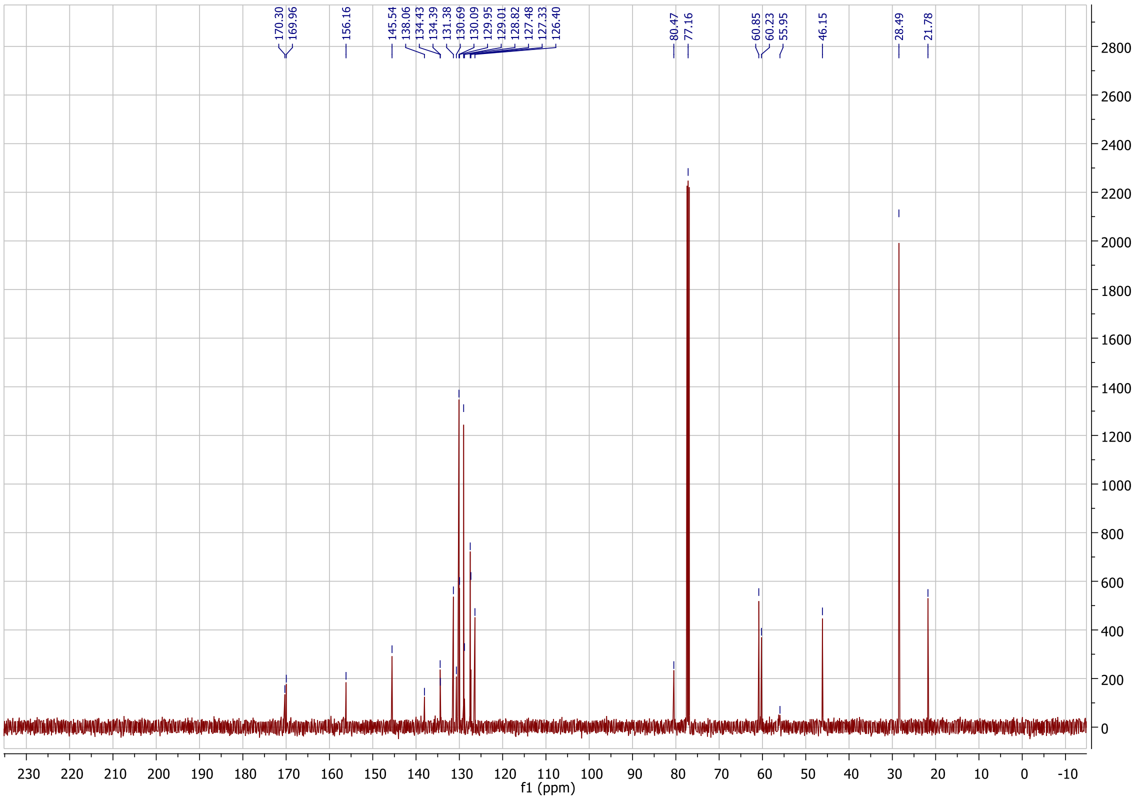

^{1}H NMR spectra of Tert-butyl 3-(N-benzyl-2-chlorobenzamido)-3-(tert-butylcarbamoyl)azetidine-1-carboxylate 6k

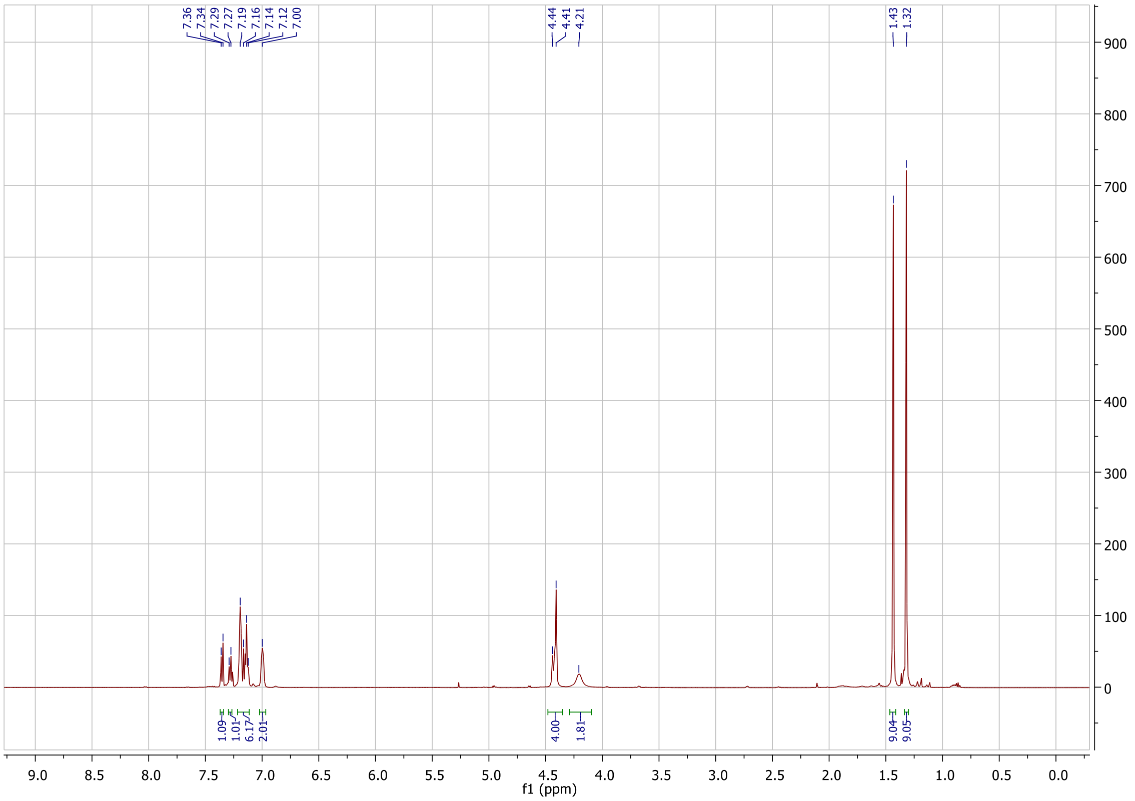

^{13}C NMR spectra of Tert-butyl 3-(N-benzyl-2-chlorobenzamido)-3-(tert-butylcarbamoyl)azetidine-1-carboxylate 6k

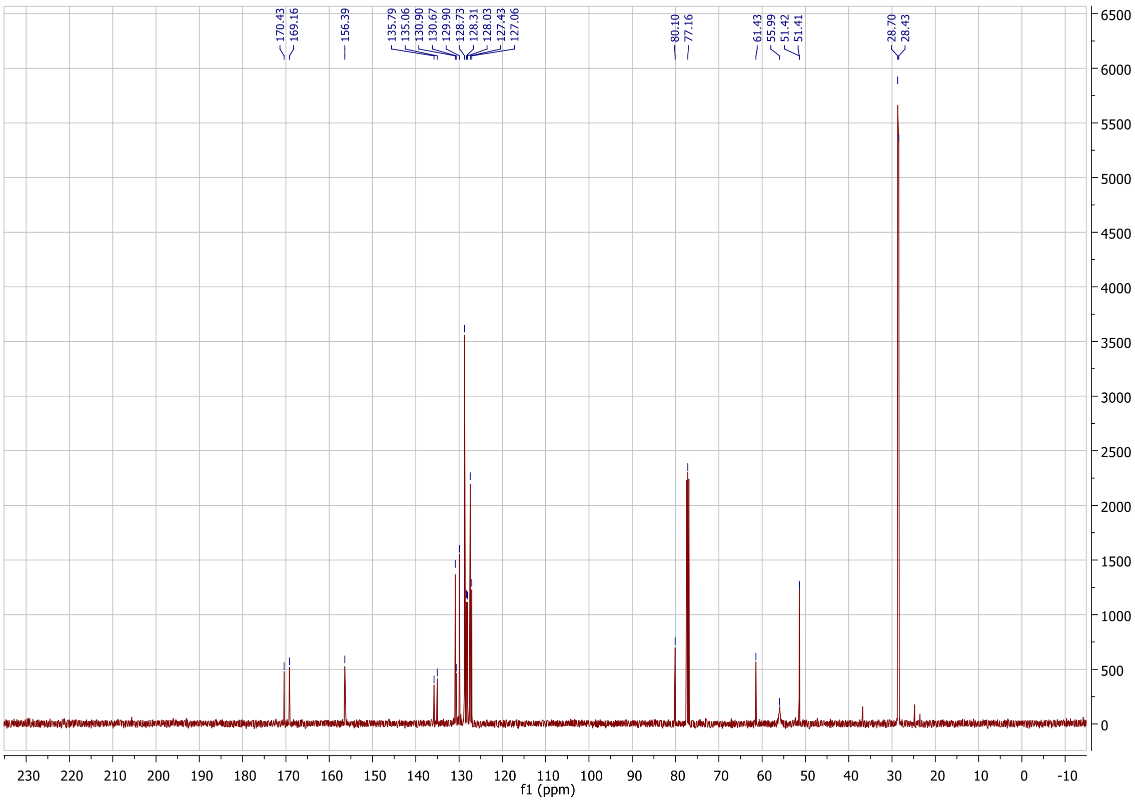

^{1}H NMR spectra of Tert-butyl 3-(N-(3,5-bis(trifluoromethyl)benzyl)-2-chlorobenzamido)-3-(tert-butylcarbamoyl)azetidine-1-carboxylate 6l

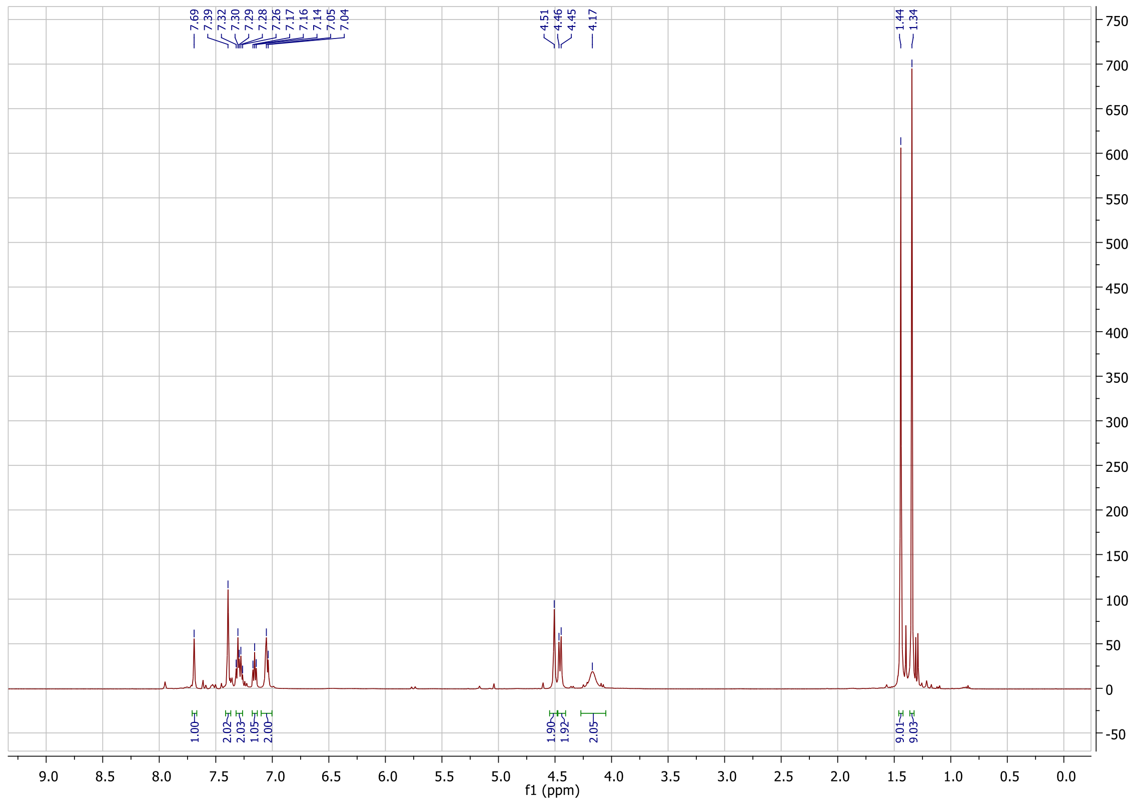

^{13}C NMR spectra of Tert-butyl 3-(N-(3,5-bis(trifluoromethyl)benzyl)-2-chlorobenzamido)-3-(tert-butylcarbamoyl)azetidine-1-carboxylate 6l

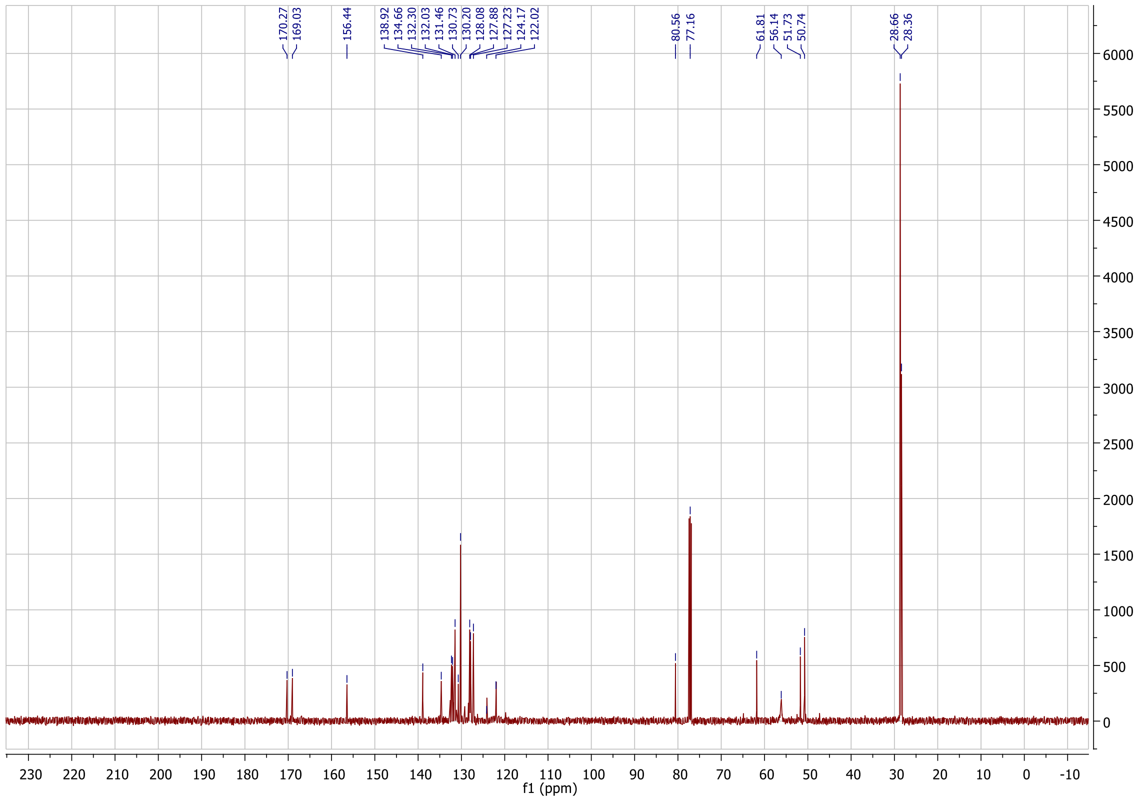

^{1}H NMR spectra of Tert-butyl 3-(tert-butylcarbamoyl)-3-(2-chloro-N-(thiophen-2-ylmethyl)benzamido)azetidine-1-carboxylate 6m

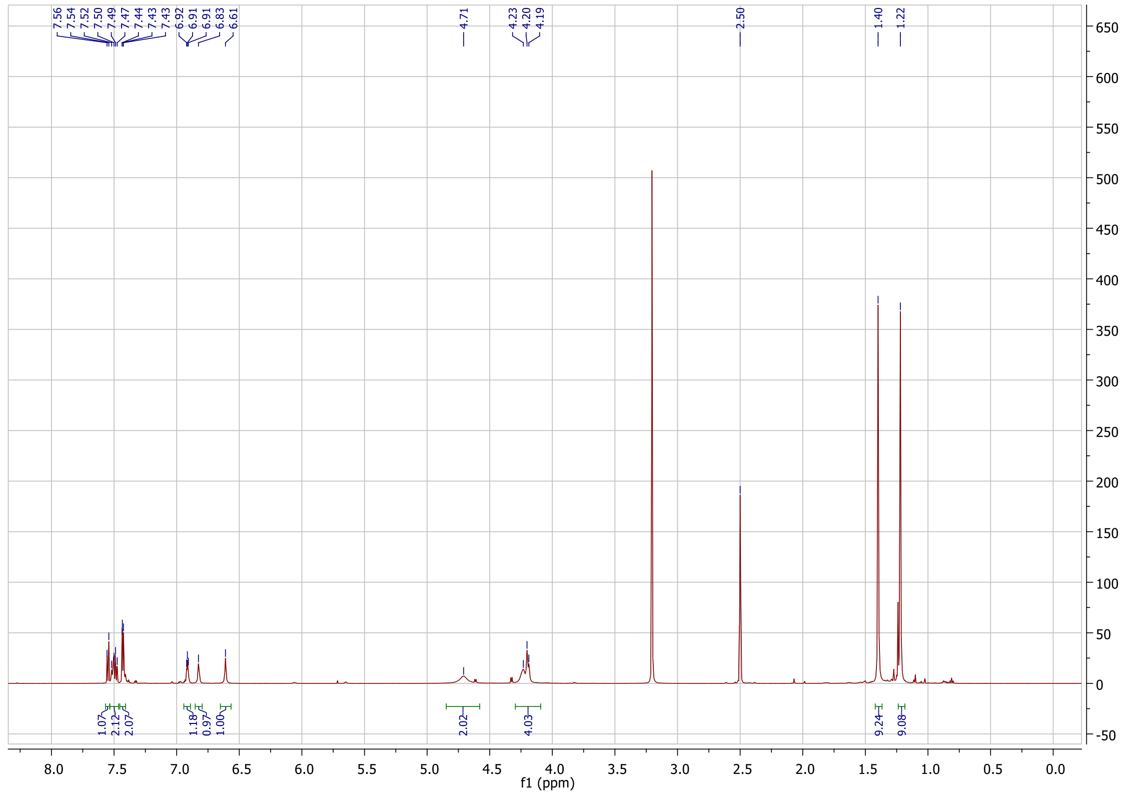

^{13}C NMR spectra of Tert-butyl 3-(tert-butylcarbamoyl)-3-(2-chloro-N-(thiophen-2-ylmethyl)benzamido)azetidine-1-carboxylate 6m

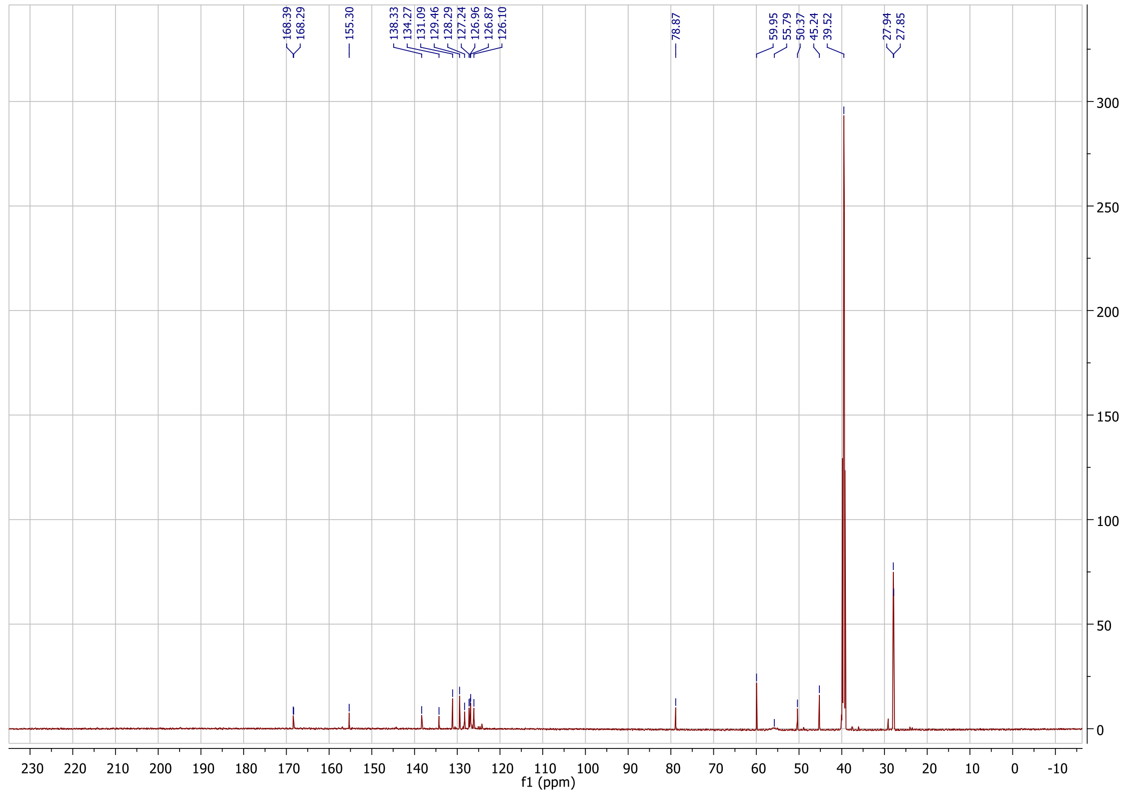

^1H NMR spectra of Tert-butyl 3-(tert-butylcarbamoyl)-3-(2-chloro-N-cyclohexylbenzamido)azetidine-1-carboxylate 6n

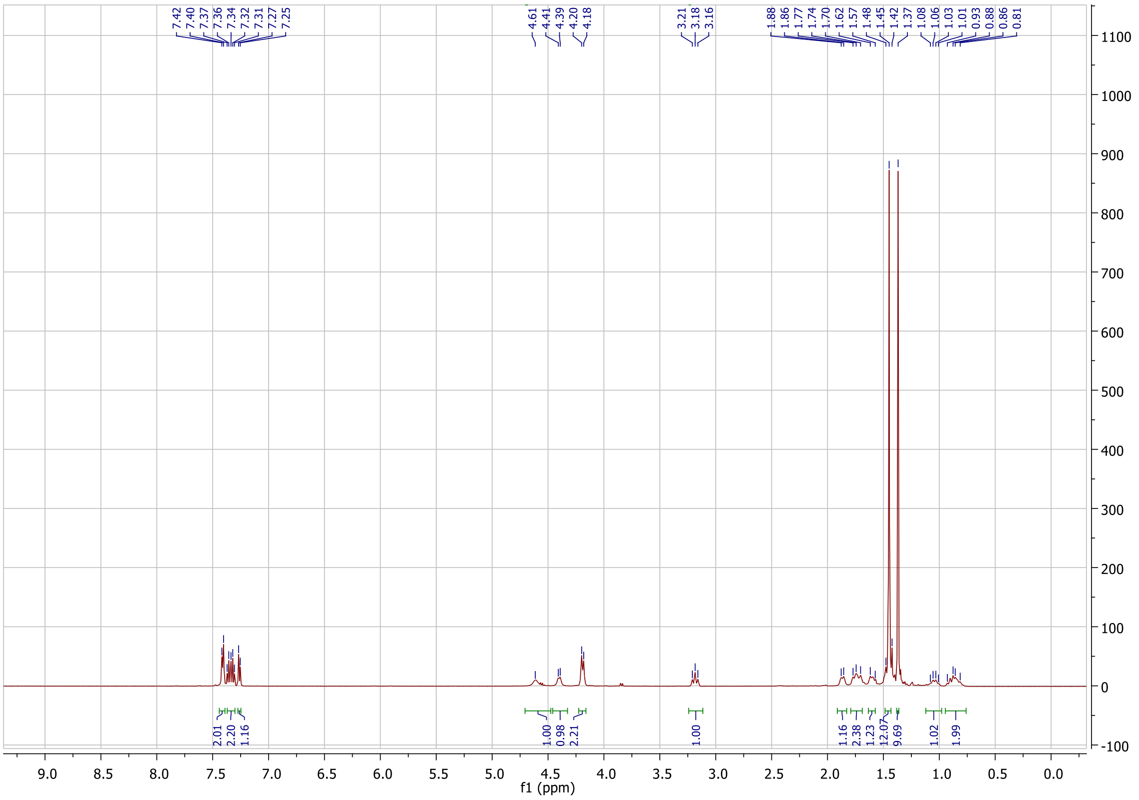

^{13}C NMR spectra of Tert-butyl 3-(tert-butylcarbamoyl)-3-(2-chloro-N-cyclohexylbenzamido)azetidine-1-carboxylate 6n

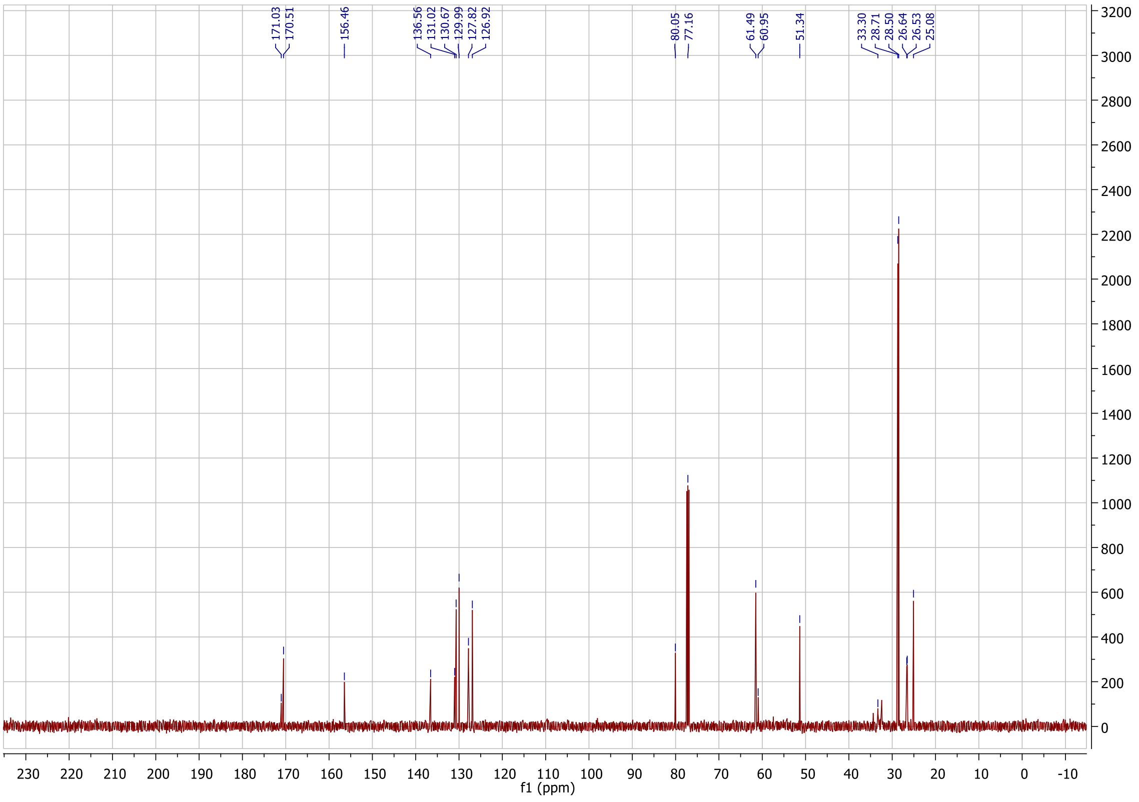

^{1}H NMR spectra of Tert-butyl 3-(tert-butylcarbamoyl)-3-(2-chloro-N-(prop-2-yn-1-yl)benzamido)azetidine-1-carboxylate 6o

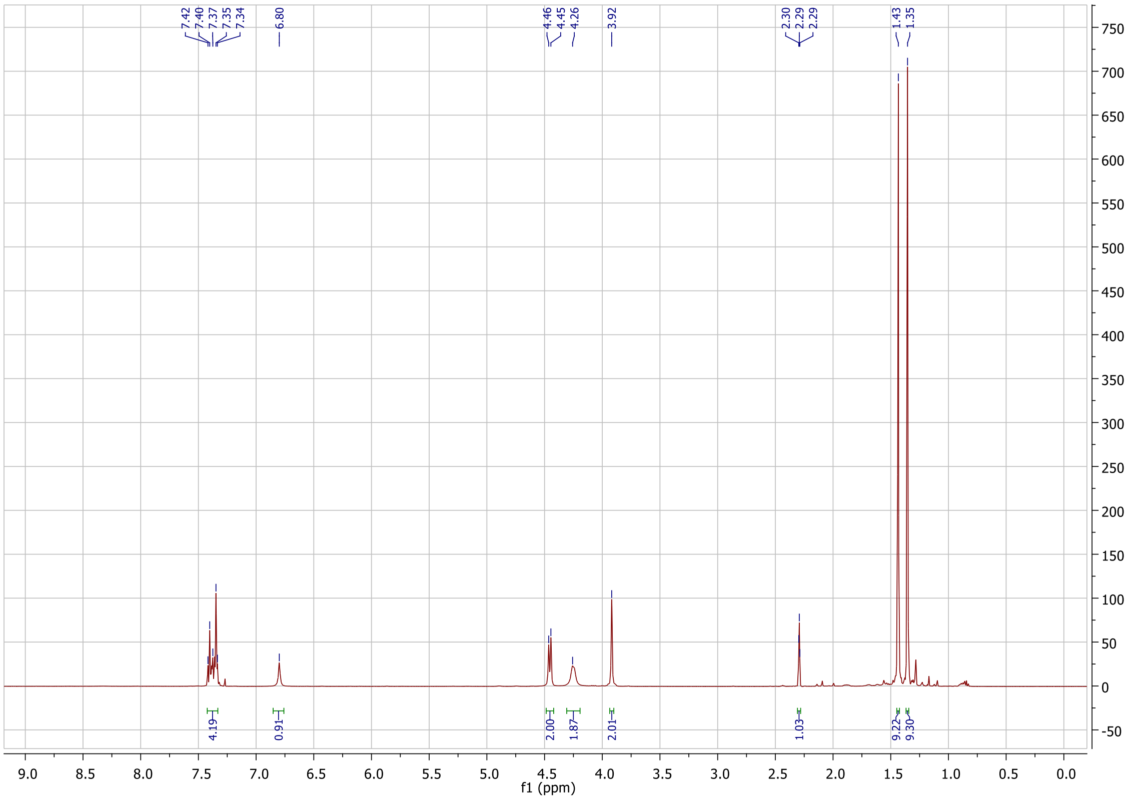

^{13}C NMR spectra of Tert-butyl 3-(tert-butylcarbamoyl)-3-(2-chloro-N-(prop-2-yn-1-yl)benzamido)azetidine-1-carboxylate 6o

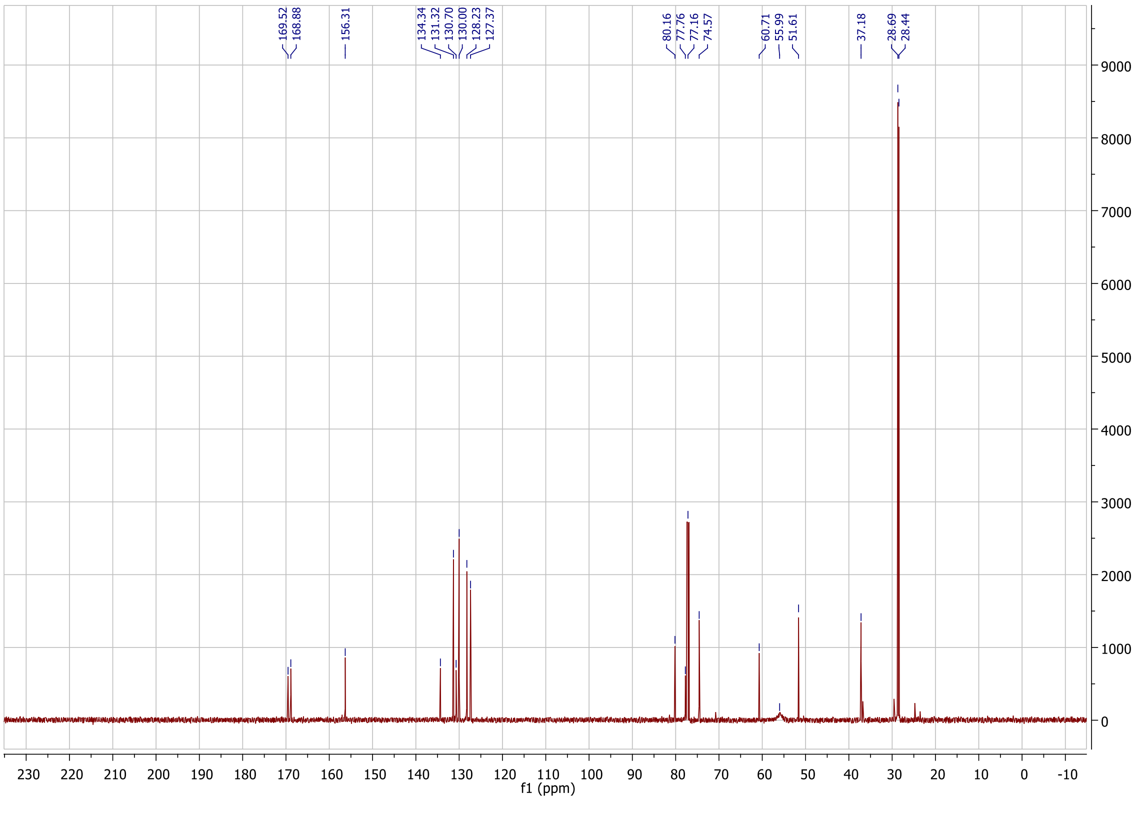

^{1}H NMR spectra of tert-butyl 3-(N-benzyl-2-chlorobenzamido)-3-((ethoxymethyl)carbamoyl)azetidine-1-carboxylate 7a

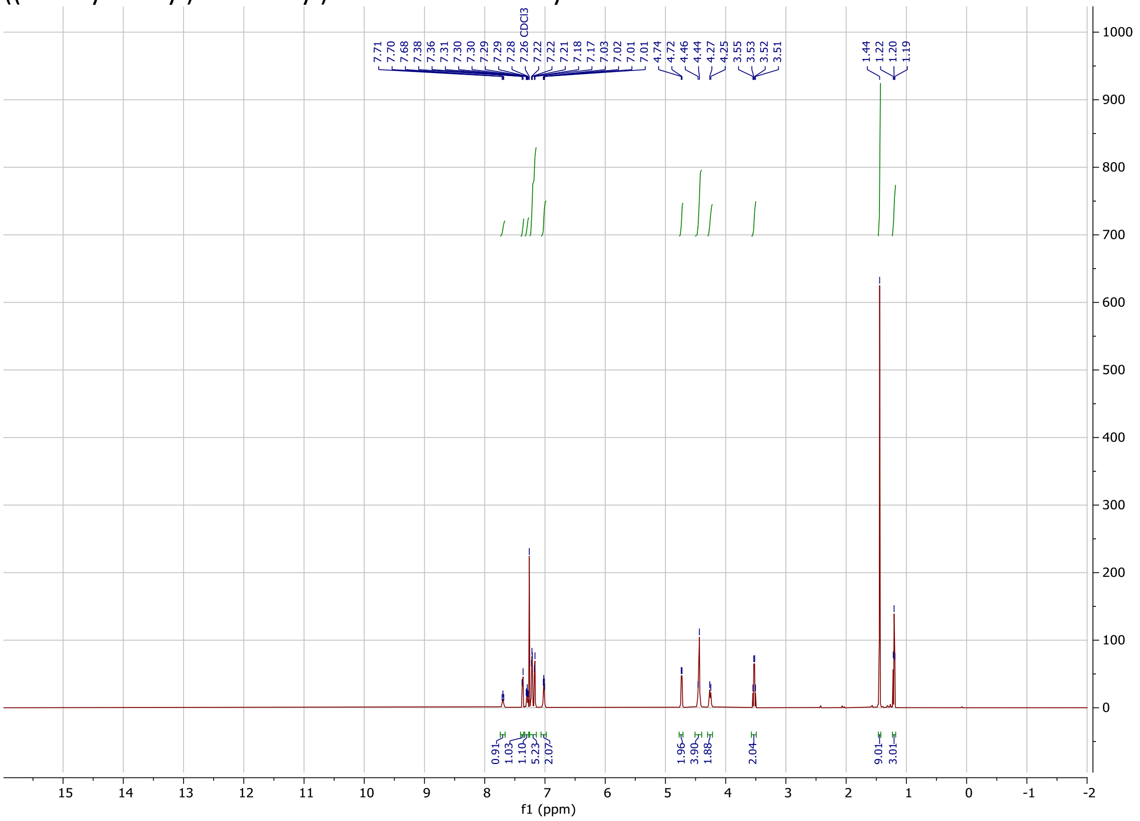

^{13}C NMR spectra of tert-butyl 3-(N-benzyl-2-chlorobenzamido)-3-((ethoxymethyl)carbamoyl)azetidine-1-carboxylate 7a

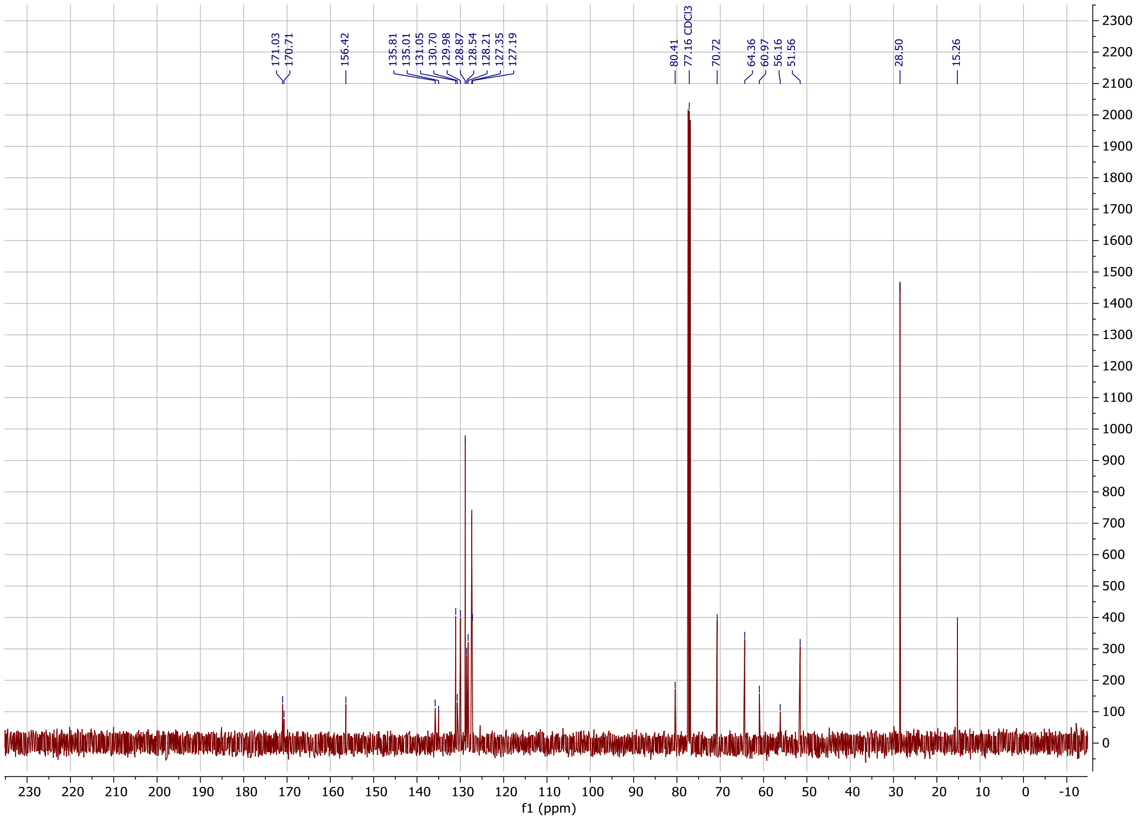

^{1}H NMR spectra of tert-butyl 3-(N-benzyl-2-chlorobenzamido)-3-(((methylthio)methyl)carbamoyl)azetidine-1-carboxylate 7b

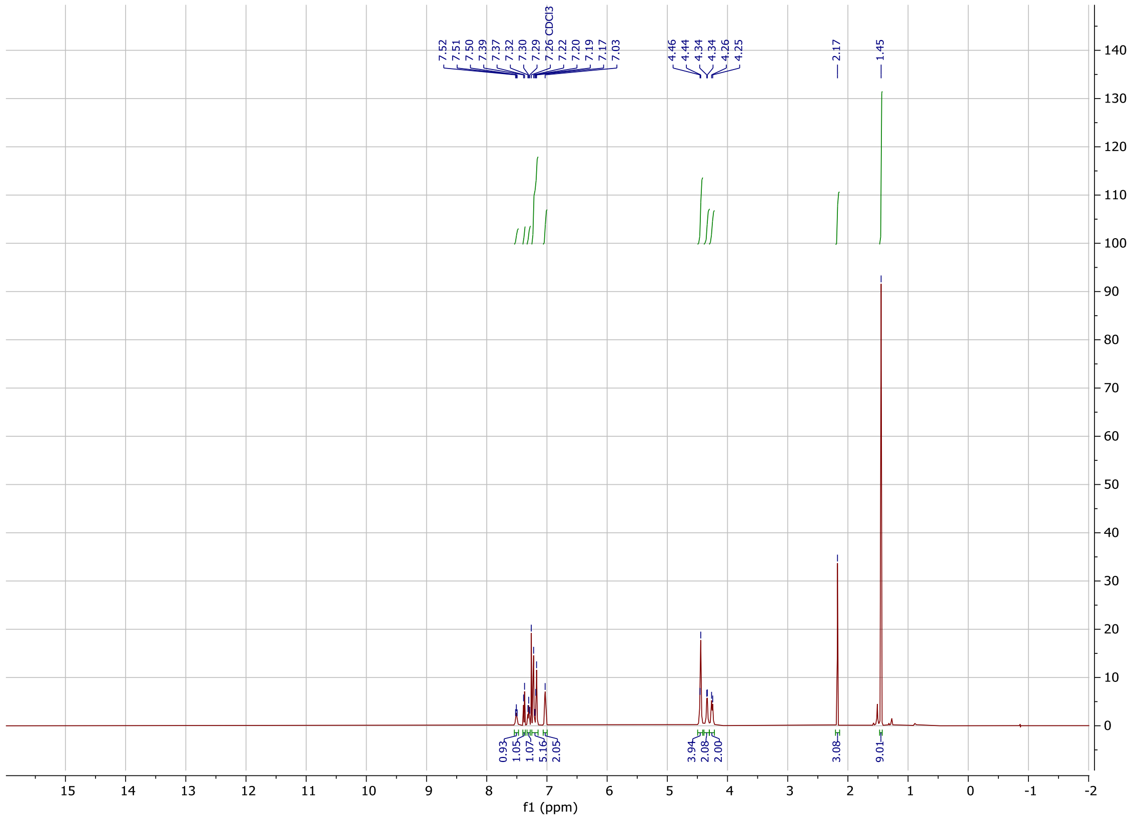

^{13}C NMR spectra of tert-butyl 3-(N-benzyl-2-chlorobenzamido)-3-(((methylthio)methyl)carbamoyl)azetidine-1-carboxylate 7b

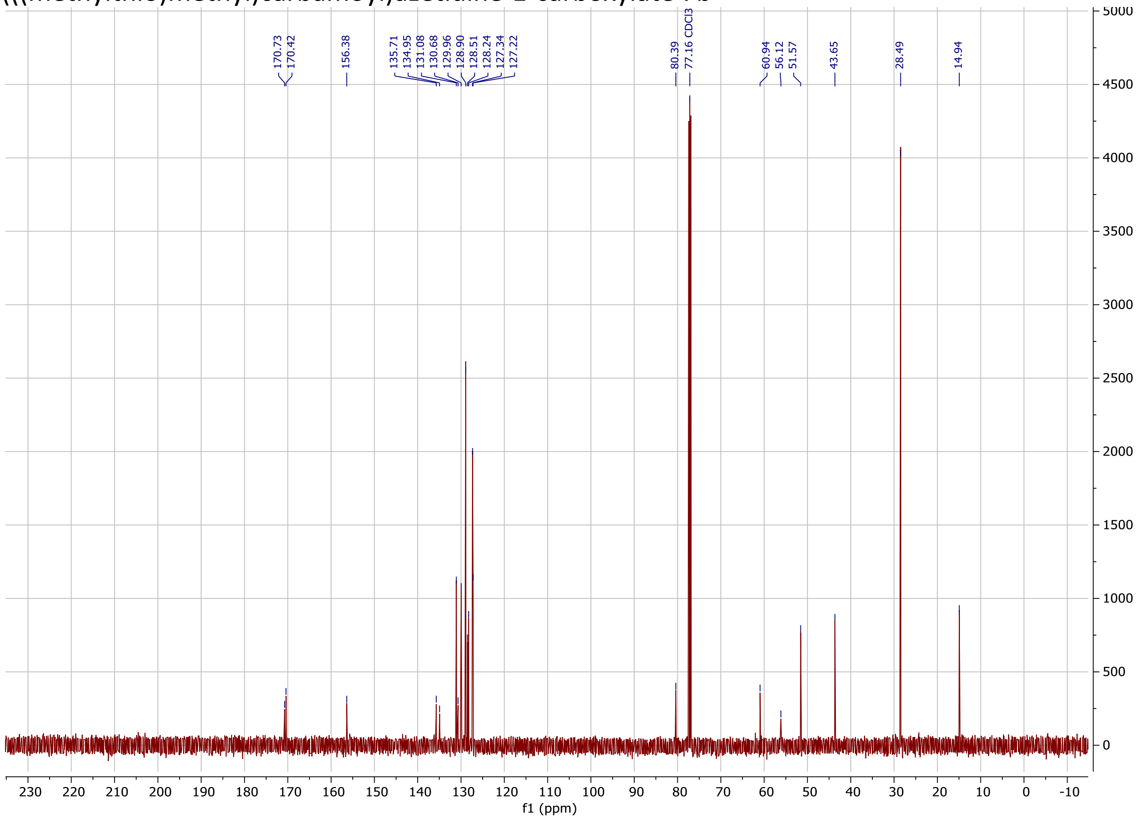

^{1}H NMR spectra of tert-butyl 3-(N-benzyl-2-chlorobenzamido)-3-((morpholinomethyl)carbamoyl)azetidine-1-carboxylate 7c

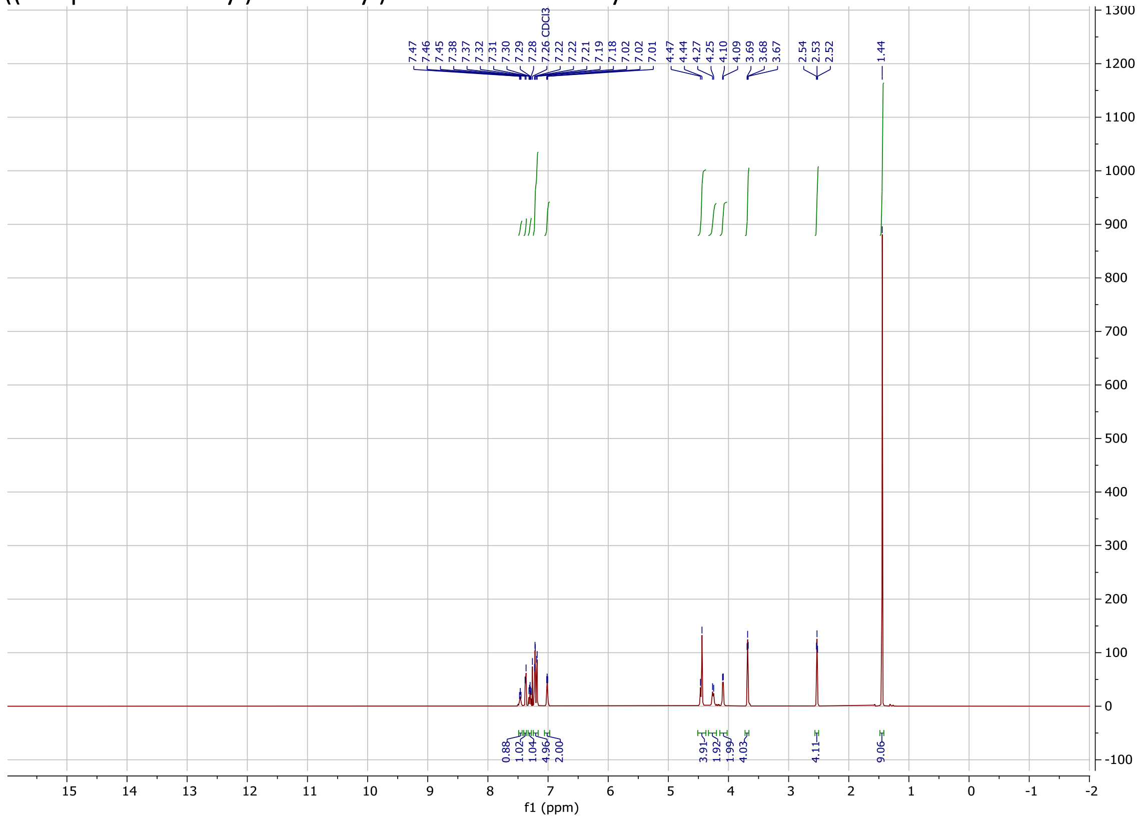

^{13}C NMR spectra of tert-butyl 3-(N-benzyl-2-chlorobenzamido)-3-((morpholinomethyl)carbamoyl)azetidine-1-carboxylate 7c

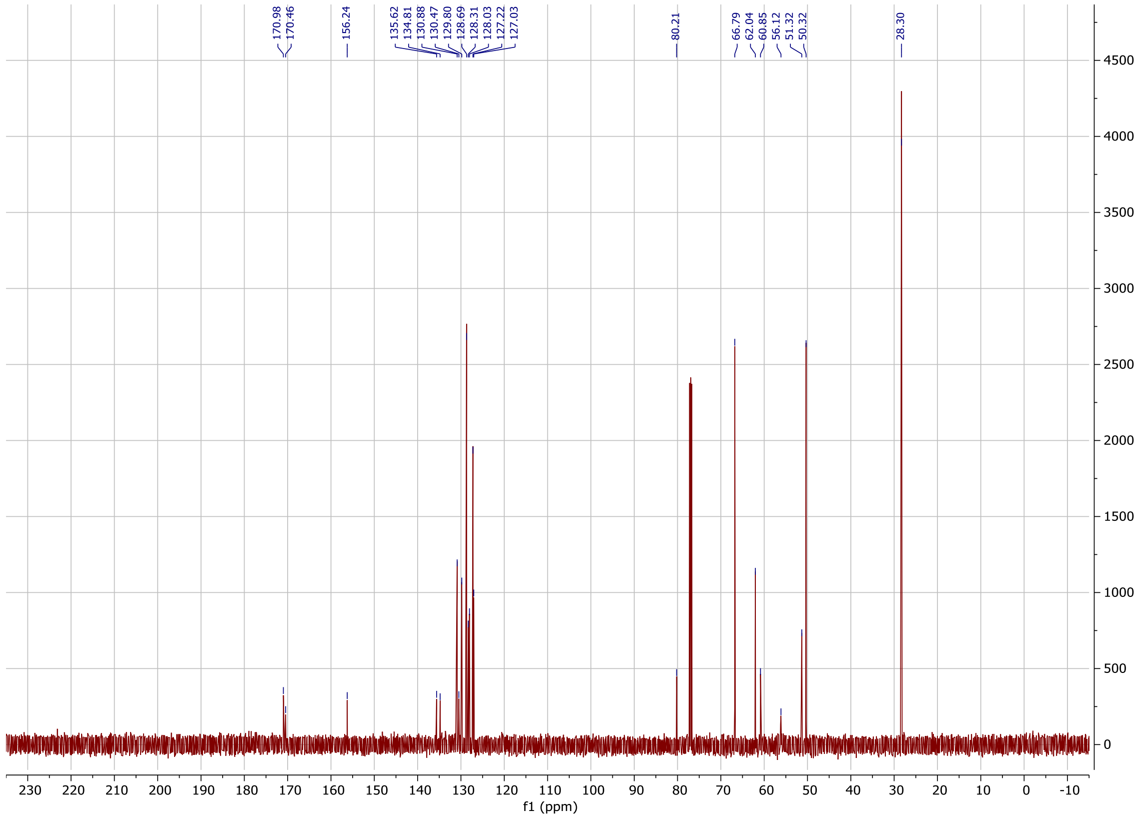

^{1}H NMR spectra of tert-butyl 3-(N-benzyl-2-chlorobenzamido)-3-(((1,3-dioxoisoindolin-2-yl)methyl)carbamoyl)azetidine-1-carboxylate 7d

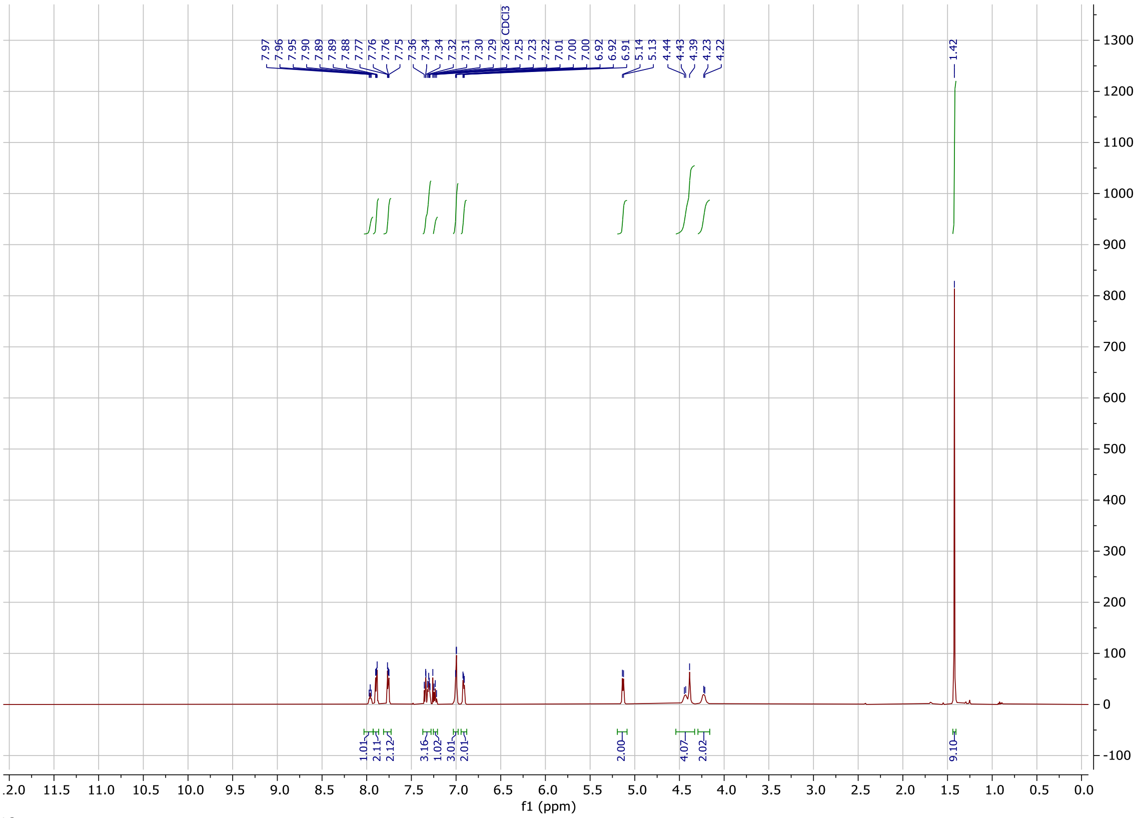

^{13}C NMR spectra of tert-butyl 3-(N-benzyl-2-chlorobenzamido)-3-(((1,3-dioxoisoindolin-2-yl)methyl)carbamoyl)azetidine-1-carboxylate 7d

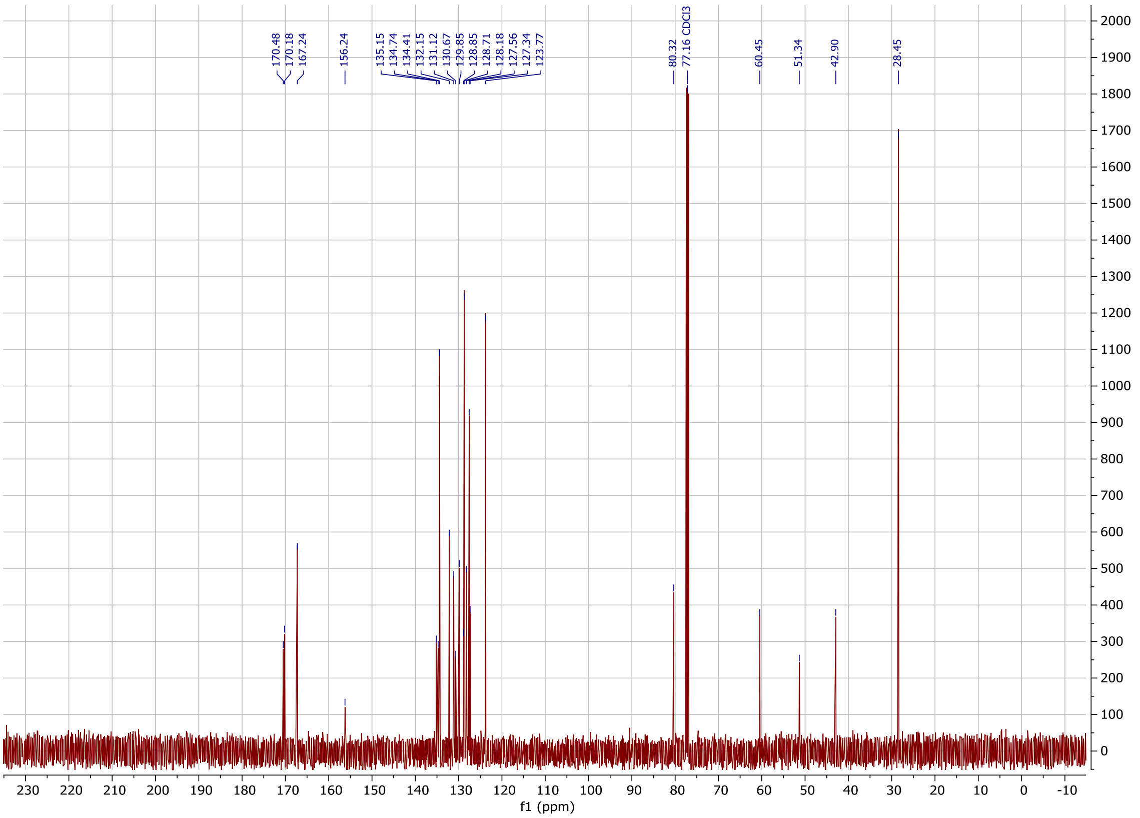

^{1}H NMR spectra of dimethyl 2-((3-(N-benzyl-2-chlorobenzamido)-1-(tert-butoxycarbonyl)azetidine-3-carboxamido)methyl)malonate 7e

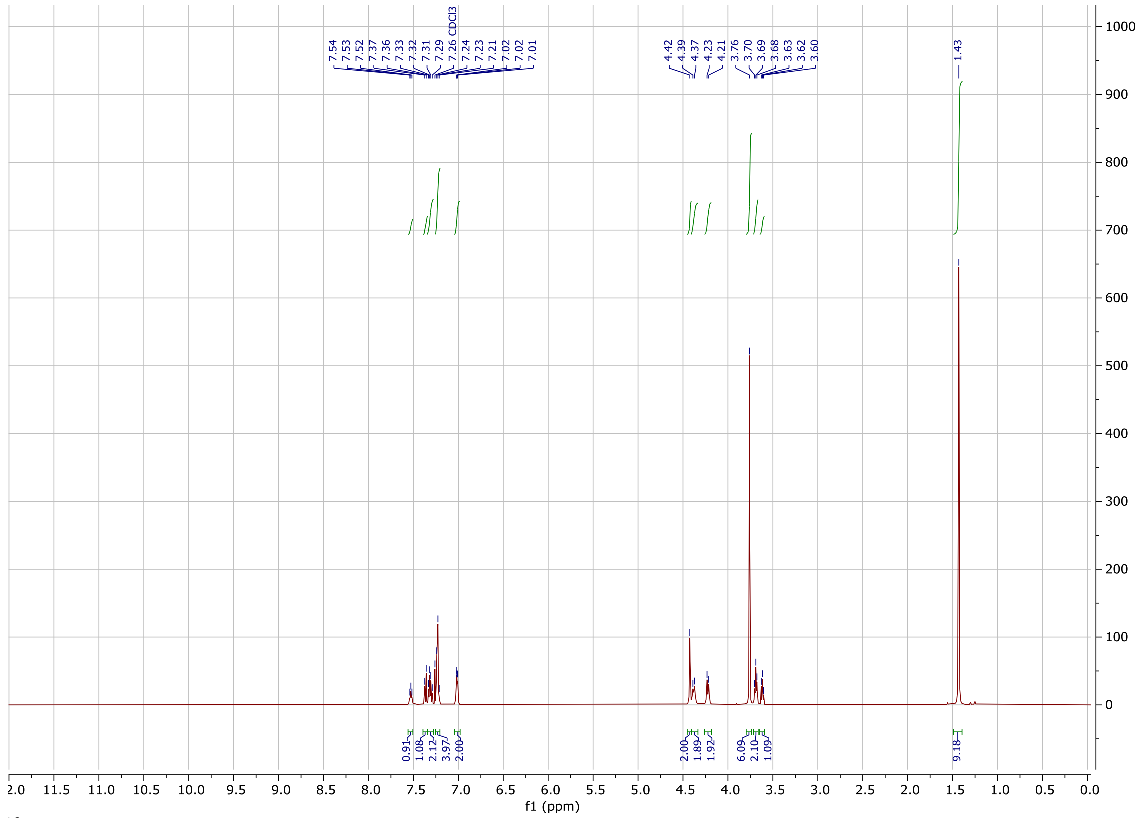

^{13}C NMR spectra of dimethyl 2-((3-(N-benzyl-2-chlorobenzamido)-1-(tert-butoxycarbonyl)azetidine-3-carboxamido)methyl)malonate 7e

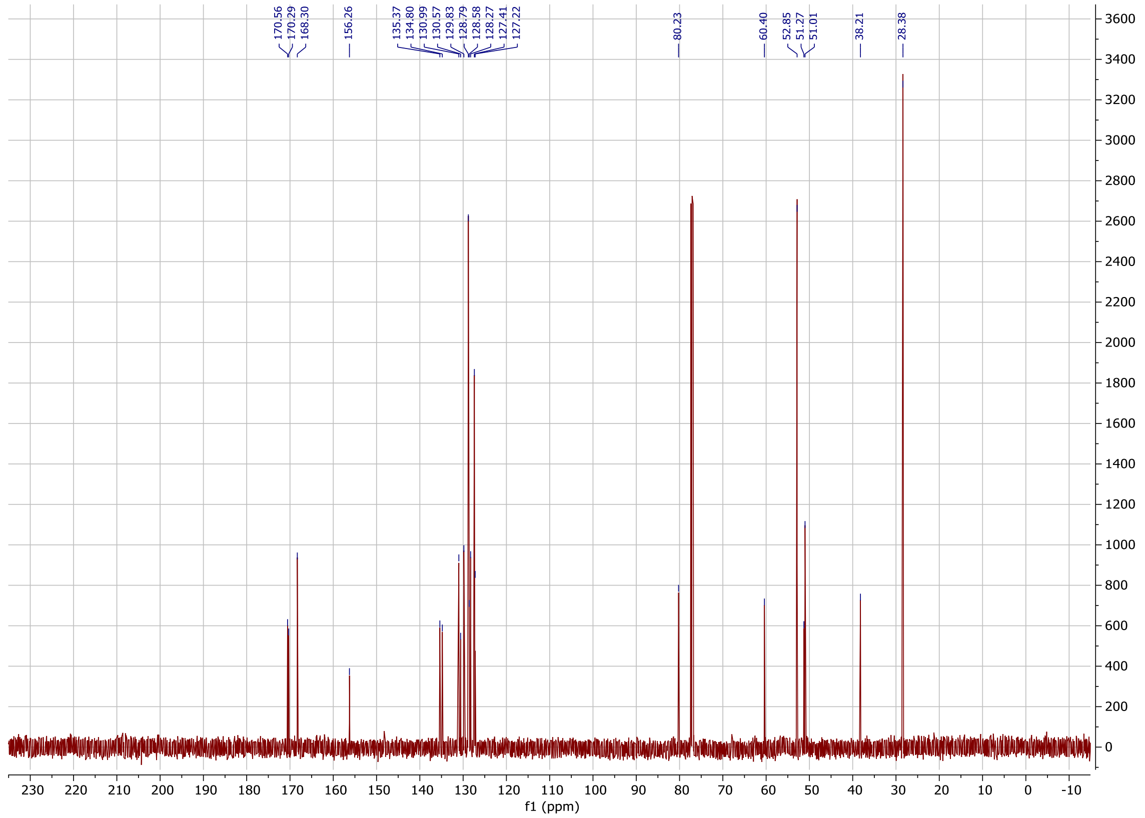

^{1}H NMR spectra of tert-butyl 3-(N-benzyl-2-chlorobenzamido)-3-((cyanomethyl)carbamoyl)azetidine-1-carboxylate 7f

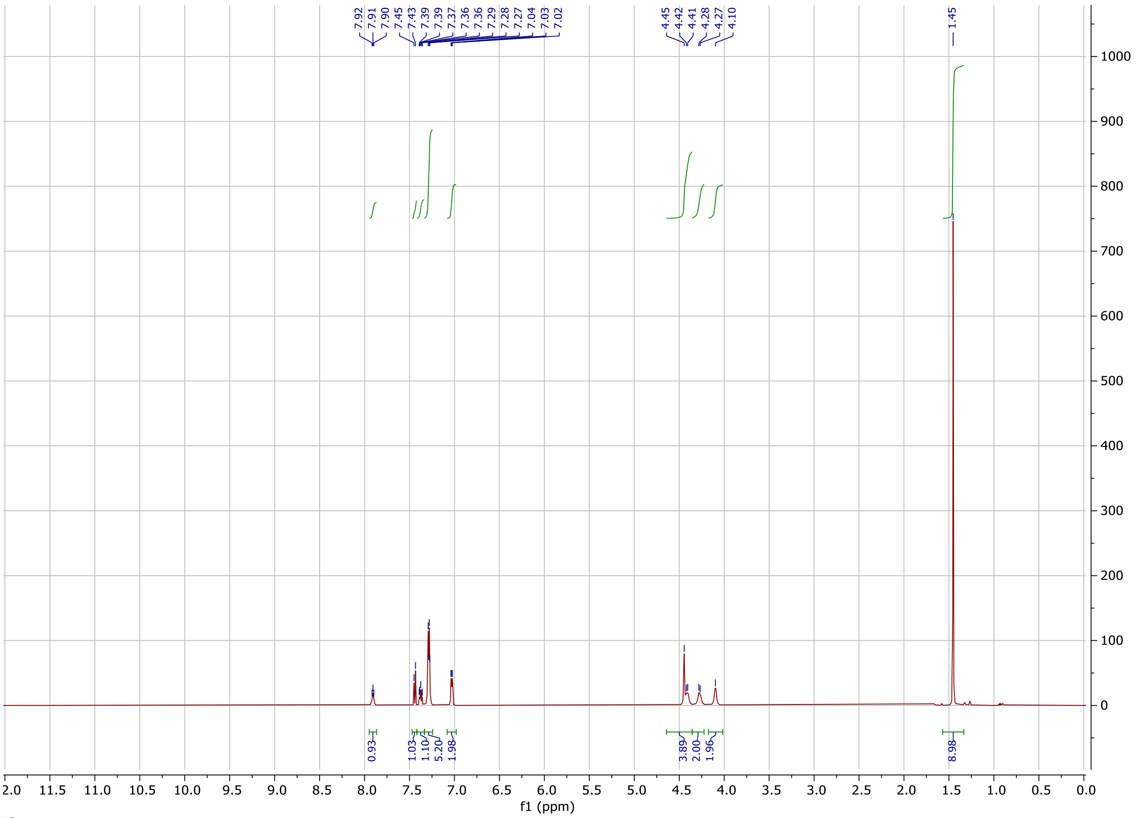

^{13}C NMR spectra of tert-butyl 3-(N-benzyl-2-chlorobenzamido)-3-((cyanomethyl)carbamoyl)azetidine-1-carboxylate 7f

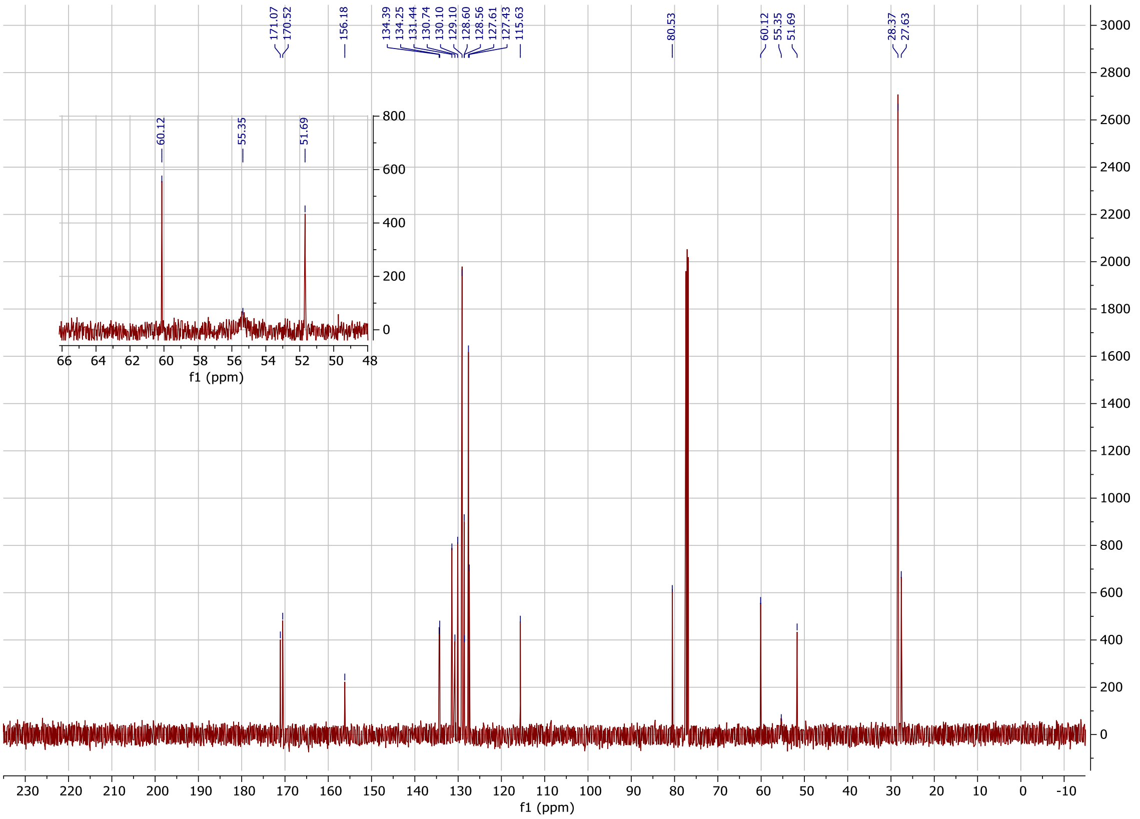

^{1}H NMR spectra of tert-butyl 3-(allylcarbamoyl)-3-(N-benzyl-2-chlorobenzamido)azetidine-1-carboxylate 7g

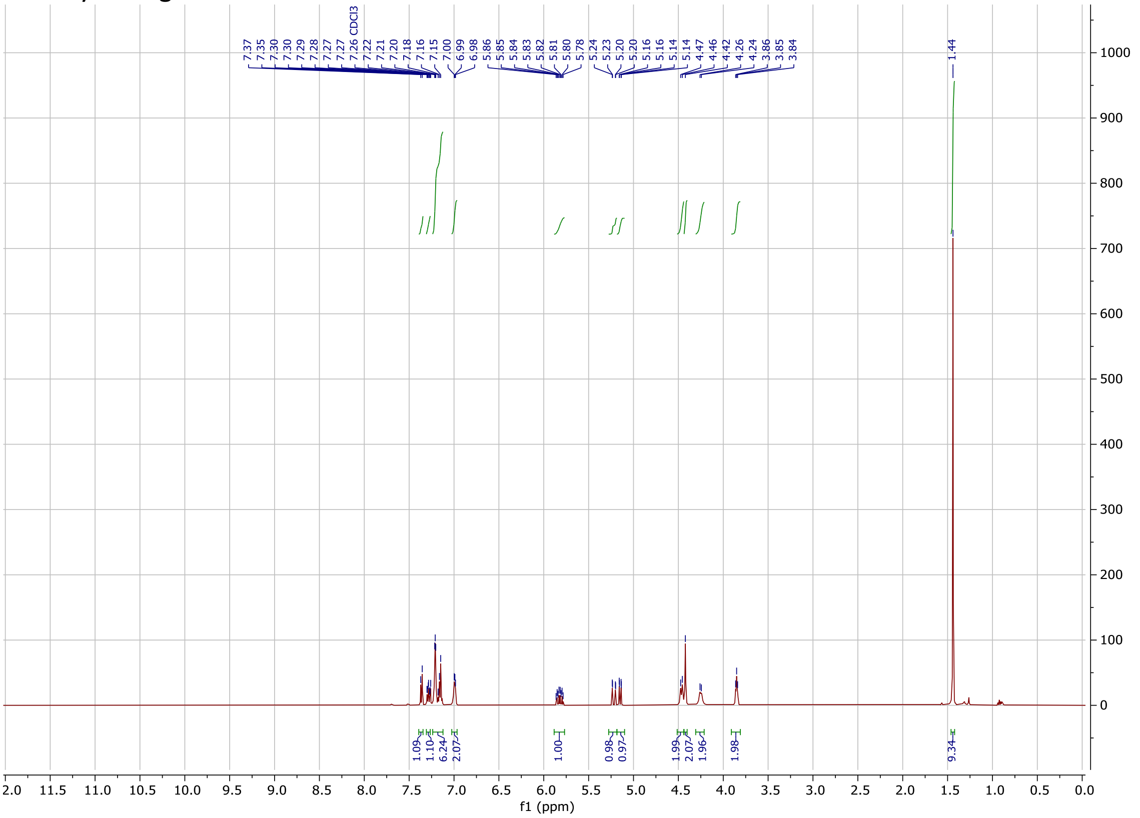

^{13}C NMR spectra of tert-butyl 3-(allylcarbamoyl)-3-(N-benzyl-2-chlorobenzamido)azetidine-1-carboxylate 7g

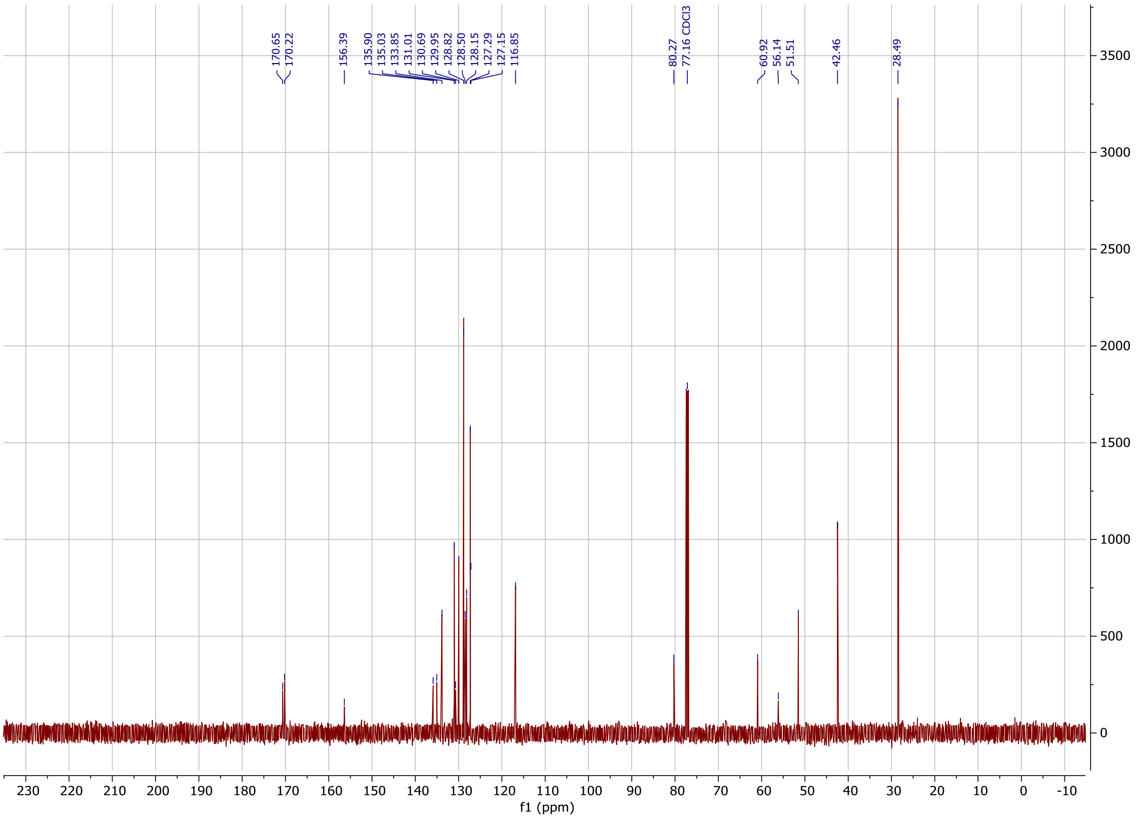

Supplement: Supplementary file 1 — Supporting Information [file OPEN-12-e202200083-s001.pdf]
